# Supplementary material for: Evidence for a transgenerational mutational signature from ionizing radiation exposure in humans
Source: Sci Rep. 2025 Jun 23;15:20262. doi: 10.1038/s41598-025-07030-5 (PMC12185716; doi:10.1038/s41598-025-07030-5)
Supplement: Supplementary file 3 — Supplementary Material 3. [file 41598_2025_7030_MOESM3_ESM.pdf]

BAIUDBw

Strahlenmessstelle der Bundeswehr

Bericht-Nr.: S 209/20

Retrospektive Dosisberechnung Röntgenstörstrahlung

Exposition gegenüber Röntgenstörstrahlung an Radar- und Feuerleitgeräten der ehemaligen NVA

07.07.2021

Munster

Verfasser: RDir Dr. SCHIRMER, Andreas
Datum: 07.07.2021

Strahlenmessstelle der Bundeswehr
Bundesamt für Infrastruktur, Umweltschutz und Dienstleistungen der Bundeswehr

29633 Munster, den 07.07.2021
Humboldtstr. 1
Tel: 05192/ 136 -347, -538, FAX-506
BwFw: 90-2252-347, -538, FAX -506
StrahlenmessstelleBw@bundeswehr.org

Az 87 - 14 – 24

Bericht-Nr.: S 209/21

Hinweise zur Körperdosis ehemaliger Beschäftigter durch Röntgenstörstrahlung an Radargeräten der ehemaligen Nationalen Volksarmee

1 Auftrag, Vorbemerkung

Auftragsgemäß legt die Strahlenmessstelle der Bundeswehr das Ergebnis der Dosisberechnung für Beschäftigte der ehemaligen Nationalen Volksarmee als Beitrag zur Studie des Instituts für Genomische Statistik und Bioinformatik, Universitätsklinikum Bonn, Rheinische Friedrich-Wilhelms-Universität Bonn vor.

Im Bericht des Arbeitsstabes Dr. Sommer (‚Die Bundeswehr und ihr Umgang mit Gefährdungen und Gefahrstoffen – Uranmunition, Radar, Asbest‘ [1]) vom 21.06.2001 war zur Bearbeitung der damals zahlreichen Anträge ehemaliger Beschäftigter auf Anerkennung von Gesundheitsschäden als berufsbedingt (Wehrdienstbeschädigungsverfahren) im Rahmen des dafür geltenden geordneten Verfahrens der Anerkennung einer Berufskrankheit (hier: BK 2402 - Erkrankungen durch ionisierende Strahlen) eine Beurteilung der Ursächlichkeit einer Gesundheitsschädigung unabdingbar. Grundlage für eine Betrachtung der Ursächlichkeit ist die rezipierte Körper-, Teilkörper oder Organdosis. Für die Schaffung der Beurteilungsgrundlage wurden im Rahmen einer Arbeitsgruppe (Arbeitsgruppe Aufklärung der Arbeitsplatzverhältnisse Radar-AG Radar) gerätespezifisch die Arbeitsplatzverhältnisse an Radarsystemen hinsichtlich der Einwirkung ionisierender Röntgenstörstrahlung untersucht und dokumentiert, um tätigkeitsspezifische Dosiswerte bereitzustellen, anhand derer in den Einzelfällen die Ersatzdosis festgelegt werden kann.

Maßgabe dieser Untersuchungen war eine in [1] geforderte Großzügigkeit in der Ermittlung der Arbeitsplatzverhältnisse. Sie wurde –da in der Beauftragung der AG Radar keine Quantifizierung dieser Vorgabe erfolgte- umgesetzt durch:

(i) Ansetzen des Maximalwertes bei Vorliegen einer Messreihe von Werten der Ortsdosis/Ortdosisleistung für ein bestimmtes Radargerät für alle Arbeitsplätze an Geräten des betreffenden Typs,

(ii) Ansatz weit gefasster Arbeitszeiten in den Bereichen mit Exposition gegenüber Röntgenstörstrahlung mit ebenfalls weitgreifender Unterstellung von Betriebszuständen (z.B. für Inspektionszwecke geöffnete Geräteklappen, die eine Abschirmung der Röntgenstörstrahlung bewirken).

Mit (i) wurde also auf die Analyse ggf. vorliegender Verteilungen von Dosiswerten verzichtet und die resultierende 'Instabilität' der Ergebnisse gegenüber notwendiger Revisionen bei Bekanntwerden höherer Messwerte für ein Radargerät bewusst in Kauf genommen. Bei der Bemessung der Arbeitszeiten in (ii) bildeten die Befragungen von ehemaligen Technikern – auch aus dem Kreis von Antragstellern- die Grundlage. Auch hier wurden teilweise Arbeitsweisen und Arbeitszeiten im Expositionsbereich ohne Analyse der Angaben zur Dauer oder Notwendigkeit, diese Arbeiten unter möglichen Expositionsbedingungen (also etwa bei angeschalteter Hochspannung) durchzuführen, in die Auswertung übernommen

Für die hier anzustellende Dosisbetrachtung sind die Maßgaben (i), (ii) nicht mehr adäquat. Beim Rückgriff auf Ergebnisse der AG Radar sind ggf. vorliegende Messwerte statistisch zu bewerten und es ist zu versuchen, die Unsicherheit in der Bemessung der Arbeitszeiten im Expositionsbereich zu erfassen.

Für den hier vorgelegten ersten Teil der Radargeräte der ehemaligen NVA wurden insgesamt 7 Radargeräte als expositionsrelevant identifiziert. In Abschnitt 2 werden diese Systeme kurz beschrieben. In Abschnitt 3 werden die für das hinsichtlich einer Exposition gegenüber Röntgenstörstrahlung ‚qualifizierte' Personal die monatlichen Werte der rezipierten Körperdosis angegeben. Die Einzelergebnisse werden in Abschnitt 4 zusammengefasst, die auf Probanden bezogenen Einzelergebnisse befinden sich im Anhang

2 Kurzbeschreibung der Systeme und Expositionssituationen

Für das sich aus der ehemaligen NVA rekrutierende Probandenensemble wurden folgende Radargeräte (auch als Funkmessgeräte bezeichnet) identifiziert:

1. Rundblickstation (RBS) P-15
2. Rundblickstation (RBS) P-19
3. Rundblickstation (RBS) P-35
4. Rundblickstation (RBS) P-37
5. Geschützrichtstation GRS-9
6. Höhenmessradar PRW 11
7. Flugabwehrraketensystem S-75

Im Folgenden werden die Radargeräte und die für die daran Beschäftigten relevanten Expositionssituationen, wie sie sich aus den Arbeitsplatzverhältnissen ergeben, dargestellt.

2.1 Rundblickstation P-15 und P-19

Die Rundblickstationen des Typs P-15 waren bei allen Waffengattungen der NVA in Nutzung. Die kompakten, transportablen Radargeräte sind in Abb. 1 dargestellt, Sie wurden Ende der 1950-er Jahre eingeführt, es gab im Laufe der Nutzungszeit mehrere Modifikationen. Sie betrafen vorrangig den Empfängertrakt und den Störschutz; Modulator und Sender mit den Röntgenstörstrahlern unterlagen keinen wesentlichen technischen Änderungen.

Die mobilen Rundblickstationen des Typs P-19 wurden ab Ende der 1970-er Jahre in der NVA eingeführt, wo sie für gleiche Aufgaben wie die P-15 übernahmen.

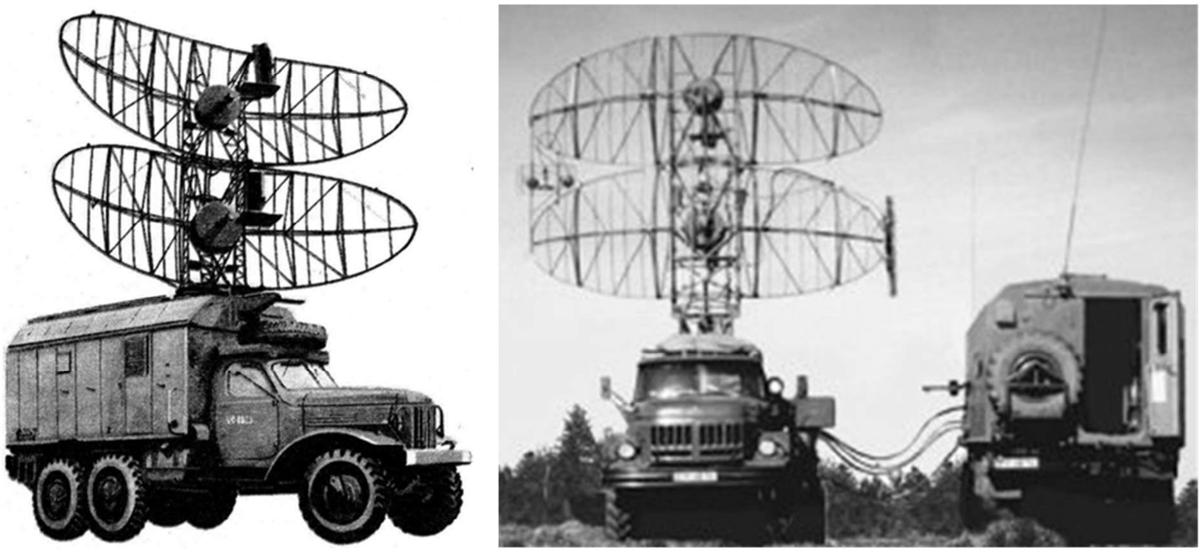

Abb. 1: Ansichten der mobilen Rundblickstationen P-15 (links) und P-19 (rechts) in entfalteten, betriebsbereiten Zustand

Abb. 2 zeigt die Anordnung der Gerätekomponenten der P-15 im Kofferaufbau des Lkw-Grundfahrzeugs. Der Kofferaufbau ist in einen Teil mit der Antenne und einer Kabine mit der übrigen Technik und der Konsole des Radaroperators (Funkorters) aufgeteilt. Der Arbeitsplatz des Operators befand sich bei der P-15 also in unmittelbarer Nachbarschaft der Gerätegruppen mit den Röntgenstörstrahlern.

Bei der P-19 waren die Antennenanlage und der Kofferaufbau mit Radarsender und Empfänger mit den Sichtgeräten auf zwei Trägerfahrzeugen untergebracht (Abb. 1, rechts). Auch bei der P-19 befand sich der Arbeitsplatz des Operators unmittelbar neben der Sender/Modulatorbaugruppe., allerdings waren bei der P-19 die räumlichen Verhältnisse weniger beengt.

Die Röntgenstörstrahler sind in Tabelle 1 angegeben. Hier und bei der Dosisbetrachtung von Röntgenstörstrahlern bei allen weiteren Radargeräten kann auf die Erfassung von Bildröhren an den Konsolen der Operatoren verzichtet werden, da durch Kathodenstrahlröhren keine Dosisbeiträge entstehen[1].

[1] Die in Sichtgeräten eingebauten Kathodenstrahlröhren sind wegen Betriebsspannungen über 5 kV überwiegend ebenfalls Röntgenstörstrahler i.S. der Röntgenverordnung (heute: Strahlenschutzgesetz). Aus konstruktiven Gründen sind Bildröhren als große Vakuumbehälter insbesondere an der bilddarstellenden Seite als dickwandige

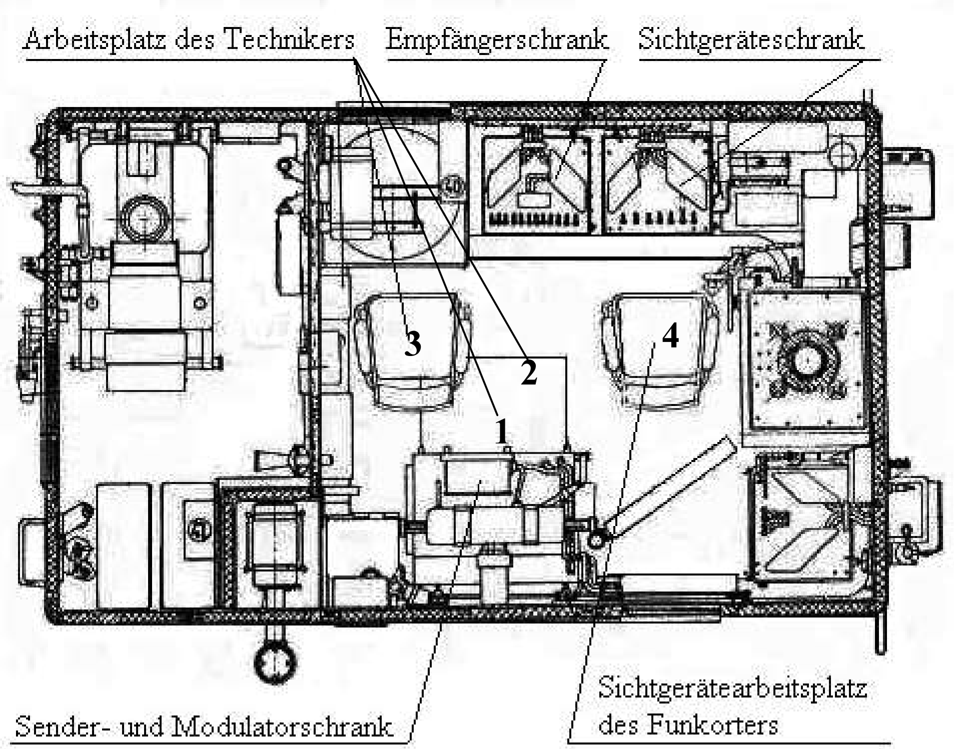

Abb. 2: Anordnung der Geräteblöcke im Kofferaufbau der P-15 und der Arbeitsplätze des Technikers und des Funkorters (Die Zahlen bezeichnen die üblichen Arbeitsplätze, von denen einige Aufpunkte für die Dosisbetrachtung nach Tabelle xx sind.)

Tabelle 1: Einbauorte und technische Daten der Röntgenstörstrahler in den RBS P-15 und RBS P-19

Zeile Nr.	RBS	Baugruppe	Bauteil	Anzahl	Typ	max. Betriebs-spannung
1	P-15	Sender	Magnetron	1	MI-119	27 kV
2		Modulator	Schalttetrode	2	GMI-90	27 kV
3		Hochspannungs-gleichrichter	Gleichrichterröhren	4	W1-0,1/30	14 kV
4	P-19	Sender	Magnetron	1	MI 119	27 kV
5		Modulator	Schalttetrode	2	GMI 90	27 kV

Maßgeblich ist die an den Schalttetroden des Typs GMI-90 herrschende Ortsdosisleistung. Durch die Ausführung des Sender- und Modulatorschrankes als massive Stahlblechkonstruktion wird die weiche Röntgenstörstrahlung ausreichend abgeschirmt, sodass nur kurze Zeitabschnitte für Kontrollen bei geöffneten Gerätetüren expositionsrelevant sind. Bei der Messung an einer dazu wieder in Betrieb genommenen P-15 wurde allerdings eine teilweise deformierte Gerätetür vorgefunden, bei der an dem entstandenen Schlitz eine deutliche Erhöhung der Ortsdosisleistung beobachtet wurde.

Glasbehälter ausgelegt, der am Ort des Betrachters (Operators) die vollständige Abschirmung der Röntgenstörstrahlung bewirkt.

Bei beiden Rundblickstationen waren die Arbeitsplätze der Operatoren (Funkorter), also die Konsolen mit den Sichtgeräten im Kofferaufbau zusammen mit der Senderbaugruppe untergebracht. Abb. 2 zeigt die räumlichen Verhältnisse in der Kabine der RBS P-15. Die räumlichen Verhältnisse in der Kabine der RBS P-19 waren weitgehend ähnlich, durch den Aufbau der Antennenanlage auf einem eigenen LKW waren sie jedoch weniger beengt.

Zu den Werten der Ortsdosisleistung und den an Aufenthaltspunkten daraus abgeleiteten Werten der Ortsdosis wird auf Abschnitt 3.1. verwiesen.

2.2 Rundblickstationen P-35 und P-37

Die Rundblickstationen P-35 und P-37 wurden zur Luftraumüberwachung und zur Führung von Luftfahrzeugen im Rahmen der Flugabwehr eingesetzt. Die RBS des Typs P-35 wurden ab Anfang der 1960er Jahre bei der NVA eingeführt und ab der Mitte der 1970er Jahre durch RBS des Typs P-37 ersetzt. Der technische Aufbau der Sender-Modulatorbaugruppen mit der Antennenanlage war weitgehend gleich. Die Arbeitsplatz- und Dosisbetrachtung kann auf die P-37 beschränkt werden, die Ergebnisse sind auf die P-35 übertragbar. Abb. 3 zeigt die Sende-Empfangskabine der RBS P-37 mit den beiden fest daran montierten Antennen. Zur Erfassung des Azimuts rotierte die gesamte, auf einer Lafette installierte Kabine. Die Bedienung der Anlage erfolgte ferngesteuert von einer auf einem LKW montierten Kabine aus. Dort waren auch die Arbeitsplätze der Operatoren.

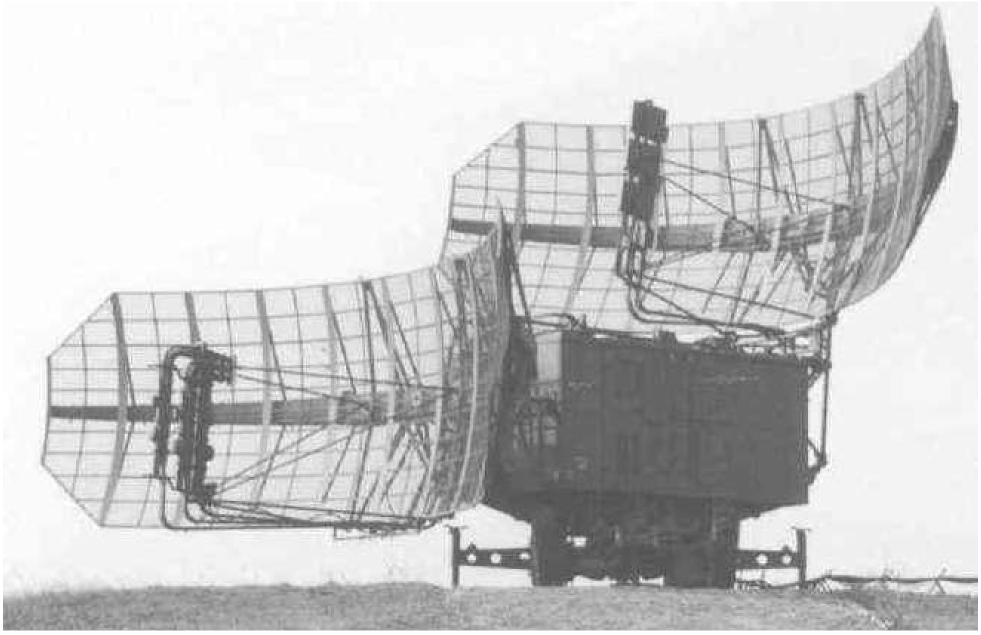

Abb. 3: Sende-/Empfangskabine der RBS P-37 (Die untere Antenne gehört zum 1. bis 3. Sende-/Empfangstrakt, die obere zum 4. und 5. Sende-/Empfangstrakt).

In der in Abb. 4 dargestellten Sende/Empfangskabine waren insgesamt 5 baugleiche –aber auf verschiedenen Frequenzen arbeitende- Sende-/Empfangsanlagen untergebracht. In Tabelle 2 sind Art und Typ der jeweils in einem Sender verbauten Röntgenstörstrahler angegeben.

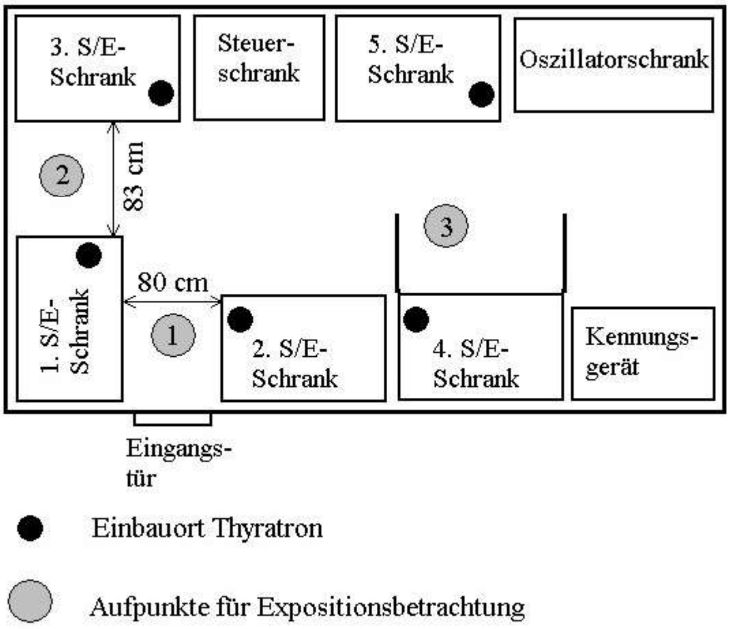

Abb. 4: Anordnung der Geräteschränke in der Sende-/Empfangskabine mit Angabe der Punkte, für die Expositionsbetrachtungen vorzunehmen sind (Für den 4. Sender-/Empfänger (S/E)-Schrank ist die Stellung der geöffneten Schranktüren skizziert.)

Tabelle 2: Röntgenstörstrahler der RBS P-37

Nr	Einbauort	Bauteil	Typ	Betriebs-spannung	Bemerkung
1	S/ E-Schrank	Magnetron	MI-29	30 kV	
2	S/ E-Schrank	Thyratron	TGI 2-400/ 16	16 kV	
3	Sichtgerät	Kathodenstrahlröhre	31 LM 6	10 kV	in Sichtgerät

Die Werte der Ortsdosisleistung an Aufenthaltspunkten und die daraus abgeleiteten Werte der Ortsdosis sind in Abschnitt 3.2 angegeben.

2.3 Geschützrichtstation GRS-9

Die Geschützrichtstation GRS-9 diente in der Truppenluftabwehr der NVA zum Radarsteuerung von Flugabwehrkanonen. Die Geräte sowjetischer Bauart wurden ab 1957 in die NVA eingeführt und bis etwa 1975 in verschiedenen Versionen genutzt. Abb. 5 ist die Gesamtansicht des in einem Trailer untergebrachten Gerätes. Abb 5 zeigt ebenfalls die Anordnung der Geräte und Bedienerplätze in der Geschützrichtstation.

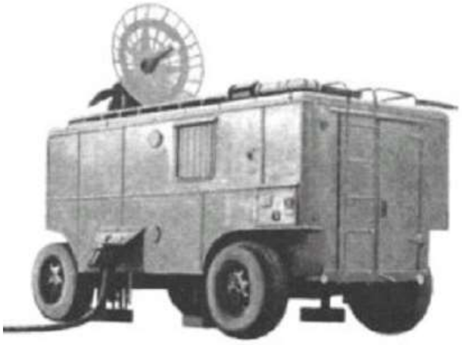

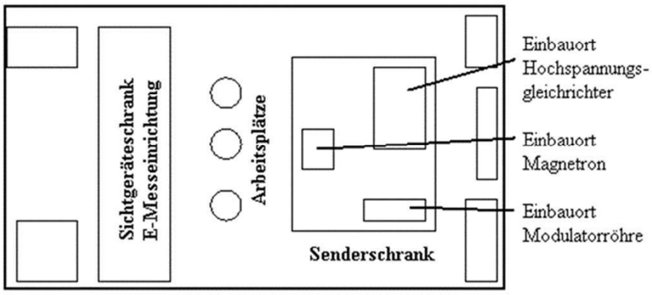

Abb. 5: Gesamtansicht der GRS-9 (links) und die Anordnung der Baugruppen und Bedienerplätze (Arbeitsplätze) in der mobilen Kabine

Die in der Sendeanlage der verschiedenen Versionen (GRS-9, GRS-9A, GRS-9 AM) eingebauten Röntgenstörstrahler sind in Tabelle 3 zusammengefasst.

Tabelle 3: Zusammenfassung der Störstrahler der verschiedenen Modifikationen der Geschützrichtstation GRS-9

**Modifikation**	**Modulator**	**HF- Generator (Magnetron)**	**Sperrdioden**	**23 kV-Gleichrichter**
GRS-9	3x GMI – 30	MI – 18 21	3 x W1-0,1/40	2x W1-0,1/40
GRS-9A	1x GMI – 90	MI – 30	---	2x W1-0,1/40
GRS-9AM	1x GMI – 90	MI – 30	1x WI 1 – 30/25	2x W1-0,1/40

Zu den Werten der Ortsdosisleistung an Aufenthaltspunkten und die daraus abgeleiteten Werte der Ortsdosis wird auf Abschnitt 3.3 verwiesen.

2.4 Höhenfinder PRW-11

Die Höhenmess-Radargeräte des Typs PRW-11 dienten zur Bestimmung der Höhe von Flugzielen in einer Höhe bis 30 km und in einer Entfernung bis 400 km in Richtungen, die durch eine Rundblickstation (RBS) ermittelt wurden. Entsprechend wurden die Höhenmesser im Verbund mit Rundblickstationen der Typen P-35, P-37, P-12 oder P-18 betrieben. Hauptsächlich wurden sie zu Aufgaben der Luftraumüberwachung und der Leitung von Jagdflugzeugen eingesetzt. Sie Geräte wurden ab Ende der 1960-er Jahre in die NVA eingeführt und bis 1991 in großen Stückzahlen betrieben.

Abb. 6 ist die Ansicht des in einer drehbaren Kabine untergebrachten Höhenmessers. Diese Sende/Empfangskabine war drehbar auf einer Lafette montiert und beinhaltete die Komponenten Modulator, Sender, Empfänger mit Störschutz und Antennensteuerung. Die Sichtgeräte mit den Bedienelementen der PRW-11 waren in einem separaten Hänger untergebracht.

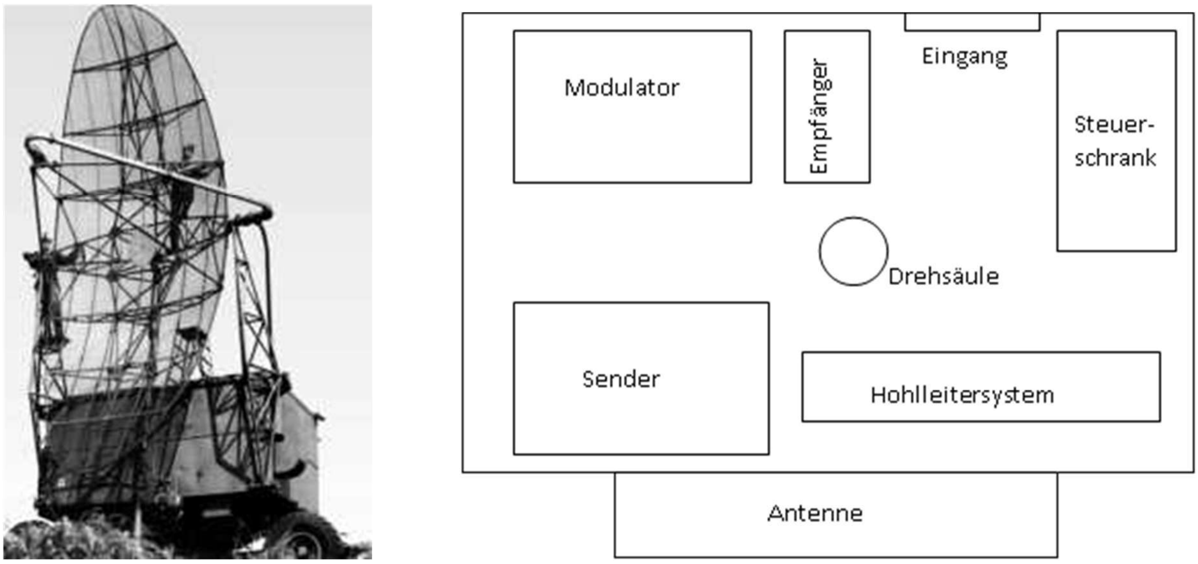

Abb. 6: Gesamtansicht der Sende/Empfangskabine der PRW-11 und der Anordnung der Baugruppen in der Kabine

Die im Modulator und im Sender der PRW-11 eingebauten Röntgenstörstrahler sind in Tabelle 4 angegeben.

Tabelle 4: Zusammenfassung der in der Sende/Empfangs-Kabine des PRW-11 eingebauten Röntgenstörstrahler

**Nr**	**Einbauort**	**Bauteil**	**Type**	**Betriebs-spannung**	**Bemerkung**
1	Modulator	Thyratron	TGI 1-700/25	24 kV	
2	Modulator	Thyratron	TGI 1-1000/25	24 kV	Ab etwa 1980 Ersatz für Type TGI 1-700/25
3	Sender	Magnetron	MI 125	50 kV	

2.5 Flugabwehrraketensystem S-75/S75-M

Die Flugabwehrraketensysteme (FlaRakSysteme) S-75 und S-75M waren im Bereich der NVA zur Bekämpfung von Luftzielen in mittleren und großen Höhen bestimmt. Die ersten Waffensysteme mit der russischen Bezeichnung S-75 'Dwina' wurden 1960 von der NVA übernommen und zum Objektschutz wichtiger administrativer und wirtschaftlicher Zentren eingesetzt. Ab dem Jahre 1963 wurden die Waffensysteme S-75 ‘Dwina‘ schrittweise durch die modernisierte Version S-75M ‘Wolchow' ersetzt und weitere neu aufgestellte

FlaRak-Verbände damit ausgerüstet. Abb. 7 zeigt die Kabine PA/PW des Systems S-75M im gefechtsbereitem Zustand. Zum FlaRakSystem S-75W gehörten noch weitere Kabinen für die Leittechnik mit dem Kommandosender für die Lenkflugkörper (Kabine AA/AW) sowie mit den Sichtgeräten für die Gefechtsführung (Kabine UW) sowie weitere Hänger für die Stromerzeugungsaggregate.

Um die Leitstation herum waren bis zu 6 Startrampen für je einen Lenkflugkörper aufgestellt.

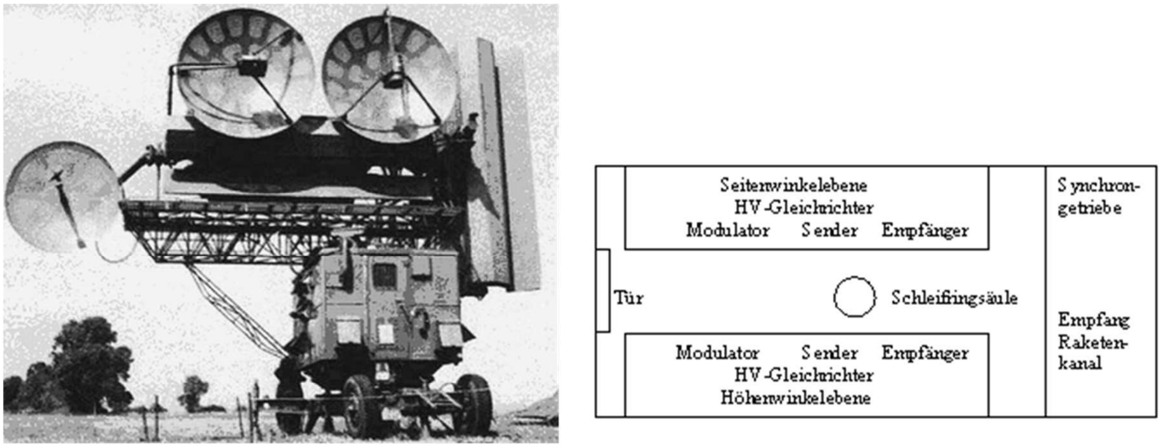

Abb. 7: Gesamtansicht der Kabine PW des FlaRakSystems S75M mit den Antennen für die Zielverfolgung und die Steuerung der Flugkörper (links) und das Lageschema der Baugruppen in der Kabine (rechts), die gleichzeitig Antennenträger ist.

Die Baugruppen mit Röntgenstörstrahlern waren in den Kabinen AW und PW. In Tabelle 5 sind die Angaben zu den in FlakRakSystem S-75W verwendeten Röntgenstörstrahler zusammengefasst. Die folgenden Abb. 8 und 9 zeigen Ansichten des Inneren der Kabine AW/PW mit geschlossenen Geräteschränken. Abb. 9 zeigt die geöffnete Modulatorbaugruppe und den geöffneten Geräteschrank eines Magnetronsenders.

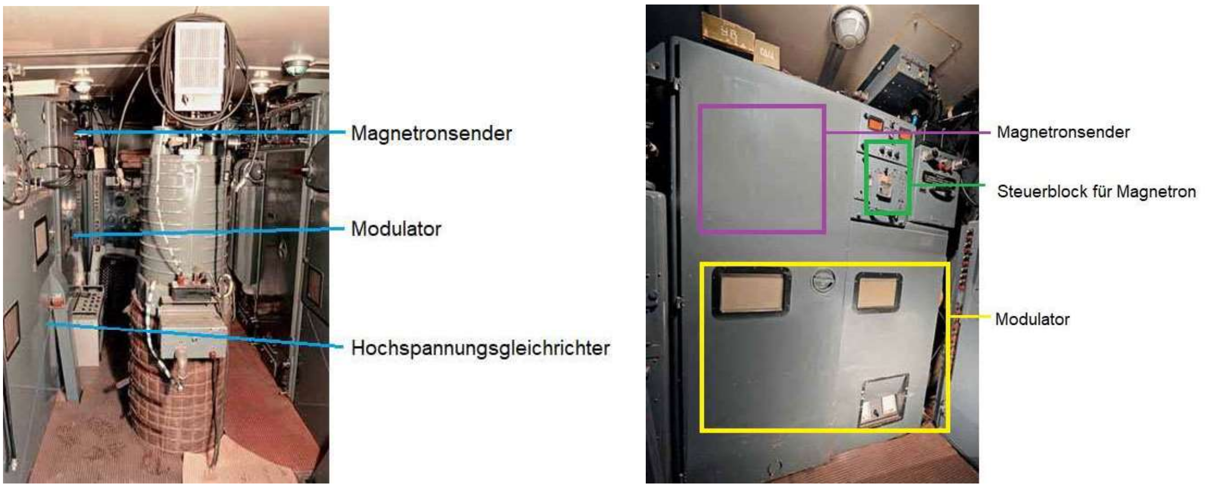

Abb. 8: Blick in die Kabine PW mit Kennzeichnung der Hauptbaugruppen eines Senders (links) und der Ansicht eines Geräteschrankes mit Modulator und Magnetron-sender)

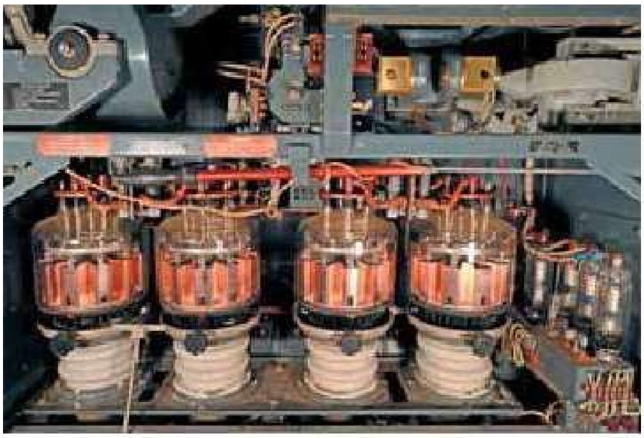

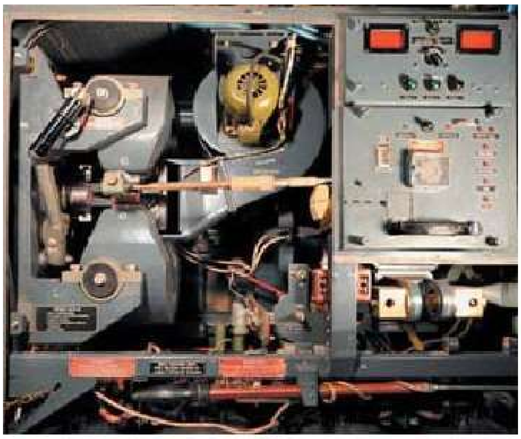

Abb. 9: Modulator mit geöffneten Geräteschrank mit den Schaltröhren GMI-2B (links) und Magnetronsender (rechts)

Tabelle 5: Röntgenstörstrahler im FlaRak- System S-75 (Anmerkung: Auf die Aufzählung von Bildröhren im Hänger mit dem Gefechtsstand (Kabine UW) wird verzichtet.

Nr	Kabine und Baugruppe	Bauteil	Typ	Anzahl	max. Betriebs-spannung
1	PA/PW Modulator	Schaltröhre	GMI-2B	2 x 4	22 kV
2	PA Sender 1	Magnetron	MI-143	1	40 kV
3	PA Sender 2	Magnetron	MI-144	1	40 kV
4	PW Sender 1	Magnetron	MI-147	1	40 kV
5	PW Sender 2	Magnetron	MI-148	1	40 kV
6	PA/PW Kompensationsstufe	Schaltröhre	GMI-90	2 x 2	22 kV
7	AA/AW Modulator	Schaltröhre	GMI-90	2	15 kV
8	AA/AW Sender	Scheibentriode	o.A.	1	20 kV

3 Werte der gerätespezifischen monatlichen Körperdosen

Zu den an Arbeits- und Aufenthaltspunkten kann bei den hier zu betrachtenden Radargeräten der ehemaligen NVA auf Messungen der Röntgenstörstrahlung zurückgegriffen werden, die noch während der Nutzungszeit dieser Geräte bei der Bundeswehr durchgeführt wurden oder auf Messungen, die an dazu wieder in Betrieb genommenen Geräten in Dienststellen/Einheiten der Bundeswehr durchgeführt wurden. Diese Ergebnisse sind in Messberichten der Strahlenmessstellen Nord und Süd der Bundeswehr dokumentiert und in den für die Bearbeitung von WBD-Anträgen erstellten gerätespezifischen internen[2] Ergebnisberichten zusammengefasst.

Aus den Werten der in den zu den folgenden Radargeräten angegebenen Werten der Photonen-Äquivalentdosisleistung ($\mathrm{d}H_x/\mathrm{d}t$)[3] werden zunächst die entsprechenden Werte der für Dosisbewertungen etablierten Umgebungsäquivalentdosis $H^*(10)$ berechnet. Die Umrechnung ergibt sich aus der Energie der Photonen. Hier wird in vereinfachender Weise der Umrechnungsfaktor für die Maximalenergie der Photonen der Röntgenstörstrahlung, die sich aus der Betriebsspannung des Röntgenstörstrahlers ergibt, eingesetzt.

Aus diesen Werten der Ortsdosis H_0 ($H^*(10)$ oder $H'(0,07)$) an beschäftigungsbedingten Aufenthaltsstellen des Körpers oder von Körperteilen Beschäftigter werden nach Maßgabe von Konversionsfaktoren f_k und Korrektionsfaktoren k_k gemäß

(1) $$H_T = f_k \, k_k \, H_0 \ .$$

Werte der Teilkörper- oder Organdosis H_T ermittelt. Die organ-/teilkörperspezifischen Konversionsfaktoren f_k tragen Streu- und Abschwächungseffekten durch das Gewebe und durch andere Körperteile Rechnung. Sie hängen von der Energie der Photonen und der Bestrahlungsgeometrie ab und sie sind für die Bestrahlung mit paralleler Photonenstrahlung angegeben. Abweichungen von dieser Idealisierung können durch den weiteren Korrektionsfaktor k_k Rechnung getragen. Im Anhang ist diese Berechnung, die auf einer Empfehlung der Strahlenschutzkommission [2,3] beruht, für die Arbeitsplätze an den untersuchten Radargeräten jeweils einzeln durchgeführt.

Aus den Teilkörper- oder Organdosiswerten H_T wird ergänzend jeweils die Effektive Dosis E gemäß:

(2) $$E = \sum_T w_T \, H_T$$

mit den normativen Wichtungsfaktoren w_T. Aus den nach (2) gewichtet ermittelten Teilkörperdosen errechneten Wert für E und dem im Regelwerk [2,3] für (homogene Bestrahlung) angegebenen Konversionsfaktor f_{k_E} lassen sich Werte der Ortsdosis angeben, die die gleiche

---

[2] Mit dem Hinweis ‚intern' ist nicht eine besondere Einstufung dieser Berichte –etwa als Verschlusssache – gemeint, sondern es wird auf den Charakter der Berichte als eine Arbeitsgrundlage der Versorgungsverwaltung hingewiesen. Auf eine durchgehende Zitierung der Grundlagen, wie für wissenschaftliche Reportierungen notwendig, wurde dort verzichtet.

[3] Zum Zeitpunkt dieser und der Untersuchungen der in der Bundeswehr noch genutzten RBS P-37 war die Dosisgröße H_x die etablierte Dosisgröße im Strahlenschutz. Mit der Kenntnis der maximalen Photonenenergie (gegeben durch die Betriebsspannung der Röntgenstörstrahler) können Werte H_x in die heute verwendete Dosisgröße Umgebungs-Äquivalentdosis (Formelzeichen $H^*(10)$) umgerechnet werden. Das wird im Weiteren vollzogen.

Effektive Dosis erzielt hätten. Für die überwiegend angetroffene inhomogene Bestrahlung ergeben sich hier freilich Diskrepanzen.

3.1 Rundblickstationen P-15 und P-19

Die Werte der Ortdosisleistung (in der Dosisgröße Photonen-Äquivalentdosis H_x) wurden an einer dazu wieder in Betrieb genommenen RBS P-15 Die daraus abgeleiteten Werte an den Arbeits- und Aufenthaltsplätzen sind in Tabelle 6 angegeben.

Aus den für Arbeiten eingenommenen Aufpunkten/Aufenthaltsorten in der Kabine der RBS (Abb. 2) und den Aufenthaltszeiten ergeben sich Werte für die monatliche Ortsdosis. Diese Werte sind in Tabelle 7 zusammen mit dem Bezug auf Körperteile/Teilkörperregionen und Organe, die sich dort befunden haben, angegeben.

Wegen der gleichen Bauweise von Sender und Modulator der P-19 sind die an der P-15 erhobenen Ausgangswerte auf diese RBS übertragbar. Bei der Abstandsextrapolation der Ortsdosisleistung auf andere Aufenthaltsorte im Kofferaufbau ergeben sich aber andere Ausgangswerte. Die Werte für die P-19 sind in Tabelle 8 angegeben.

Tabelle 6: Maximale Werte der Ortsdosisleistung (ODL) am Sender- und Modulatorschrank der RBS P-15 mit der Angabe des Abstandes zu den Röntgenstörstrahlern (RS), der ggf. exponierten Körperbereiche und der Orientierung des Strahlenfeldes (Hochspannung 27 kV). Für die Orientierung von Strahlenbündeln sind die Bezeichnungen 'ap' = anterior-posterior (von vorn) und 'pa' = posterior-anterior (von hinten) üblich.

**Zeile Nr.**	**Arbeitsbereich/ggf. exponierte Körperbereiche / (Orientierung des Strahlenfeldes)**	**Pos. Nr.** (siehe Abb.2)	**Zustand der Türen**	**Abstand**	**max. ODL**	**Bem. Nr.**
1	Sender und Modulator/ Hände und Beine (ap)	1	offen	30 cm zu RS	2,1 mSv/h	
2	Sender und Modulator/ Kopf, Körperstamm (ap)	1	offen	50 cm zu RS	0,89 mSv/h	
3	Sender (Wanderfeldröhre)/ Kopf, Körperstamm (ap)	1	geschlossen, Türspalt offen	50 cm zu RS	0,108 mSv/h	1)
4	Sender und Modulator/ Hände	1	geschlossen, Serviceklappe offen.	2 cm zu Frontplatte 25 cm RS	0,50 mSv/h	2)
5	vor Sender und Modulator zur Überwachung des Funkorters (siehe Abschnitt 4.3) / Kopf (pa)	1	geschlossen, Türspalt offen	30 cm zu RS	0,30 mSv/h	1),3)
6	dito/ Becken (pa)	1	geschlossen, Serviceklappe offen	25 cm zu RS	0,50 mSv/h	4)
7	vor Empfänger Körperstamm im Beckenbereich (pa)	2	geschlossen, vor Bleiglas-fenster	10 cm zur Oberfläche 20 cm RS	0,0036 mSv/h	
8	Sichtgerät/ Kopf, Körperstamm (pa)	4	geschlossen	60 cm zu RS	0,00028 mSv/h	

1) ODL Beiträge entstanden durch ein schmales Strahlenbündel, das wegen verbogener und nicht vollkommen dicht schließender Türen nach oben austrat. Das Strahlenbündel war in 10 cm Abstand knapp 5 cm breit.
2) Strahlenbündel erfasste nur die Hände
3) Exposition entstand für den Fall, dass sich der Techniker weit nach hinten lehnte und sein Kopf- und Halsbereich durch ein schmales Strahlenbündel erreicht werden konnten, das bei nicht vollkommen schließender Tür (siehe Abbildung 9) nach oben austrat.
4) Aus der Serviceklappe trat ein schmales Strahlenbündel flach zur Gerätefront aus und konnte den Beckenbereich des vor dem Schrank sitzenden Technikers bei weit zurückgelehnter Sitzposition erreichen. Dem Zustand der Serviceklappe (offen - geschlossen) wurde nach Auskunft von Zeitzeugen keine Bedeutung beigemessen.

Tabelle 7: Auf Körperbereiche und Expositionsdauer bezogene zusammengefasste maximale monatliche Ortsdosis in der Dosisgröße H_x (Photonen-Äquivalentdosis) für den an der RBS P-15 tätigen Personenkreis.

**Zeile Nr.**	**Personenkreis**	**Körperbereich**	**hauptsächliche Orientierung des Strahlenbündels**	**maximale monatliche Ortsdosis**	
				***H*x**	***H**(10)**
1	Stationsleiter/ Gruppenführer	Hände		2,1 mSv	2,216 mSv[1])
2		Beine	ap	2,1 mSv	1,638 mSv
3		Kopf	ap	1,1 mSv	0,858 mSv
4		Kopf	pa	3,0 mSv[2])	2,340 mSv
5		Körperstamm	ap	1,1 mSv	0,858 mSv
6		Körperstamm im Beckenbereich	pa	0,072 mSv	0,0562 mSv
7		Becken	pa	5,0 mSv[3])	3,900 mSv
8	Funkorter	Kopf	pa	0,045 mSv	0,0351 mSv
9		Körperstamm	pa	0,045 mSv	0,0351 mSv

[1]) Hier (abweichend von der Spaltenüberschrift) auf die Dosisgröße H'(0,07) umgerechnet
[2]) Dosisbeitrag entsteht durch ein schmales Strahlenbündel (Ausdehnung wenige cm)
[3]) Der überwiegende Anteil (5 mSv) entsteht durch ein schmales Strahlenbündel (Ausdehnung wenige cm)

Tabelle 8: Auf Körperbereiche und Expositionsdauer bezogene zusammengefasste maximale monatliche Ortsdosis für den an der RBS P-19 tätigen Personenkreis.

**Nr**	**Personenkreis**	**Körperbereich**	**hauptsächliche Orientierung des Strahlenbündels**	**maximale monatliche Ortsdosis**	
				***H*x**	***H**(10)**
1	Stationsleiter/ Funkmesstrupp-führer	Hände		2,1 mSv	2,216 mSv
2		Beine	ap	2,1 mSv	2,216 mSv
3		Kopf	ap	1,1 mSv	0,866 mSv
4		Körperstamm	ap	1,1 mSv	0,866 mSv
5	Funkorter	Körperstamm im Beckenbereich	rechte Seite	0,8 mSv	0,624 mSv
6	Kontroll-personal	Kopf	rechte Seite	0,3 mSv [1])	
7		Beckenbereich	rechte Seite	0,5 mSv [2])	

[1]) Dosisbeitrag entsteht durch ein schmales Strahlenbündel (Ausdehnung wenige cm).
[2]) Der überwiegende Anteil entsteht durch ein schmales Strahlenbündel (Ausdehnung wenige cm).

An beiden RBS ist bei der Dosisberechnung zu unterscheiden zwischen den für die Radartechnik verantwortlichen Stationsleiter einerseits und en daran tätigen Funkortern. Der Stationsleiter hat technische Arbeiten auch am gelegentlich auch kurz am geöffneten und in Betrieb befindlichen Modulator gearbeitet und es sind höherer Werte der Ortsdosisleistung für diese Zeitabschnitte anzusetzen als für die Funkorter. Die Funkorter saßen bei beiden RBS in unveränderter Orientierung zum Modulator-/Senderschrank und für sie ist (im Wesentlichen) nur eine Bestrahlungsgeometrie zutreffend. Für den Stationsleiter, der neben den Technischen Arbeiten mit Körperorientierung zum Modulator-/Senderschrank mit Bestrahlung ap – anterior-posterior) auch die Funkorter überwacht hat, ist dabei auch eine andere Bestrahlungsrichtung (pa-posterior-anterior) wie für die Funkorter vorgekommen. Für den Stationsleiter sind somit zwei Expositionsszenarien auszuwerten und zusammengefasst in Rechnung zu stellen.

Zu den Einzelheiten wird auf den Anhang 2 verwiesen, in Tabelle A1 sind die Werte für die in der Strahlenschutzverordnung (zur Berechnung der Effektiven Dosis nach ICRP 2007) herausgegriffene Organe zusammengestellt. Als Wert der pro Beschäftigungsmonat in Rechnung zu stellenden Effektiven Dosis ergibt sich:

P-15:	Stationsleiter:	0,315 mSv
	Funkorter (Operator):	0,031 mSv
P-19:	Stationsleiter (Techniker)	0,353 mSv
	Funkorter (Operator)	0,033 mSv

3.2 Rundblickstationen P-35 und P-37

Aus den in Abb. 4 dargestellten Aufpunkten ergeben sich nach Maßgabe der für die an geöffneten Sender/Empfänger-Schränken erhobenen Messwerte die in Tabelle 9 zusammengestellten Werte der Ortsdosisleistung. Die zu berücksichtigende Röntgenstörstrahlung stammt von den Thyratrons (Betriebsspannung 16 kV). In Tabelle 9 sind die aus der Auswertung von 9 Messberichten sich ergebenden Maximalwerte an Aufenthaltspunkten von Körperteilen angegeben. Zur Berechnung von Teilkörperdosen wird auf den Anhang verwiesen.

Die für gleiche Positionen in verschiedenen Messungen an verschiedenen Geräten ermittelten Dosiswerte weisen eine breite (über etwa zwei Größenordnungen reichende) Verteilung auf. Im Anhang 1 werden Hinweise zur Verteilung der Messwerte gegeben.

Die sich aus weitgreifenden Arbeitszeitansätzen errechneten maximalen Werte der monatlichen Dosiswerte sind ebenfalls in Tabelle 9 angegeben.

Tabelle 9: Maximale Werte der Ortsdosisleistung und monatlichen Ortsdosis der Röntgenstörstrahlung und betroffene Körperteile für Stationsleiter und Techniker der RBS P-37 (Spannung des Röntgenstörstrahlers 16 kV). Die verschiedenen Werte der ODL der Zeilen 3 – 5 ergeben sich aus unterschiedlichen Abständen des Beckens/Oberschenkels zum Sender/Empfänger-Schrank, gemäß der ermittelten Körperhaltung bei den entsprechenden Zeitansätzen.

**Zeile Nr.**	**exponierter Körperbereich/ Orientierung des Strahlenfeldes**	**Ortsdosis-leistung**	**Zeitansatz pro Monat**	**max. monatliche Ortsdosis**		
				**Einzelbeitrag**	**Summe**	
1	Hände, Beine /ap (Beine)	100 µSv/h	1 h	100 µSv	100 µSv	
2	Oberkörper, Kopf /ap	16 µSv/h	1 h	16 µSv	16 µSv	
3	Becken, Oberschenkel /pa	110 µSv/h	20 min	37 µSv		
4	Becken, Oberschenkel /pa	12,2 µSv/h	7 h	85,4 µSv		
5	Becken, Oberschenkel /pa	27,5 µSv/h	8 h	220 µSv		
6	Becken, Oberschenkel /pa				343 µSv	

Unter Berücksichtigung der Konversions- und Korrektionsfaktoren ergeben sich die für einzelne Organe und Teilkörperbereiche in Tabelle A4 angegebenen Werte der monatlich rezipierten Dosen. Als aus dem Maximalwert der Messwerte ermittelte Wert der Effektiven Dosis ergibt sich für einen an der P-37 tätigen Techniker 8,0 µSv.

Unter Berücksichtigung aller an RBS des Typs P-37 erhobenen Messwerte ergeben sich die in Tabelle 10 zusammengestellten Werte. Der Medianwert der monatlichen Ortsdosis bezogen auf den Wert 100 µSv für den Ort der Hände beträgt lediglich 10,5 µSv Messwerte beträgt, sodass die auch in Tabelle A1 angegebenen Werte der Teilkörperdosen um, den Faktor 10µSv/100µSv = 0,1 zu reduzieren sind.

Tabelle 10: Zusammenstellung der Werte zur statistischen Charakterisierung der Auswertung der Messergebnisse zur RBS P-37

	**Monatliche Referenzdosis H_x [µSv]**	**Effektive Dosis [µSv]**	
Maximalwert	100	8,02	
75%-Perzentil	29,3	2,34	
Median	10,5	0,84	
25%-Perzentil	1,84	0,148	
Minimum	0,76		
Mittelwert	23,5 ± 31,59		

Zusammengefasst ist für einen an der P-37 tätigen Techniker eine pro Arbeitsmonat rezipierte effektive Dosis von im Mittel 0,8 µSv (in einem Bereich (0,25 –Perzentil – 0,75 Perzentil) zwischen 0,15 – 2,34 µSv) anzurechnen. Aus der Geometrie des Strahlenfeldes, das hauptsächlich den Beckenbereich ap (Bestrahlung von vorn) betroffen hat, sind die höchsten monatlichen Organdosiswerte an Blase und Gonaden mit (Medianwerte) 2,19 µSv bzw. 5,5 µSv aufgetreten. Der Wert der Hautdosis von 10 µSv betrifft nur den Bereich der Hände und Unterarme.

3.3 Geschützrichtstation GRS-9

Für Untersuchungen der Emission von Röntgenstörstrahlung wurde eine Anlage des Typs GRS-9AM wieder in Betrieb genommen, die dabei gefundenen Werte sind als repräsentativ für alle GRS-9-Anklagen anzusehen. In Tabelle 11 sind die Messwerte der Ortsdosisleistung (Dosisgröße Photonenäquivalentdosis H_x), die an der wieder in Betrieb genommenen Senderbaugruppe einer GRS-9AM erhoben wurden, zusammengestellt. Die sich daraus nach Maßgabe der Abstände und der Aufenthaltszeit bei den Arbeiten ergebenden Werte der monatlichen Dosis sind in Tabelle 12 angegeben.

Tabelle 11: Zusammenfassung der Werte der ODL (Photonen-Äquivalentdosisleistung, Formelzeichen H_x pro Stunde) an der GRS-9AM (Alle Röntgenstörstrahler werden mit maximal 23 kV betrieben.)

**Störstrahler**	**Zustand des Gehäuses**	**Abstand oder Aufpunkt**	**max. Messwert ODL**	**Zeile Nr.**
Modulator (GMI-90 mit Sperr-diode WI 1-30/25)	Geräteklappe geöffnet	20 cm zur Tetrode	8,1 mSv/h [1])	1
	Geräteklappe geschlossen, Abdeckklappe geöffnet	an Sichtfenster	3,5 mSv/h [2])	2
	Geräteklappe und Abdeckklappe geschlossen	20 cm zur Gehäuseoberfläche	DL_0	4
Sender (Magnetron MI-30)	Geräteklappe geöffnet	< 10 cm zum Magnetron	4 µSv/h [3])	5
	geschlossen	10 cm zu Gehäuse-oberfläche	DL_0	6
Hochspannungs-gleichrichter W1-0,1/40	Geräteklappe geöffnet	ca 20 cm (in Ebene der Geräteklappe)	180 µSv/h [4])	7
	geschlossen		DL_0	8

[1]) ODL-Wert ist für die Arbeitsplatzbetrachtung ohne Belang. Der angegebene Messwert betrifft die Dosisgröße $H_p(10)$.
[2]) betroffen ist der Kopf bei Kontrollen vor der geöffneten Abdeckklappe. Es wird unterstellt, dass die Hände an Körperstamm bleiben oder die Abdeckklappe halten, jedenfalls nicht das Sichtfenster abdecken.
[3]) ODL herrscht nur in räumlich engbegrenzten Bereich, in dem sich keine Bauelemente befinden, die die (in etwa 50 cm Abstand dahinter befindlichen) Gleichrichterröhren abschirmen. Betroffen sind durch die Röntgenstörstrahlung Hände und Kopf.
[4]) ODL-Wert ist für die Arbeitsplatzbetrachtung ohne Belang

Tabelle 12: Maximale monatliche Ortsdosis für Arbeiten am Modulator der GRS-9 (Hochspannung 23 kV)

**Aufpunkt/ betroffenes Körperteil**	**max. ODL**	**max. monatliche Arbeitszeit**	**max. monatliche Ortsdosis H_x**	
an geöffneter Abdeckplatte/ Kopf	3,5 mSv/h	30 min	1,75 mSv	

In Tabelle A5 des Anhangs sind die Konversionsfaktoren und die sich daraus ergebenden Werte der monatlich rezipierten Organdosen zusammengefasst. Die Exposition betraf den Kopf bei kurzen Inspektionsarbeiten an einer geöffneten Geräteklappe.

Als Monatswert der Effektiven Dosis ergibt sich E = 0,0465 mSv, der höchste Wert einer Organdosis beträgt 0,683 mSv für die Schilddrüse. Die in Tabelle A5 angegeben hohen Werte der Teilkörperdosen Haut (1,04 mSv) und Knochenoberfläche (0,282 mSv) betreffen nur die Teile im Strahlenfeld der geöffneten Serviceklappe.

3.4 Höhenmessgerät PRW-11

Für die Radargeräte des Typs PRW-11 liegen zwei Vermessungsprotokolle hinsichtlich Röntgenstrahlung aus Störstrahlern vor. Bei diesen Messungen wurde keine Werte der Ortsdosisleistung über dem Wert des Untergrundes festgestellt, sodass ausgehend von dieser Basis eine Dosisbetrachtung für Probanden, die an diesem Gerät gearbeitet haben, entfallen kann.

Bei Radargeräten des Typs PRW-13, ebenfalls ein Höhenmesser, wurde ein fast baugleicher Modulator verwendet, bei dem die gleichen Thyratrontypen unter gleichen Betriebsbedingungen eingesetzt wurden. Zur Absicherung der Bewertung wurden in den für die Versorgungsverfahren vorgesehenen Bewertungen zusätzlich die Messergebnisse zum Radargerät PRW-13 herangezogen.

Für die Radargeräte PRW-13 liegen 12 Vermessungsprotokolle hinsichtlich Röntgenstrahlung aus Störstrahlern vor.

Die durchschnittliche maximale Aufenthaltsdauer am Modulatorschrank bei möglicherweise geöffneten Geräteklappen betrug 5 Stunden pro Monat. Die Röntgenstörstrahlung stammt vom Glasthyratron (Betriebsspannung 24 kV). In Tabelle 13 sind die Ausgangswerte der auf der breiteren Datenbasis beruhenden Dosisbetrachtung angegeben.

Tabelle 13: Maximalwerte der monatlichen Ortsdosen bezogen auf die Aufenthaltsdauer und den Ort ggf. exponierter Körperteile bei geöffneten Geräteklappen am Höhenmessradar PRW-11 (Betriebsspannung des Störstrahlers 24 kV)

**Nr**	**Abstand**	**Körperteil**	**Maximale Ortsdosisleistung**	**Maximale monatliche Ortsdosis**
1	In Ebene der Gerätefront	Hände	6 µSv/h	30 µSv
2	40 cm Abstand zur Gerätefront	Körperstamm	0,5 µSv/h	2,5 µSv

In Tabelle A6 des Anhangs sind die Werte der Organ- und Teilkörperdosiswerte wie sie sich aus dem Maximalwert der Messwerte ergeben, zusammengefasst.

Auf Grundlage der Maximalwerte ergibt sich für die pro Arbeitsmonat anzurechnende Effektive Dosis der Wert 0,68 µSv, der höchste Organdosiswert wird mit 1,6 µSv für die Gonaden (aufgrund des im Vergleich zu den anderen Organen hohen Konversionsfaktors) erreicht. Der ebenfalls (vergleichsweise) hohe Wert für die Hautdosis von 17 mSv betrifft die Hände und Unterarme.

Aus der Auswertung auch der übrigen Messwerte bei den Messbedingungen der Zeile Nr 1 der Tabelle 13 ergeben sich die in Tabelle 14 zusammengefassten statistischen Kenngrößen. Der Medianwert aller für eine Expositionsbetrachtung relevanten Messwerte beträgt 1,5 µSv/h, also ¼ des Maximalwertes, der die Grundlage der Expositionsberechnung ist. Die in Tabelle A6 angegebene Dosiswerte sind also um den Faktor 0,25 zu reduzieren.

Tabelle 14: Ergebnis der Auswertung der Dosisleistungsmessungen an der PRW-11 und der am Thyratron des mit einem im wesentlichen baugleichen Modulator ausgestatteten PRW-13

**Nr**	**Größe**	**Wert**	
1	Mittelwert	1,63 ± 2,08 µSv	
2	Minimum	0,10	
3	25%-Quantil	0,15	
4	Median	1,50	
5	75%-Quantil	1,75	
6	Maximum	6,00	

Damit sind an Arbeitsplätzen der PRW-11 pro Tätigkeitsmonat als Wert der Effektiven Dosis 0,17 µSv (Medianwert) in einem Bereich (25%-Perzentil – 75%-Perzentil) zwischen 0,017 µSv und 0,2 µSv anzusetzen. Für die monatlich rezipierten Organ/Teilkörperdosis Gonaden ergibt sich der Wert 0,4 µSv (im Bereich 0,04 µSv (25%-Perzentil) und 0,46 µSv (75%-Perzentil).

3.5 FlaRakSystem S-75M

Da zu den Sender- und Modulatorbaugruppen des FlaRakSystems S-75M keine Ergebnisse von Ortsdosismessungen vorliegen, werden Werte der Ortsdosis aus Vergleichen mit anderen Sender-/Modulatorbaugruppen abgeleitet, in denen typgleiche Röhren bei vergleichbaren Betriebswerten eingesetzt werden.

Für die Kabine AW/PW mit den beiden Radarsendern die Erfassung der Elevation und des Azimut-Winkels des Flugzieles ergeben sich für Hautbaugruppen Hochspannungsversorgung, Modulator und Magnetronsender folgende Ergebnisse:

(1) Hochspannungsversorgung: An den in dieser Baugruppe verwendeten Gleichrichterröhren können Ortsdosisleistungswerte im Bereich einiger Hundert µSv/h auftreten. Im Schrank des Hochspannungsgleichrichters befand sich noch einen mit je zwei Schaltröhren des Typs GMI-90 ausgerüstete Kompensationsstufe zum Umschalten des Modulators bei Frequenzwechsel des Magnetrons. Diese Vorgänge kamen selten vor, sodass ein Dosisbeitrag durch diese Kompensationsstufen nicht relevant ist.
Oberhalb des Schrankes befanden sich Teile des Hohlleitersystems mit Sende-/Empfangsumschaltern. Arbeiten bei eingeschaltetem Sender sind dort normalerweise nicht notwendig, sodass ein Aufenthalt eines Beschäftigten vor dem Hochspannungsgleichrichter somit nicht erforderlich ist.

Die Geräteschränke bestehen aus Stahlblech und bieten ausreichende Abschirmung, Klappen und Lüftungsgitter sind nicht vorhanden. Eine mögliche Schwachstelle der Abschirmung ist ein Spalt an der Oberseite der Tür, durch den ein räumlich eng begrenzte Strahlenbündel austreten kann, was zu einer Exposition bei Arbeiten und dem Aufenthalt vor dem jeweiligen Sender bewirken kann, aber ohne Auswirkung auf Arbeiten am anderen Sender ist.
(2) Modulator: Direkt an im Modulator verbauten Röhren können bei der Betriebsspannung von 22 kV Werte der Ortsdosisleistung von mehreren mSv/h entstehen. Oberhalb des Modulatorschrankes ist das Magnetron mit dem Steuerblock für die Frequenzeinstellung eingebaut, dort sind Arbeiten auch bei eingeschaltetem Sender/Modulator notwendig. Der Geräteschrank und die Türen bestehen aus Stahlblech und gewährleisten eine vollständige Abschirmung der Röntgenstörstrahlung. Eine mögliche Schwachstelle bilden aber auch hier wie am Hochspannungsgleichrichter und - wie am Modulator der Rundblickstation P-15 messtechnisch erfasst- Spalte an der Türoberkante.
(3) Magnetronsender: Das Magnetron wird mit einer Hochspannung von 40 kV betrieben und ist so eingebaut, dass die HF-Auskopplung zur linken Seitenwand des Senderschrankes weist. Das Auftreten von Röntgenstörstrahlung an der Gerätefront des Magnetronsenders kann somit ausgeschlossen werden. Eine Dosisbetrachtung für Arbeiten direkt an dieser Baugruppe entfällt.

Für die Kabine AA/AW mit dem Kommandosender für die Steuerung der Lenkflugkörper ergibt sich aus analogen Betrachtungen für die Exposition gegenüber Röntgenstörstrahlung bei Arbeiten das Folgende:
(1) Hochspannungsgleichrichter: Der Hochspannungsgleichrichter befindet sich in einem fest verschraubten Einschub aus Stahlblech. Ein relevanter Beitrag zur Exposition ist nicht zu erwarten.
(2) Modulator: Bei den im Modulator verbauten Hochleistungs-Schaltröhren des Typs GMI-90 sind bei einer Betriebsspannung von 15 kV Werte der Ortsdosisleistung bis 1 mSv/h direkt an der Röhre zu erwarten. Die Stahlblechtüren des Modulatorschrankes schirmen die weiche Röntgenstörstrahlung vollständig ab, sodass eine Exposition nur bei geöffnetem Geräteschrank oder durch Schlitze der Schranktür auftreten kann.
(3) Hochfrequenzgenerator: Die Senderöhre ist vollständig in einem Topfkreis aus Metall umschlossen. Das Auftreten von Röntgenstörstrahlung an der Gerätefront des HF-Generators kann ausgeschlossen werden.

Das Ergebnis dieser Betrachtung ist in Tabelle 15 zusammengefasst.

Tabelle 15: Zusammenfassung abgeschätzter Dosisanteile bei Arbeiten am FlaRakSystem S-75M.

Nr	Baugruppe	Tätigkeit	Ortsdosis-leistung	betroffene Körperteile	Exposi-tionszeit	Hoch-spannung	Monatl. Dosis
	**Kabine AW/PW**						
1	Hoch-spannungs-gleichrichter	Sichtkontrolle bei geöffneter Gerätetür,	0,1 mSv/h	Kopf, Oberkörper	1 h/Jahr	22 kV	
2		Kontrolle Hohlleiter-system	0,01 mSv/h	Kopf, Oberkörper	1 h/Monat	22 kV	
3		Summe					18,3 µSv
4	Modulator	Sichtkontrolle	5 mSv/h	Kopf, Oberkörper	1 h/Jahr	22 kV	
5		Arbeit am Steuerblock	0,2 mSv/h	Kopf, Oberkörper	2h /Monat	22 kV	
6		Summe					816,7 µSv
	**Kabine AA/AW**						
7	Modulator	Sichtkontrolle bei geöffneter Gerätetür	0,5 mSv/h	Kopf und Oberkörper	0,5 h/Jahr	15 kV	20,8 µSv
8		Arbeiten an Steuerblock	0,1 mSv/h	Obere und untere Extremitäten	1 h/Monat	15 kV	100 µSv

In Tabelle A7 des Anhanges sind die sich daraus ergebenden Werte der monatlich rezipierten Organ- und Teilkörperdosiswerte angegeben. Für die Effektive Dosis ergibt sich der Wert 86 µSv, wegen der Exposition der oberen und unteren Extremitäten und dem vergleichsweise hohen Konversionsfaktor wird die höchste Organdosis (abgesehen von der Hautdosis für Hände und Unterarme) für die Schilddrüse mit 330 µSv erreicht.

4 Zusammenfassung

Das Ergebnis der Dosisberechnung an den für den Anteil der Probanden, die bei der ehemaligen NVA gedient haben und für die eine tätigkeitsbedingte Exposition gegenüber Röntgenstörstrahlung vorgekommen ist, ist in Tabelle 16 angegeben.

Tabelle 16: Zusammenfassung der monatlichen Werte der effektiven Dosen bei Tätigkeiten an Radargeräten der ehemaligen NVA

**Nr**	**Radargerät**	**Tätigkeit**	**Monatliche Dosiswerte**		**Bemerkung**
			**Effektive Dosis**	**Organdosis Keimdrüsen**	
1	P-15	Techniker	0,318 mSv	0,346 mSv	Dosiswert stammt aus einer Messung eines wieder in Betrieb genommenen Gerätes
2		Funkorter	0,0032 mSv	0,2 µSv	
3	P-19	Stationsleiter	0,353 mSv	0,935 mSv	
4		Funkorter	0,0334 mSv	0,0117 mSv	
5	P 35, P-37	Techniker	0,84 µSv	5,49 µSv	Medianwert aus 9 Messberichten
6	GRS-9	Techniker	0,0465 mSv	-	Dosiswert stammt aus einer Messung eines wieder in Betrieb genommenen Gerätes
7	PRW-11	Techniker	0,17 µSv	0,40 µSv	Medianwert aus 7 Messwerten
8	S-75	Techniker	83,6 µSv	-	Dosiswert stammt aus der Abschätzung typischer Dosisleistungswerte an den Störstrahlern und der Analyse der technischen Gegebenheiten.

Schirmer Andreas
Digital unterschrieben von Schirmer Andreas
Datum: 2021.07.08 11:19:14 +02'00'

Dr. A. Schirmer

Anhang 1: Hinweise zur Statistik von Messwerten an den Funkmessstationen P-37 und PRW-11

Für die untersuchten Funkmessgeräte der ehemaligen NVA liegen nur zur RBS P-37 mehrere Messberichte vor. Für das Höhenmessgerät PRW-11 war die Datenbasis durch Erweiterung auf Messungen am weitgehend baugleichen Modulator des Höhenmessgerätes PRW-13 erweitert worden. Auch bei der begrenzten Datenbasis können Überlegungen zur Art der Verteilung der Messwerte geboten sein.

Zu den für diese beiden Radargeräte vorliegenden Messreihen wurden in den jeweiligen Abschnitten die statistischen Kenngrößen (in Tabelle 10 bzw. 14) angegeben. In Abb. 10 sind die Verteilungen der beiden Messwertreihen in normierten Dosisskalen als kumulierte Anteile als Funktion der monatlichen Dosis zusammengestellt.

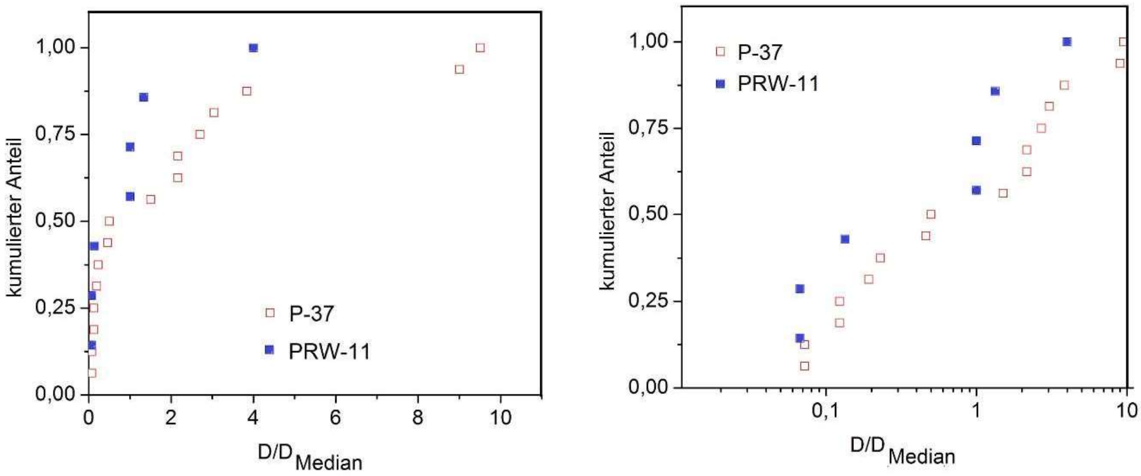

Abb. 10: Zusammenstellung der Messwerte für die Funkmessstationen P-37 und PRW-11 als kumulierte Anteile der Dosiswerte bezogen auf den median der jeweiligen Messreihe in linearer (links) und logarithmischer (rechts) Dosisskala.

Auf eine eingehendere rechnerische Analyse wir zunächst verzichtet. Aus der Wahl der Dosisskalen –hier linear und logarithmisch- ergibt sich, dass die Annahme einer logarithmischen Normalverteilung die Daten besser erfasst als eine einfache Normalverteilung.

Im Ergebnis folgen z.T. erhebliche Reduzierungen der anzusetzenden Dosiswerte, wenn etwa Medianwerte anstelle der Maximumwerte für die Dosisabschätzung zu Grund gelegt werden.

Anhang 2: Hinweise zur Berechnung von Teilkörperdosiswerten und der Effektiven Dosis

Die Berechnung der Teilkörper- oder Organdosiswerte erfolgt anhand des einschlägigen Regelwerkes. Ausgangsgröße ist die während der Zeit, in der die Messungen durchgeführt wurden, gebräuchliche Photonen-Äquivalentdosis (H_x) , die mittels der Spannung der Röntgen-störstrahler in die für die Bewertung zu verwendende Umgebungs-Äquivalentdosis H*(10) (oder – bei Bewertung der Hautdosis- in die Richtungs-Äquivalentdosis H‘(0,07)) anhand des in [2] angegeben Diagrammes (Abb. 3.1) umgerechnet wird. Mit der Bezeichnung H_0 für die Ortsdosis sind das die in den folgenden Tabellen angegeben Werte der monatlichen Ortsdosis an den Positionen der Teilkörperbereiche oder Organe.

In den weiteren Spalten der folgenden Tabellen werden die Bestrahlungsgeometrie und die durch die Photonenenergie bestimmten Konversionsfaktoren (ermittelt durch Ablesung der Diagramme in [2,3] angegeben. Die Konversionsfaktoren beruhen auf Berechnungen von aufrecht stehenden Körperphantomen, die durch parallele Strahlung exponiert werden. Einem kurzen Abstand des Körpers zur (punktförmigen) Quelle, also der Exposition durch divergente Strahlung wird durch sogenannte Korrektionsfaktoren k_k Rechnung getragen. Die in [2,3] angegeben Faktoren k_k werden hier übernommen. Weiterhin ist festzustellen, dass bei den untersuchten Arbeiten die Bestrahlung des aufrecht stehenden Menschen von vorn (ap-anterior-posterior) eine weitere Idealisierung darstellt. In [4] wurden Konversionsfaktoren für gebeugte Körperphantome bei Bestrahlung von oben (CRA-cranial) berechnet und verglichen. Es zeigt sich, dass bei einer Bestrahlung von oben der Konversionsfaktor kleiner ist als bei einer gebeugten Körperposition, bei der die Bestrahlung von CRA abweicht.

Übertragen auf die Bestrahlungsgeometrie mit gebeugter Körperhaltung an Radargeräte-schränken sind damit durch die Annäherung an die CRA-Bestrahlung in der Regel niedrigere Konversionsfaktoren zu erwarten[4]. Bei niedrigen Photonenenergien sind die Effekte ausgeprägter.

Die weiteren Spalten der folgenden Tabellen enthalten die schließlich berechneten monatlichen Teilkörper- oder Organdosiswerte. Die an den Spaltenenden aufgeführten Angaben der Effektiven Dosis sind die nach Maßgabe der Wichtungen w_T (Spalte 2) ausgeführten Summen.

[4] So ist bei weicher Röntgenstrahlung bei Bestrahlung (CRA-von oben) für bestimmte Organe (z.B. Speicheldrüse, Schilddrüse) ein erheblicher Abschwächungseffekt durch den darüber befindlichen Schädel zu erwarten.

Berechnung von Teilkörper- und Organdosiswerten aus den ermittelten Werten der monatlichen Ortsdosen

Tabelle A1: Werte der monatlichen Teilkörper-/Organdosen für Stationsleiter der RBS P-15

Radargerät		P15												
Stationsleiter														
**Nr**	**Organ/Gewebe *T***	**Wich-tung w_T**	**Monatliche Ortsdosis H_0 [mSv]**	**Geo-metrie**	**Kon-versions-faktor f_k**	**Kor-rektion k_k**	**Organ-/Teilkörper-dosis [mSv]**		**Monatliche Ortsdosis [mSv]**	**Geo-metrie**	**f_k**	**k_k**	**Organ-/Teilkörper-dosis [mSv]**	**Summe Be-strahlungs-geometrien [mSv]**
	1	2	3	4	5	6	7		8	9	10	11	12	13
1	Knochenmark, rot	0,12	0,8580	ap	0,195	1,10	0,1840		0,562	pa	0,1950	1,10	0,1205	0,3046
2	Dickdarm	0,12	0,8580	ap	0,360	1,30	0,4015		0,562	pa	0,0510	1,30	0,0373	0,4388
3	Lunge	0,12	0,8580	ap	0,250	1,10	0,2360		0,000	pa	0,1500	1,10	0,0000	0,2360
4	Magen	0,12	0,8580	ap	0,313	1,50	0,4022		0,000	pa	0,0375	1,50	0,0000	0,4022
5	Brust	0,12	0,8580	ap	0,350	1,50	0,4505		0,000	pa	0,0210	1,50	0,0000	0,4505
6	Keimdrüsen	0,08	0,0562	ap	0,720	1,50	0,0607		3,900	pa	0,0488	1,50	0,2855	0,3462
7	Blase	0,04	0,0562	ap	0,390	1,20	0,0263		3,900	pa	0,0250	1,20	0,1170	0,1433
8	Speiseröhre	0,04	0,8580	ap	0,188	1,00	0,1609		2,340	pa	0,0240	1,00	0,0562	0,2170
9	Leber	0,04	0,8580	ap	0,240	1,50	0,3089		0,000	pa	0,0750	1,50	0,0000	0,3089
10	Schilddrüse	0,04	0,8580	ap	0,756	0,90	0,5838		2,340	pa	0,0240	0,90	0,0505	0,6343
11	Haut	0,01	2,2155		0,600	0,90	1,1964		0,000	pa	0,5500	0,90	0,0000	1,1964
12	Knochenoberfläche	0,01	0,8580	ap	0,370	1,00	0,3175		0,562	pa	0,4000	1,00	0,2248	0,5423
13	Gehirn	0,01	0,8580	ap	0,026	0,70	0,0156		2,340	pa	0,0510	0,70	0,0835	0,0992
14	Speicheldrüsen	0,01	0,8580	ap	0,238	0,60	0,1223		2,340	pa	0,2439	0,60	0,3424	0,4647
15	andere	0,12												
				ap	Effektive Dosis		0,2655			pa	Effektive Dosis		0,0494	

Anmerkung: Aus der Angabe in [1] für den Konversionsfaktor für die Effektive Dosis E (nach ICRP 2007 [2]) zu $E/_{H^*(10)} = 0{,}34$ für die Bestrahlungsgeometrie ap und $E/_{H^*(10)} = 0{,}084$ für pa ergeben sich aus den hierfür die inhomogene Bestrahlung ermittelten Werten für E für eine dafür angesetzte räumlich homogene Ortsdosis $H^*(10)$ die Werte 0,78 mSv für ap bzw. $H^*(10)$ = 0,588 mSv für pa.

Tabelle A2: Werte der monatlichen Teilkörper-/Organdosen für Funkorter der RBS P-15

Radargerät		P-15						
Funkorter								
**Nr**	**Organ/Gewebe T**	**Wich-tung w_T**	**Monatliche Ortsdosis H_0 [mSv]**	**Geo-metrie**	**Kon-versions-faktor f_k**	**Kor-rektion k_k**	**Organ-/Teilkörper-dosis [mSv]**	
	1	2	3	4	5	6	7	
1	Knochenmark, rot	0,12	0,0351	pa	0,1950	1,10	0,0075	
2	Dickdarm	0,12	0,0351	pa	0,0510	1,30	0,0023	
3	Lunge	0,12	0,0351	pa	0,1500	1,10	0,0058	
4	Magen	0,12	0,0351	pa	0,0375	1,50	0,0020	
5	Brust	0,12	0,0351	pa	0,0210	1,50	0,0011	
6	Keimdrüsen	0,08	0,0351	pa	0,0488	1,50	0,0026	
7	Blase	0,04	0,0351	pa	0,0250	1,20	0,0011	
8	Speiseröhre	0,04	0,0351	pa	0,0240	1,00	0,0008	
9	Leber	0,04	0,0351	pa	0,0750	1,50	0,0039	
10	Schilddrüse	0,04	0,0351	pa	0,0240	0,90	0,0008	
11	Haut	0,01	0,0475		0,5500	0,90	0,0235	
12	Knochenoberfläche	0,01	0,0351	pa	0,4000	1,00	0,0140	
13	Gehirn	0,01	0,0351	pa	0,0510	0,70	0,0013	
14	Speicheldrüsen	0,01	0,0351	pa	0,2439	0,60	0,0051	
15	andere	0,12	0,0351					
				ap	Effektive Dosis		0,0032	

Anmerkung: Aus den Werten für den Konversionsfaktor für die Effektive Dosis E (nach ICRP 2007 [2]) zu $\frac{E}{H^*(10)}$ $f_{k\_E} = 0{,}084$ für pa ergibt sich aus dem für die inhomogene Bestrahlung ermittelte Wert für E für eine dafür anzusetzende räumlich homogene Ortsdosis $H^*(10) = \frac{E}{f_{k\_E}} = \frac{0{,}032\ mSv}{0{,}084} = 0{,}38\ mSv$.

<u>Tabelle A3:</u> Werte der monatlichen Teilkörper-/Organdosen für

Radargerät		P-19												
Stationsleiter									Funkorter					
**Nr**	**Organ/Gewebe** ***T***	**Wich-tung** w_T	**Monatliche Ortsdosis** H_0 **[mSv]**	**Geo-metrie**	**Kon-versions-faktor** f_{k_AP}	**Kor-rektion** k_k	**Organ-/Teilkörper-dosis [mSv]**		**Monatliche Ortsdosis** H_0 **[mSv]**	**Geo-metrie**	**Kon-versions-faktor** $f_{k\_LAT\_R}$	**Organ-/Teilkörper-dosis [mSv]**		
	1	2	3	4	5	8	7							
1	Knochenmark, rot	0,12	0,8658	ap	0,195	1,10	0,1857		0,6240	LAT_R	0,1000	0,0686		
2	Dickdarm	0,12	0,8658	ap	0,360	1,30	0,4052		0,6240	LAT_R	0,1260	0,1022		
3	Lunge	0,12	0,8658	ap	0,250	1,10	0,2381		0,6240	LAT_R	0,0500	0,0343		
4	Magen	0,12	0,8658	ap	0,313	1,50	0,4058		0,6240	LAT_R	0,0000	0,0000		
5	Brust	0,12	0,8658	ap	0,350	1,50	0,4545		0,6240	LAT_R	0,0263	0,0246		
6	Keimdrüsen	0,08	0,8658	ap	0,720	1,50	0,9351		0,6240	LAT_R	0,0125	0,0117		
7	Blase	0,04	0,8658	ap	0,390	1,20	0,4052		0,6240	LAT_R	0,0075	0,0056		
8	Speiseröhre	0,04	0,8658	ap	0,188	1,00	0,1623		0,0000	LAT_R	0,0250	0,0000		
9	Leber	0,04	0,8658	ap	0,240	1,50	0,3117		0,6240	LAT_R	0,0750	0,0702		
10	Schilddrüse	0,04	0,8658	ap	0,756	0,90	0,5891		0,0000	LAT_R	0,1250	0,0000		
11	Haut	0,01	2,2155		0,600	0,90	1,1964		0,0475	LAT_R	0,4000	0,0171		
12	Knochenoberfläche	0,01	0,8658	ap	0,370	1,00	0,3203		0,6240	LAT_R	0,2630	0,1641		
13	Gehirn	0,01	0,8658	ap	0,026	0,70	0,0158		0,0000	LAT_R	0,0750	0,0000		
14	Speicheldrüsen	0,01	0,8658	ap	0,238	0,60	0,1234		0,0000	LAT_R	0,4000	0,0000		
15	andere	0,12					0,0000							
					Effektive Dosis		0,3528			Effektive Dosis		0,0334		

<u>Anmerkung:</u> Aus den Konversionsfaktoren $f_{k\_E} = 0{,}3428$ für die Bestrahlungsrichtung ap und $f_{k\_E} = 0{,}084$ für pa können den aus den inhomogenen Bestrahlungsgeometrien ermittelten Werten der Effektiven Dosis als Dosiswerte einer homogenen Bestrahlung zugeordnet werden: $H^*(10)$ = 1,03 mSv (ap) – Exposition des Stationsleiters, und $H^*(10)$ = 0,398 mSv für den Funkorter.

Tabelle A4: Werte der monatlichen Teilkörper-/Organdosen für den Stationsleiter/Techniker der RBS P-37 ermittelt aus dem Maximalwert der Messwerte der Ortsdosisleistung

Radargerät		P-37						
Stationsleiter							MAX-Wert	
**Nr**	**Organ/Gewebe** ***T***	**Wich-tung** w_T	**Monatliche Ortsdosis** H_0 **[µSv]**	**Geo-metrie**	**Kon-versions-faktor** f_{k_AP}	**Kor-rektion** k_k	**Organ-/Teilkörper-dosis [µSv]**	
	1	2	3	4	5	8	7	
1	Knochenmark, rot	0,12	121,94	ap	0,024	1,10	3,3	
2	Dickdarm	0,12	121,94	ap	0,051	1,30	8,1	
3	Lunge	0,12	5,69	ap	0,020	1,10	0,1	
4	Magen	0,12	5,69	ap	0,025	1,50	0,2	
5	Brust	0,12	0,86	ap	0,833	1,50	1,1	
6	Keimdrüsen	0,08	121,94	ap	0,300	1,50	54,9	
7	Blase	0,04	121,94	ap	0,150	1,20	21,9	
8	Speiseröhre	0,04	5,69	ap	0,025	1,00	0,1	
9	Leber	0,04	5,69	ap	0,020	1,50	0,2	
10	Schilddrüse	0,04	5,69	ap	0,366	0,90	1,9	
11	Haut	0,01	88,89		1,260	0,90	100,8	
12	Knochenoberfläche	0,01	121,94	ap	0,100	1,00	12,2	
13	Gehirn	0,01	5,69	ap	0,000	0,70	0,0	
14	Speicheldrüsen	0,01	5,69	ap	0,038	0,60	0,1	
15	andere	0,12			0,048		0,0	
					Effektive Dosis		8,02	

Anmerkung: Aus dem Konversionsfaktor $f_{k\_E} = 0{,}17$ für die Effektive Dosis bei homogener Bestrahlungsrichtung ap ergibt sich aus dem hier für die inhomogene Bestrahlungsgeometrie ermittelten Werte der Effektiven Dosis E = 0,3528 µSv als Dosiswert einer äquivalenten homogenen Bestrahlung H*(10) = 47,2 µSv.

Tabelle A5: Werte der monatlichen Teilkörper-/Organdosen für den Techniker der Geschützrichtstation GRS-9RBS P-37 ermittelt aus dem Maximalwert der Messwerte der Ortsdosisleistung

Radargerät		GRS-9						
Stationsleiter							MAX-Wert	
**Nr**	**Organ/Gewebe** ***T***	**Wich-tung** w_T	**Monatliche Ortsdosis** H_0 **[mSv]**	**Geo-metrie**	**Kon-versions-faktor** f_{k_AP}	**Kor-rektion** k_k	**Organ-/Teilkörper-dosis [mSv]**	
	1	2	3	4	5	8	7	
1	Knochenmark, rot	0,12	0,000	ap	0,024	1,10	0,000	
2	Dickdarm	0,12	0,000	ap	0,051	1,30	0,000	
3	Lunge	0,12	0,000	ap	0,020	1,10	0,000	
4	Magen	0,12	0,000	ap	0,025	1,50	0,000	
5	Brust	0,12	0,000	ap	0,833	1,50	0,000	
6	Keimdrüsen	0,08	0,000	ap	0,300	1,50	0,000	
7	Blase	0,04	0,000	ap	0,150	1,20	0,000	
8	Speiseröhre	0,04	1,197	ap	0,025	1,00	0,118	
9	Leber	0,04	0,000	ap	0,020	1,50	0,000	
10	Schilddrüse	0,04	1,1967	ap	0,366	0,90	0,683	
11	Haut	0,01	1,661		1,260	0,90	1,046	
12	Knochenoberfläche	0,01	1,197	ap	0,100	1,00	0,282	
13	Gehirn	0,01	1,197	ap	0,000	0,70	0,021	
14	Speicheldrüsen	0,01	1,197	ap	0,038	0,60	0,097	
15	andere	0,12			0,048		0,000	
					Effektive Dosis		0,0465	

Anmerkung: Aus dem Konversionsfaktor $f_{k\_E} = 0{,}24$ für die Effektive Dosis bei homogener Bestrahlungsrichtung ap ergibt sich aus dem hier für die inhomogene Bestrahlungsgeometrie ermittelten Wert der Effektiven Dosis E = 0,0465 mSv als Dosiswert für eine äquivalente homogene Bestrahlung $H^*(10)$ = 0,193 mSv.

Tabelle A6: Werte der monatlichen Teilkörper-/Organdosen für den Techniker der Geschützrichtstation GRS-9RBS P-37 ermittelt aus dem Maximalwert der Messwerte der Ortsdosisleistung

Radargerät	PRW-11							
Stationsleiter							MAX-Wert	
Nr	Organ/Gewebe T	Wich-tung w_T	Monatliche Ortsdosis H_0 [µSv]	Geo-metrie	Kon-versions-faktor f_{k_AP}	Kor-rektion k_k	Organ-/Teilkörper-dosis [µSv]	
	1	2	3	4	5	8	7	
1	Knochenmark, rot	0,12	1,758	ap	0,146	1,10	0,282	
2	Dickdarm	0,12	1,758	ap	0,250	1,30	0,571	
3	Lunge	0,12	1,758	ap	0,138	1,10	0,266	
4	Magen	0,12	1,758	ap	0,200	1,50	0,527	
5	Brust	0,12	1,758	ap	0,250	1,50	0,659	
6	Keimdrüsen	0,08	1,758	ap	0,610	1,50	1,608	
7	Blase	0,04	1,758	ap	0,325	1,20	0,686	
8	Speiseröhre	0,04	1,758	ap	0,122	1,00	0,215	
9	Leber	0,04	1,758	ap	0,195	1,50	0,514	
10	Schilddrüse	0,04	1,758	ap	0,671	0,90	1,061	
11	Haut	0,01	29,490		0,650	0,90	17,252	
12	Knochenoberfläche	0,01	1,758	ap	0,290	1,00	0,509	
13	Gehirn	0,01	1,758	ap	0,038	0,70	0,046	
14	Speicheldrüsen	0,01	1,758	ap	0,161	0,60	0,169	
15	andere	0,12			0,143			
					Effektive Dosis		0,684	

Anmerkung: Aus dem Konversionsfaktor $f_{k\_E} = 0{,}26$ für die Effektive Dosis bei homogener Bestrahlungsrichtung ap ergibt sich aus dem hier für die inhomogene Bestrahlungsgeometrie ermittelten Wert der Effektiven Dosis E = 0,68 µSv als Dosiswert für eine äquivalente homogene Bestrahlung $H^*(10)$ = 2,61 µSv.

BAIUDBw

Strahlenmessstelle der Bundeswehr

Bericht-Nr.: S 209/20-E1

Retrospektive Dosisberechnung Röntgenstörstrahlung

Exposition gegenüber Röntgenstörstrahlung an Radar- und Feuerleitgeräten der Bundeswehr

28.07.2021

Munster

Verfasser: Dr. SCHIRMER, Andreas

Datum: 25.08.2021

Strahlenmessstelle der Bundeswehr
Bundesamt für Infrastruktur, Umweltschutz und Dienstleistungen der Bundeswehr

29633 Munster, den 25.08.2021
Humboldtstr. 1
Tel: 05192/ 136 -347, -538, FAX-506
BwFw: 90-2252-347, -538, FAX -506
StrahlenmessstelleBw@bundeswehr.org

Az 87 - 14 – 24

Bericht-Nr.: S 209/21-E1

Hinweise zur Körperdosis ehemaliger Beschäftigter durch Röntgenstörstrahlung an Radargeräten der Bundeswehr

1 Auftrag, Vorbemerkung

Auftragsgemäß legt die Strahlenmessstelle der Bundeswehr das Ergebnis der Dosisberechnung für Beschäftigte der Bundeswehr als Beitrag zur Studie des Instituts für Genomische Statistik und Bioinformatik, Universitätsklinikum Bonn, Rheinische Friedrich-Wilhelms-Universität Bonn vor. Dieser Bericht ist die Ergänzung des zu den Beschäftigten der ehemaligen NVA vorgelegten Ergebnisses [1]. Die dort gemachten Vorbemerkungen zur Ausgangssituation der Arbeitsplatzuntersuchungen und zur Arbeitsweise gelten auch für den Anteil der Arbeitsplätze der Bundeswehr, der im Übrigen damals zuerst untersucht wurde.

Für den hier vorgelegten Teil der Radargeräte der Bundeswehr sind für die Dosisbetrachtung der Probanden Arbeitsplätze an insgesamt 12 Radargeräten zu berücksichtigen, die in Tabelle 1 zusammengefasst sind.

Tabelle 1: Übersicht der betrachteten Radargeräte

Nr	Waffensystem	Expositionsrelevante(s) Radargerät(e)		
1	F-104 G (Starfighter)	F-15 NASARR		
2	Fla-Rak-System HAWK	HPIR		
3	Fla-Rak-System NIKE	LOPAR, HIPAR		
4	Feuerleitradar Deisswil-7	Deisswil-7		
5	Landekontrollgerät AN/MPN-11 mit Radar AN/CPN-4	AN/CPN-4		
6	Luftraumüberwachungsradar HADR	HADR		
7	Rundsuchradar, mobil AN/TPS-43	AN/TPS-43		
8	Rundsuchradar AN/FPS-7e	AN/FPS-7e		
9	Höhenmessradar AN/FPS-6	AN/FPS-6		
10	Höhenmessradar AN/FPS-89	AN/FPS-89		
11	Höhenmessradar S-244	S-244		

2 Kurzbeschreibung der Systeme und Expositionssituationen

2.1 Luftfahrzeugradar NASARR

Das Vorwärtssichtradar NASARR (North American Airborne Search and Ranging Radar, genaue Bezeichnung F 15 B-A/D) wurde in allen Versionen des in der Bundeswehr eingesetzten Luftfahrzeug-Musters F-104 G (Starfighter) eingebaut. Das NASARR war ein Allwetter-Zielsuch- und Entfernungsmessradar, es war als kompakte Baugruppe, bestehend aus Sender/Empfänger mit angebauter, schwenkbarer Parabolantenne unter dem Radom an der Lfz-Spitze eingebaut. Weitere Baugruppen wie Rechner, Kühlaggregate, Anzeige und Bedienelemente waren hinter dem Cockpit bzw. im Cockpit am Platz des Piloten eingebaut Abb. 1 zeigt die Anordnung der Baugruppen, Abb. 2 ist die Gesamtansicht des Radargerätes, das bei Wartungen und Reparaturen sowohl eingebaut im Lfz (bei abgebautem Radom) als auch ausgebaut auf Testständen betrieben wurde.

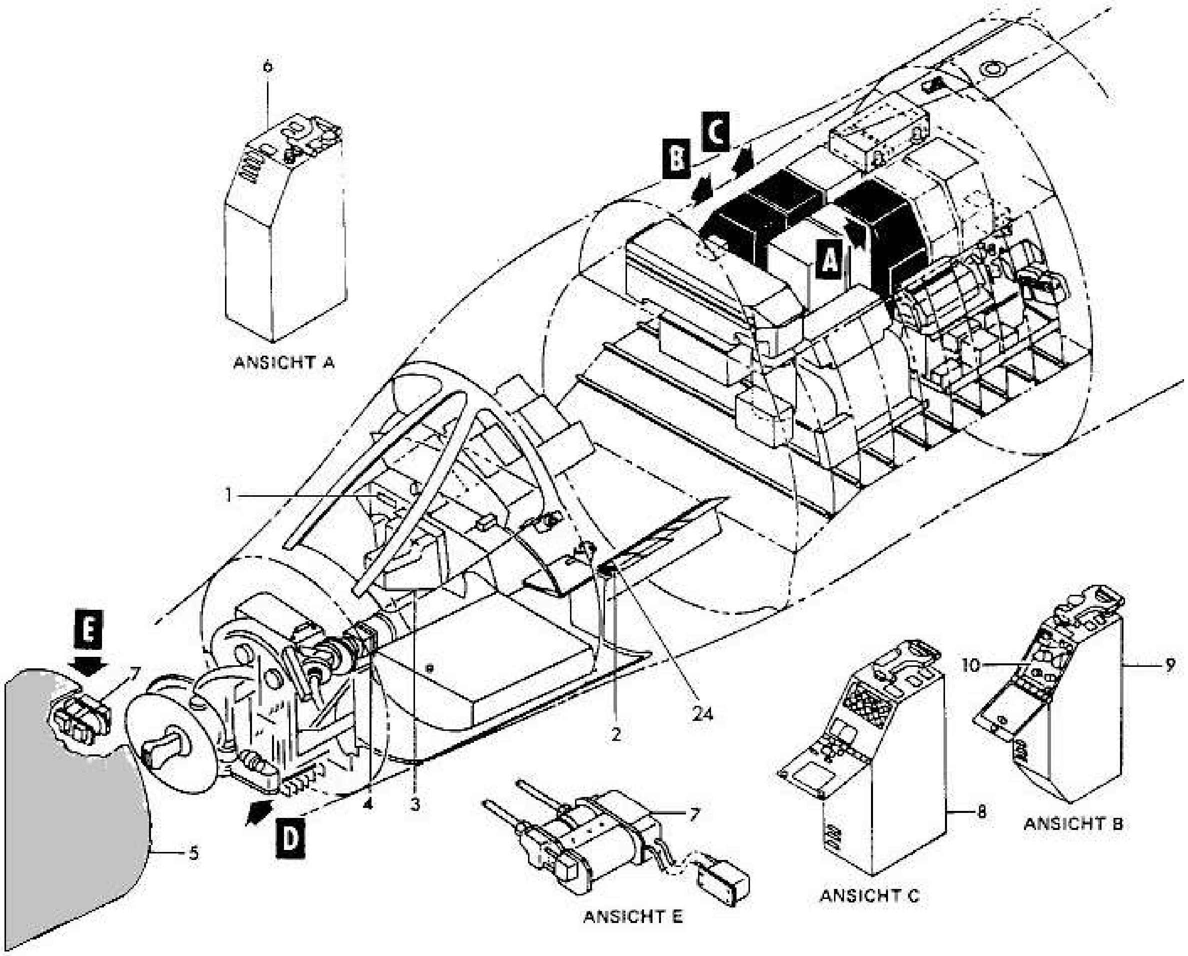

Abb. 1: Übersicht der Baugruppen des NASARR in Lfz F-104G (Starfighter). Der Radarsender mit den Störstrahlern ist die Baugruppe D. Zur Erklärung der einzelnen durchnummerierten Komponenten wird auf Abb. 2 verwiesen

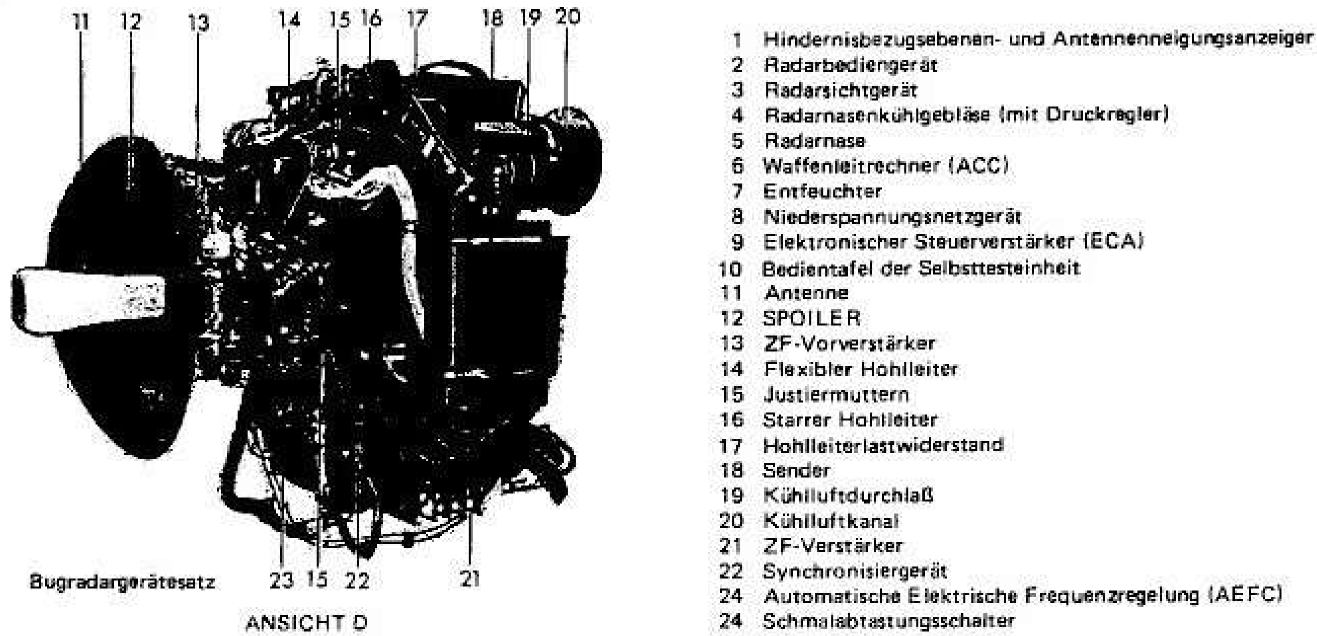

Abb. 2: Schematische Gesamtansicht der Sender/Antennenbaugruppe des NASARR mit Detailangabe der Bauteile. Das Magnetron befindet sich an der dem Betrachter abgewandten am Sender (Position Nr 18).

Zu den Arbeitsplatz- und Expositionsverhältnissen für Beschäftigte, die am NASARR gearbeitet haben, hat es mehrere Untersuchungen auch mit detaillierten Darstellungen und Dokumentationen der Arbeitsweisen an den Störstrahlern gegeben. Abbildung 3 enthält den wesentlichen Arbeitsschritt der Frequenzeinstellung an Magnetron.

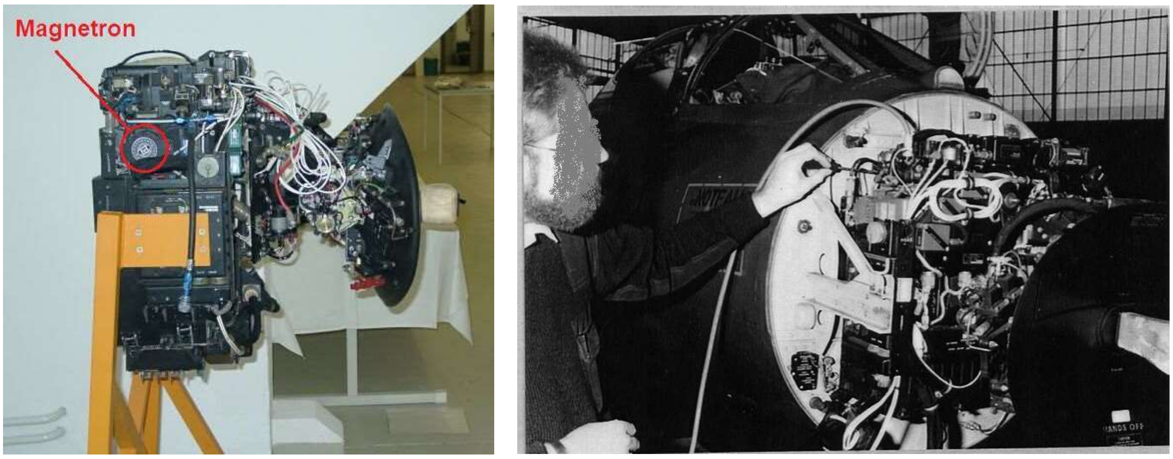

Abb. 3: Gesamtansicht des ausgebauten NASARR (links) und Darstellung technischer Arbeiten am Vollsysteme (NASARR im Lfz eingebaut): Einstellarbeit am Magnetron (rechts).

Die im NASARR betriebenen Röntgenstörstrahler sind in Tabelle 2 angegeben. Auf die besondere Betrachtung der im Anzeigegerät betriebenen Kathodenstrahlröhre (Betriebsspannung 12 kV) wird hier wie im Folgenden verzichtet[1].

[1] Die in Kathodenstrahlröhren von Radarsichtgeräten erzeugte Röntgenstörstrahlung wird durch das als großer Vakuumbehälter ausgelegte massive Glasgehäuse vollständig abgeschirmt.

Tabelle 2: Röntgenstörstrahler im Lfz-Radargerät NASARR (Es wurden in der Nutzungszeit des NASARR verschiedene Magnetron- bzw. Thyratron-Typen verschiedener Hersteller benutzt)

Nr	Störstrahler	Typen	Hoch-spannung	Einbauort
1	Magnetron	M4193, L4193A, YJ1210	25 kV	an Oberseite Sender
2	Thyratron	JAN 8613 (Keramikthyratron), F 5008B, KU 21, 6587	12 kV	im Inneren des Sendergehäuses

2.2 Radargeräte des Fla-Rak-Systems HAWK

Zur Bekämpfung von Luftzielen bis in Höhen von 15.000 m mittels bodengestützter Flugabwehrraketen wurde an 1963 das Waffensystem HAWK in die Bundeswehr eingeführt und von der Luftwaffe betrieben. Dem Auftrag entsprechend waren die insgesamt 9 Bataillone in festen Stellungen in einem von der Ostsee zu den Alpen reichenden Streifen aufgestellt. Alle Komponenten des Waffensystems waren verlegbar.

Das Waffensystem wurde im mehreren Programmen (ausgehend von BASIC-HAWK über I(improved) - HAWK zu letztlich PIP (Product Improvement Program)-HAWK verbessert, wobei allerdings die Technik der Radarsender nicht grundlegend betroffen war.
In einem HAWK-Verband wurden bis zu 5 verschiedene Radargerätetypen betrieben, von denen die vier Typen, die in den Kampfstaffeln betrieben wurden, in Tabelle 3 angegeben sind. Zusätzlich wurde auf der Bataillonsebene für die weiter reichende Luftraumaufklärung ein Rundsuchradargerät des Typs ASR-P betrieben.

Tabelle 3: Bezeichnung der Radargeräte der Versionen von HAWK

Radargerät	Bezeichnung im Verbesserungsprogramm		
	BASIC-HAWK	I-HAWK	PIP-HAWK
High Power Illumination Radar, HPIR	AN/MPQ-39	AN/MPQ-46	AN/MPQ-60
Pulse Acquisition Radar, PAR	AN/MPQ-35	AN/MPQ-50	AN/MPQ-50
Continuous Wave Acquisition Radar, CWAR	AN/MPQ-34	AN/MPQ-48	AN/MPQ-55
Range Only Radar, ROR	AN/MPQ-37	AN/MPQ-51	ausgesondert

Im Vorgriff auf die Darstellung der Messergebnisse wird hier darauf verwiesen, dass eine Dosisbetrachtung für die CWAR und ROR entfällt, an diesen Geräten wurden in den ab 1980 durchgeführten Dosismessungen keine Werte über dem messtechnischen Untergrund festgestellt. Die beiden Radargerätetypen, für die eine Dosisbetrachtung notwendig ist, sind in Abb. 4 dargestellt.

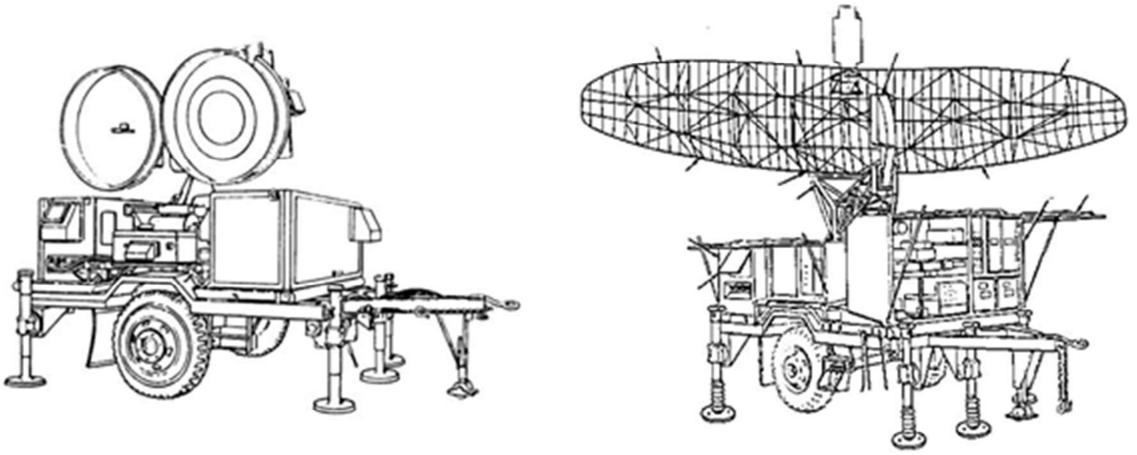

Abb. 4: Hochleistungsbeleuchtungsradar (HPIR - links) und Pulserfassungsradar (PAR – rechts) des Waffensystems HAWK in einsatzbereiter Konfiguration.

Die in den HPIR und PAR eingebauten Röntgenstörstrahler sind in Tabelle 4 angegeben.

Tabelle 4: Röntgenstörstrahler in den Radargeräten HPIR und PAR im Nutzungszeitraum von I-HAWK

Nr	Radargerät	Störstrahler	Typ	Betriebsspannung
	BASIC und I-HAWK			
1	HPIR	Klystron	VA 851 D	12 kV
2	PAR	Thyratron	JAN 5949 A	18 kV
3		Clipperdiode	JAN 6303	36 kV
4		Charging-/Shunt-Diode	JAN 6303	18 kV
	PIP HAWK			
	HPIR	Klystron	VA 868 E	12 kV
		Master Oszillator, Klystron	QKK 1229	11,3 kV
	PAR	Stabilotron	QK 630	36 kV
		Thyratron	JAN 5940 A	18 kV
		HV-Regulator	3 CW5000F1	14 kV

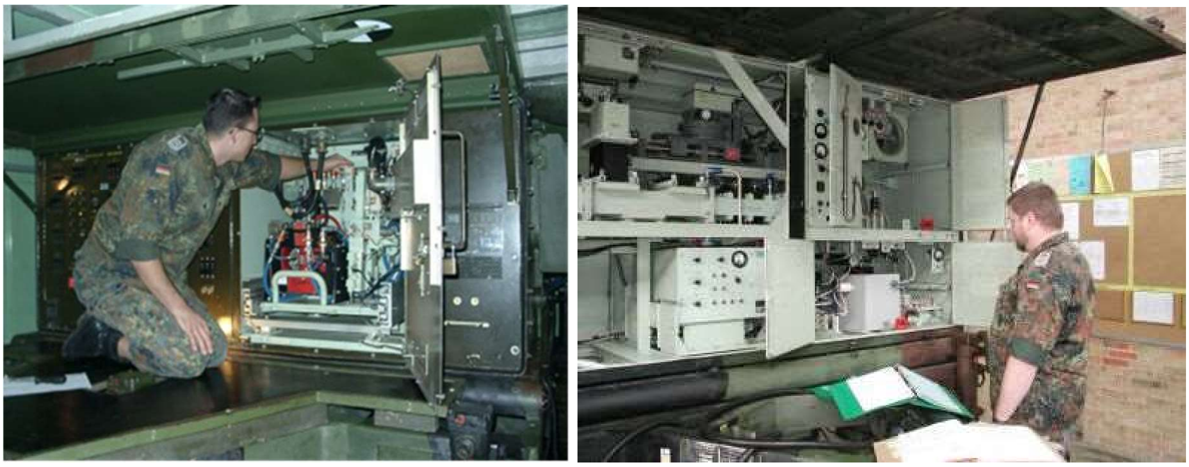

Abb. 5: Typische Arbeitspositionen am HPIR (links) am Klystron und in der Nähe des Klystrons (rot lackiertes Bauteil unter dem linken Unterarm des knienden Technikers) und typische Überwachungsarbeit am PAR (rechts) am geöffneten Modulator mit dem Thyratron etwa in Kopfhöhe des stehenden Technikers

2.3 Radargeräte des Fla-Rak-Systems NIKE

Das Flugabwehr-Raketensystem NIKE wurde als Teil der NATO-weiten Luftverteidigung gegen Luftziele in großen Höhen ab Ende der 1950er Jahre in festen Stellungen entlang einer Nord-Süd-Linie aufgebaut. Es gab in der Nutzungszeit von NIKE mehrere technische Verbesserungsprogramme wie beispielsweise die Einrüstung eines weiteren Tracking-Radar-Gerätes Mitte der 1960er Jahre und die Einrüstung eines Hochleistungs-Rundsuchradargerätes zur Luftlageerstellung auf Bataillonsebene. Das Fla-Raketensystem NIKE wurde 1989 außer Dienst gestellt.

Eine NIKE-Stellung wurde von einer Kampf-Batterie (entspricht einer Kompanie) betrieben. Die Stellung bestand aus zwei räumlich getrennten Bereichen, dem Feuerleitbereich und dem Abschussbereich. Im Feuerleitbereich waren die Radargeräte zur Erfassung der Ziele (LOPAR - Low Power Acquisition Radar), zur Verfolgung der Ziele (TTR-Target Tracking Radar, TRR – Target Ranging Radar) und zur Verfolgung und Steuerung des Flugkörpers (MTR – Missile Tracking Radar) aufgestellt. Im bis zu 4000 m davon befindlichen Abschussbereich wurden die Lenkflugkörper gewartet und auf Startgeräten vorgehalten.

Vier solcher Kampf-Batterien waren zu einem Bataillon zusammengefasst. Auf der Ebene des Bataillons wurde das Hochleistungs-Suchradar (HIPAR – High Power Acquisition Radar) betrieben.

Ab 1980 wurden die Radargeräte systematisch hinsichtlich der Röntgenstörstrahlungsemission untersucht. Im Ergebnis zeigt sich, dass im Abstand von 30 cm von der berührbaren Oberfläche der für technische Arbeiten geöffneten Senderbaugruppen der Trackingradargeräte keine Werte der Ortdosisleistung über dem Nulleffekt gefunden wurden. Eine Dosisbetrachtung ist also nur für die Rundsuchradargeräte (LOPAR, HIPAR) notwendig. Abb. 6 und 7 sind Schemazeichnungen des LOPAR und des HIPAR, das in einem eigenen Betriebsgebäude untergebracht war

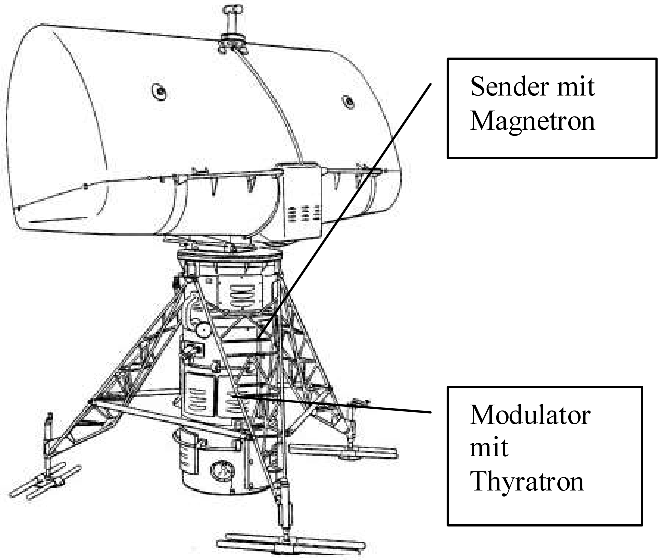

Abb. 6: Gesamtansicht des Ansicht des LOPAR mit Darstellung der Einbaustellen der Röntgenstörstrahler im tonnenförmigen Gehäuse

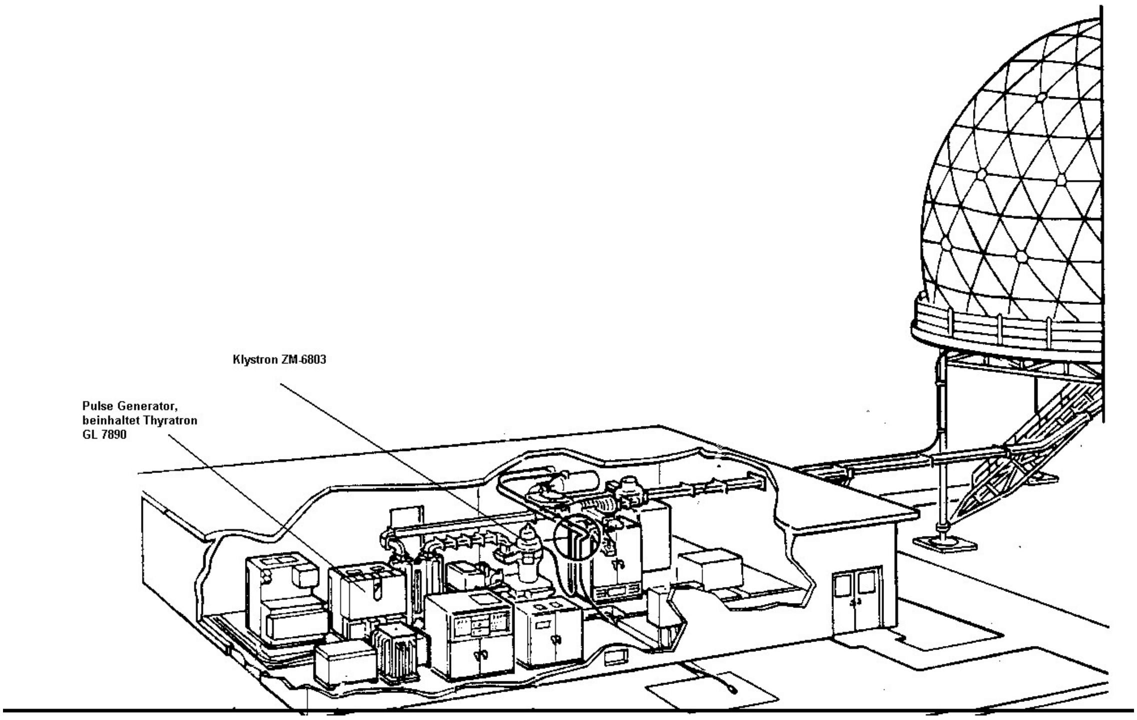

Abb. 7: Gesamtansicht des stationären HIPAR-Gebäudes mit Teil des Radomes der Rundsuchradarantenne/

In Tabelle 5 sind die Röntgenstörstrahler der Radargeräte des Fla-Rak-Systems NIKE angegeben, für die eine Dosisbetrachtung notwendig ist.

Tabelle 5: Liste der zu betrachtenden Störstrahler in den Radargeräten des Fla-Raketen-Systemes NIKE

Nr	Radargerät	Störstrahler	Type	HV
1	LOPAR	Magnetron	5795	36 kV
2		Thyratron	JAN 5948A	16 kV
3	HIPAR	Gleichrichterröhren	F 7779	20 kV*)
4		Thyratron	GL 7890	28 kV
5		Klystron	ZM-6803	200kV

*) die 6-fach vorhandenen Gleichrichterröhren wurden Anfang 1980 durch Halbleiterbauelemente ersetzt.

2.4 Feuerleitradar Deisswil

Das Feuerleitsystem mit dem Feuerleitradar Deisswil wurde zur Steuerung von Flugabwehrkanonen eingesetzt. Die Bahnverfolgung von Wetterballons zur Ermittlung von Windprofilen gehörte ebenso zum Nutzungsspektrum der Radargeräte des Typs Deisswil, von denen unterschiedliche Versionen genutzt wurden. Abb. 8 zeigt die Versionen Deisswil IV und Deisswil VIIB. Der Radarsender der unterschiedlichen Versionen war weitgehend gleich aufgebaut, sodass sich die Arbeitsplatzbetrachtung auf die leistungsfähigste Version Deisswil VIIB beschränken kann[2].

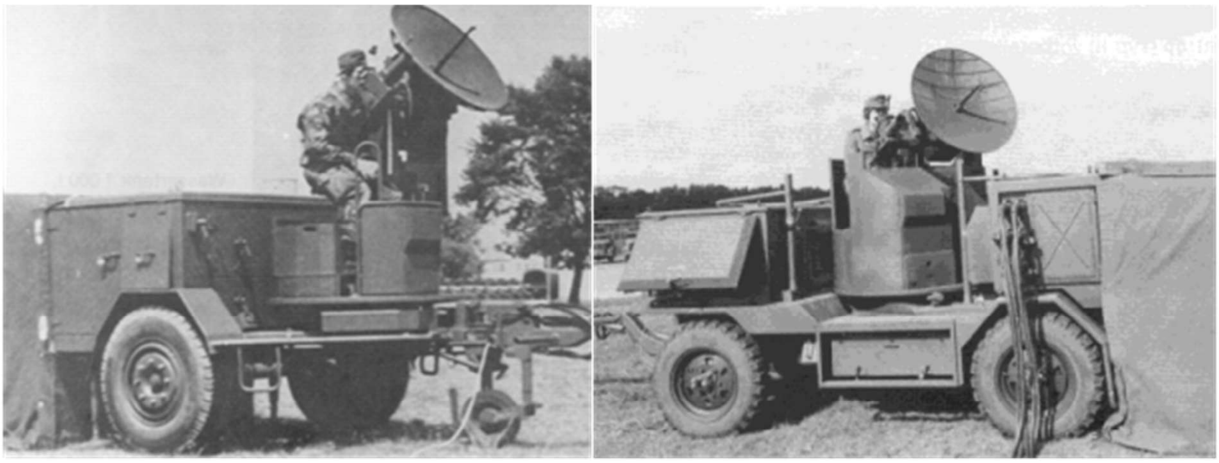

Abb. 8: Feuerleitgeräte der Typen Deisswil IV (links) und Deisswil VII (B) (rechts)

Der Radarsender der Feuerleitgeräte Deisswil war im Richtgerät eingebaut, einer kanzelförmigen horizontal drehbaren Baugruppe, an der die in der Neigung verstellbare Parabolantenne montiert ist. Auf den Richtgerät war zudem der Arbeitsplatz des Richtkanoniers. Weitere Baugruppen z.B. für die Spannungsversorgung des Richtgerätes waren in Schränken auf dem Anhänger untergebracht.

Die im Radarsender des Deisswil VIIB eingebauten Röntgenstörstrahler sind in Tabelle 6 angegeben.

Tabelle 6: Zusammenstellung der im Feuerleitgerät Deisswil VII eingebauten Röntgenstörstrahler

Nr	Einbauort	Bauteil	Typen	Hochspannung
1	Richtgerät	Magnetron	AEELR 01-LD (Litton)<br>VMX 1497 G (Varian)	22 kV
2		Thyratron	5 C 22, HT 415	11 kV
3		Shunt-Diode	8020W	11 kV
4		Clipper-Diode	8020, 8020W	22 kV
5	Geräteschrank	Thyratron	8503<br>CW 6022	11 kV
6		Gleichrichterdiode	PL 5544	5 kV

[2] Im Feuerleitgerät Deisswil VII waren beispielsweise zwei Magnetrons eingebaut, um durch Umschalten sofort eine Ausweichfrequenz verfügbar zu haben.

2.4 Landeanflugradar AN/CPN-4

Das Radargerät AN/CPN-4 mit dem Landekontrollgerät AN/MPN-11 war ein mobiles, auf zwei Anhängern montiertes Radarsystem, das ab Mitte der 1950er Jahre – quasi als Erstausstattung- eingeführt wurde. Der Betriebsanhänger (Operations Trailer) enthielt die kompletten Radargeräte (Präzisionsanflugradar und Rundsuchradar), die Funkausrüstung und die Bildschirmanzeigeeinheiten, an denen die Operatoren arbeiteten. Der Stromversorgungsanhänger (Power Trailer) beinhaltet das Dieselaggregat, das Klimagerät und die Heizung sowie das Ersatzteillager. Abbildung 7 zeigt die beiden Anhänger mit den betriebsbereiten Radargeräten.

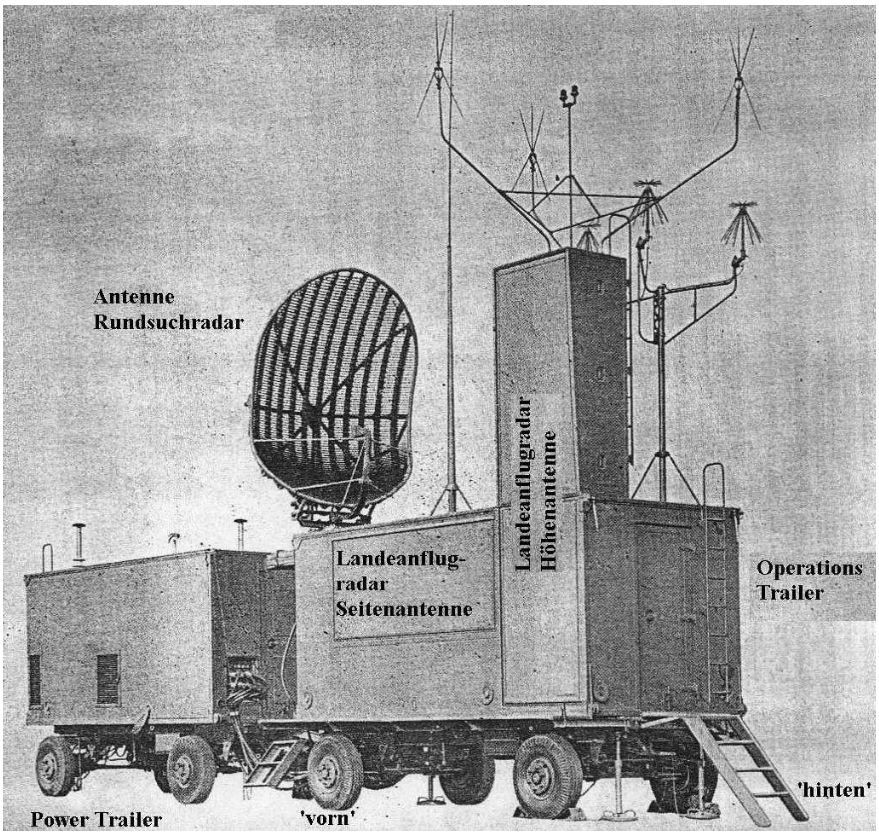

Abb. 9: Außenansicht des AN/CPN-4 bestehend aus Stromversorgungsteil (Power Trailer) und Geräte/Bedienteil (Operations-Trailer) mit den dazugehörigen Antennen des Landekontrollgerätes AN/MPN-11. (Die Angaben 'vorn' und 'hinten' dienen der Orientierung bei der Betrachtung der Abbildungen 10 und 11.)

Die folgenden Abbildungen 10 und 11 zeigen detailliert die Innenansichten des Operations-Trailers mit den Arbeitsplätzen der 3 Operatoren, die sich z.T direkt vor den Sender-/Modulatorbaugruppen der Radargeräte befanden. Durch die Lüftungsschlitze ist bei diesen Radargeräten eine Exposition pa (posterior-anterior – von hinten) zu berücksichtigen.

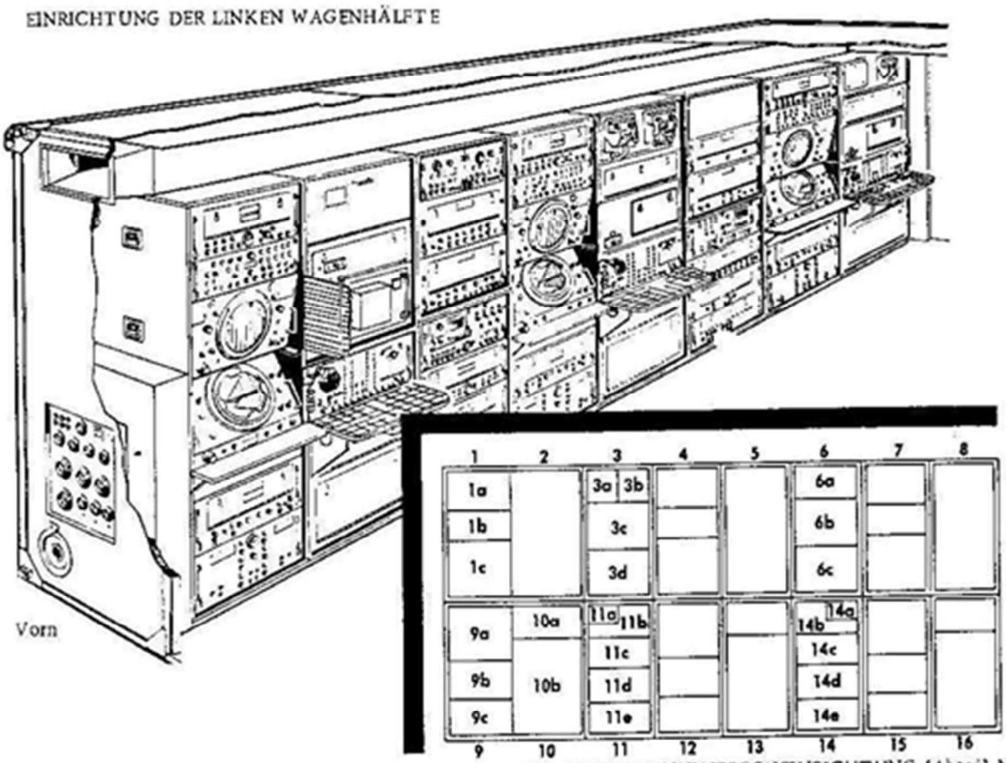

1. SUCHER-BILDSCHIRMANLAGE (Abteil Nr. 14)
   1a. Energieversorgung für Sucher-Bildschirm
   1b. Sucherzentrale
   1c. Sucher-Bildschirmgerät

2. OBERE FUNKSPRECHGERÄTE (Abteil Nr. 11)

3. FERNÜBERTRAGUNGSANLAGE (Abteil Nr. 13)
   3a. Energieversorgung für Kabelverstärker (Suchersystem)
   3b. Energieversorgung für Kabelverstärker (Meßradarsystem)
   3c. Meßradar-Kabelverstärker
   3d. Suchradar-Kabelverstärker

4. Entspricht 1.

5. OBERE FUNKSPRECH-EINRICHTUNG (Abteil Nr. 11A)

6. BILDSCHIRM-SCHALTGERÄT (Abteil Nr. 16)
   6a. Leere Frontplatte
   6b. Bildschirm-Steuerwähler
   6c. Kartengenerator

7. Entspricht 1.

8. OBERE FUNKSPRECH-EINRICHTUNG (Abteil Nr. 11B)

9. AZ-EL-BILDSCHIRMANLAGE (Abteil Nr. 1)
   9a. Az-el-Bildschirmgerät
   9b. Meßradar-Ablenkgenerator
   9c. Energieversorgung für az-el-Bilschirmgerät

10. UNTERE FUNKSPRECHEINRICHTUNG (Abteil Nr. 10)
   10a. Funksprech-Bedienungstafel
   10b. Funksprech-Geräte

11. MTI-ENERGIEVERSORGUNGS-ANLAGE (Abteil Nr. 15)
   11a. Winkelspannungs-Meßgerät
   11b. Sucher-Bildwellenmischer
   11c. Energieversorgung für zusammengesetzten Bildwellengenerator
   11d. MTI-Regler
   11e. MTI-Gleichrichter

12. Entspricht 9.

13. Entspricht 10.

14. ZUSAMMENGESETZTE BILDWELLEN-EINRICHTUNG (Abteil Nr. 17)
   14a. Winkelspannungs-Prüfgerät
   14b. Peilverstärker
   14c. Zusammengesetzter Bildwellengenerator
   14d. MTI-Regler
   14e. MTI-Gleichrichter.

15. Entspricht 9.

16. Enspricht 10.

In der Anordnung der Funksprech-Anlagen bestehen gewisse Unterschiede zwischen dem Radargerät AN/CPN-4 und dem Landekontrollgerät AN/MPN-11.

Abb. 10: Übersicht der Baugruppen im Operations-Trailer mit den 3 Konsolenarbeitsplätzen

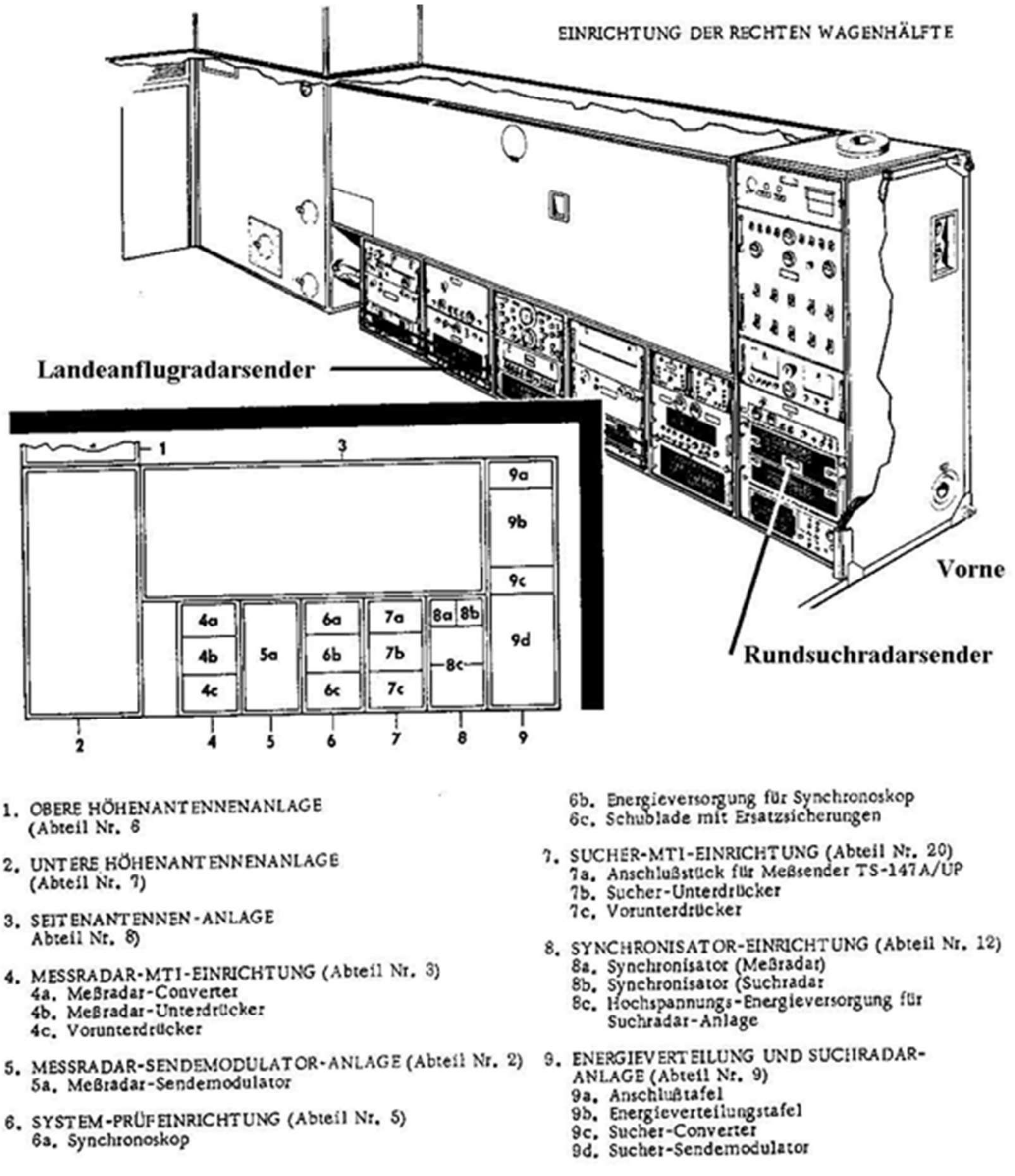

Abb. 11: Ansicht der rechten Seite des Operations-Trailers mit Legende der eingebauten Geräte und Komponenten

Die in den Radarsendern des AN/CPN-4 eingebauten Röntgenstörstrahler sind in Tabelle 7 angegeben.

Tabelle 7: Zusammenstellung der in den Radargeräten des AN/CPN-4 eingebauten Röntgenstörstrahler

Nr	Einbauort	Bauteil	Type	Hochspannung
1	Rundsuchradar	Magnetron	JAN 5586	24 kV
2		Thyratron	JAN 5949/ 1907	16 kV
3	Landeanflugradar	Magnetron	JAN 2J51	15 kV
4		Thyratron	JAN HT 415/ 5C22	8 kV

2.5 Luftraumüberwachungsradar HADR

Die in den Jahren 1982 – 1984 in ortsfesten Radarstellungen des Radarführungsdienstes in Dienst gestellten Luftraumüberwachungsradargeräte des Typs HADR (Hughes Air Defence Radar) dienen der weiträumigen Überwachung des Luftraumes und der Ortung von Luftzielen. Die Luftwaffe betreibt bis zum heutigen Tag 4 Geräte dieses 3D-Rundsuchradargerätetyps, mit dem Richtung, Abstand und Höhe eines Luftzieles bis zu einer nominellen Entfernung von 450 km gemessen werden können.

Für die erforderliche hohe Impuls-Sendeleistung ist im HADR ein mehrstufiger Sender eingebaut, der in mehreren Geräteschränken eingebaut ist, die in einem Radarsenderaum aufgestellt sind, der sich unter dem Radom befindet (Abb. 12). Die in der Anlage eingesetzten Röntgenstörstrahler sind in Tabelle 8 zusammengefasst

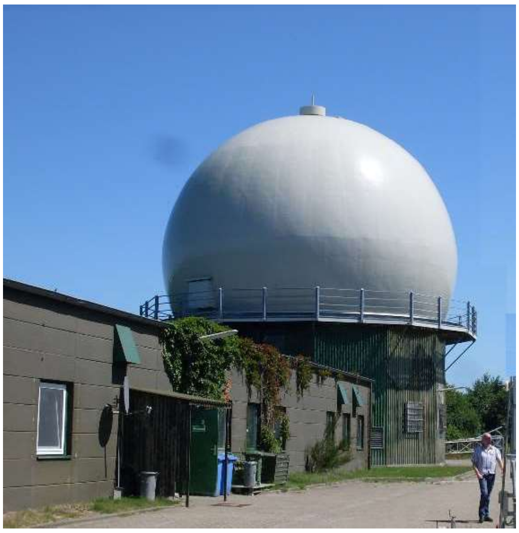

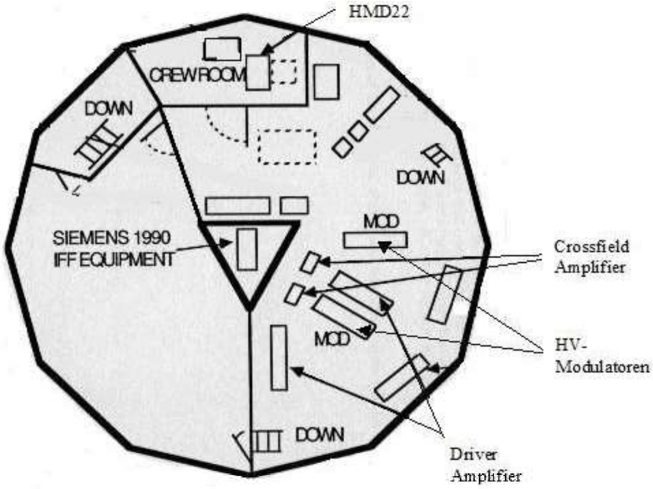

Abb. 12: Außenansicht des Betriebsgebäudes HADR (links) und Anordnung der Senderbaugruppen im Technikraum unter dem Radom (rechts)

Tabelle 8: Zusammenfassung der im HADR eingebauten Röntgenstörstrahler

Nr	Bauteil	Typen	Hersteller	Betriebsspannung
1	Wanderfeldröhre (TWT)	1758H (vor 1984)	Hughes	45kV
2		VTR-5728 (nach 1984)	Varian	45kV
3	Crossed Field Amplifier (CFA)	L-4756A	Litton	35kV
4		QKS 1998 (nach 1984)	Raytheon	35kV
5	Thyratron	CX1549/8047	Hughes	19kV

2.6 Mobiles Rundsuchradar AN/TPS-43

Das AN/TPS-43B ist ein aus zwei Modulen, nämlich einem Container mit der gesamten Elektronik und der Antenne, bestehendes, transportables 3D-Radargerät zur Luftraumüberwachung. Es wurde vom Radarführungsdienst betrieben und oft als Ersatz für die ortsfesten Luftraumüberwachungsradargeräte aufgebaut, wenn diese repariert oder umgerüstet wurden.

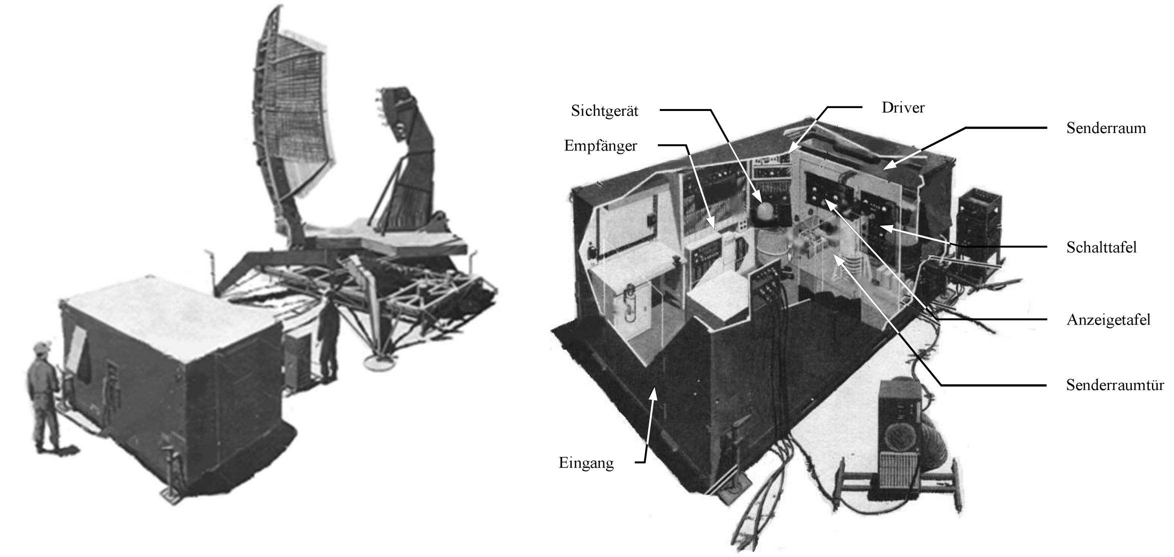

Abb. 13: Ebenerdige Aufstellung der beiden Komponenten des AN/TPS-43 und Schnittbild des Containers

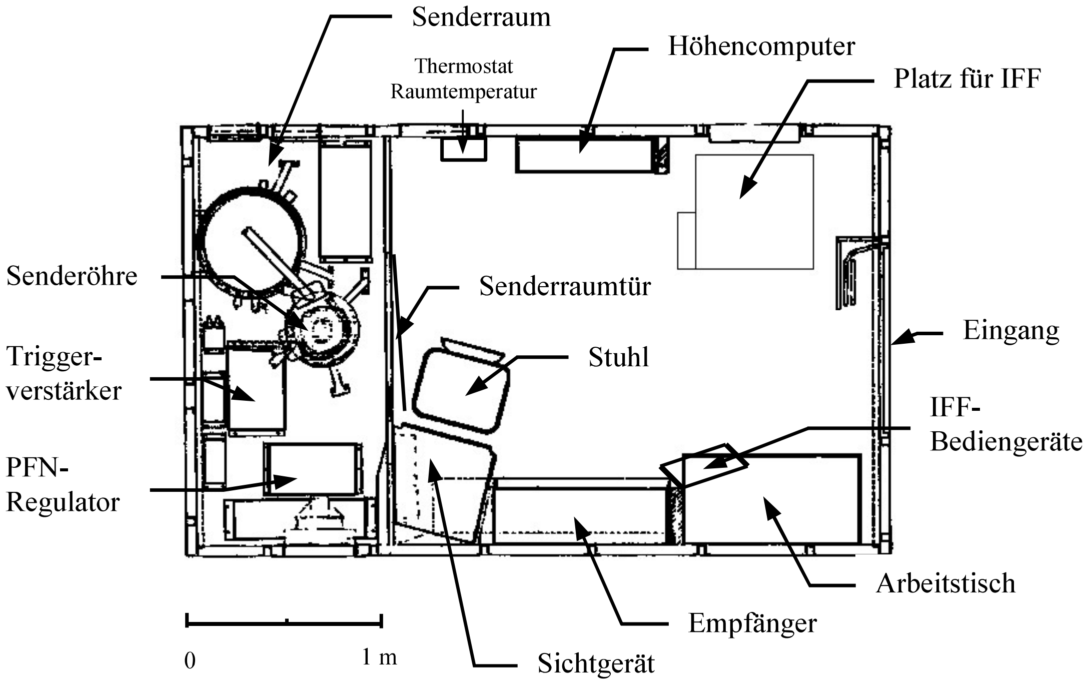

Abb. 14: Lageplan der einzelnen Komponenten im Container

Die im 3D-Rundsuchradar AN/TPS-43 eingebauten Röntgenstörstrahler sind in Tabelle 9 angegeben.

Tabelle 9: Zusammenstellung der im AN/TPS-43 eingebauten Röntgenstörstrahler

**Nr**	**Baugruppe**	**Bauteil (Störstrahler)**	**Typ**	**Betriebsspannung**		
1	Sender	Twystron	VA 145E	117 kV		
2	Modulator	Thyratron (Keramik)	JAN 7390	25 kV		
3	Modulator	Tetrode	ML 7715	25 kV		
4	Driver	TWT (Wanderfeldröhre)	544H	8,1 kV		

2.7 Luftraumüberwachungsradar AN/FPS-7E

Die Rundsuchradargeräte des Typs AN/FPS-7E wurden in ortsfesten Radarstellungen des Radarführungsdienstes der Luftwaffe von Anfang der 60er bis Mitte der 80er Jahre betrieben. Die insgesamt vier Radargeräte wurden vom den 3D-Rundsuchradageräten des Typs HADR abgelöst. Abb. 15 zeigt die Antennenanlage mit der auffälligen Stapelung von Hornstrahlern zur Höhenabtastung des AN/FPS-7, das eines der ersten 3D Rundsuchradargeräte war. Die Angaben zu den Röntgenstörstrahlern sind in Tabelle 10 zusammengefasst.

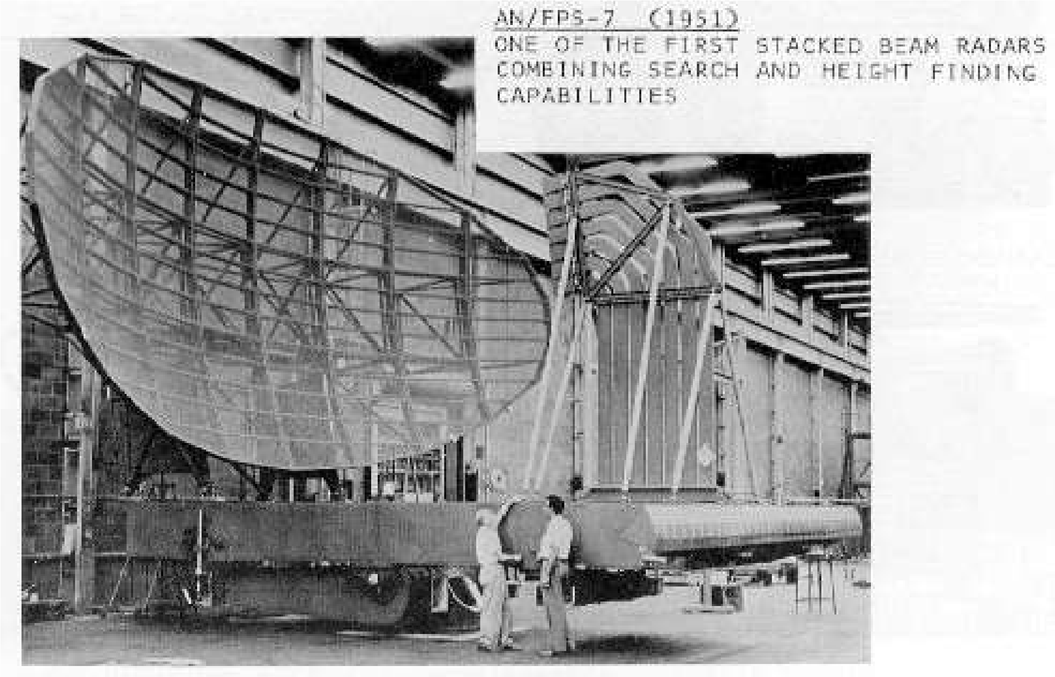

Abb. 15: Antenne des 3D-Rundsuchradargerätes AN/FPS-7 (Quelle: [2]))

Tabelle 10: Einbauorte der Störstrahler des AN/FPS-7E

**Bauteil/Baugruppe**	**Typ**	**Betriebs-spannung**	**Anzahl**
Klystron/Transmitter	L 3250	220 kV	2
Thyratron/Modulator	JAN 5948 A	30 kV	2x6
Charging-, Reverse Voltage Dioden /Modulator		30 kV	2x6
HV-Dioden /DC-Power-Unit	GL 5973, JAN 5973	15 kV	2x6

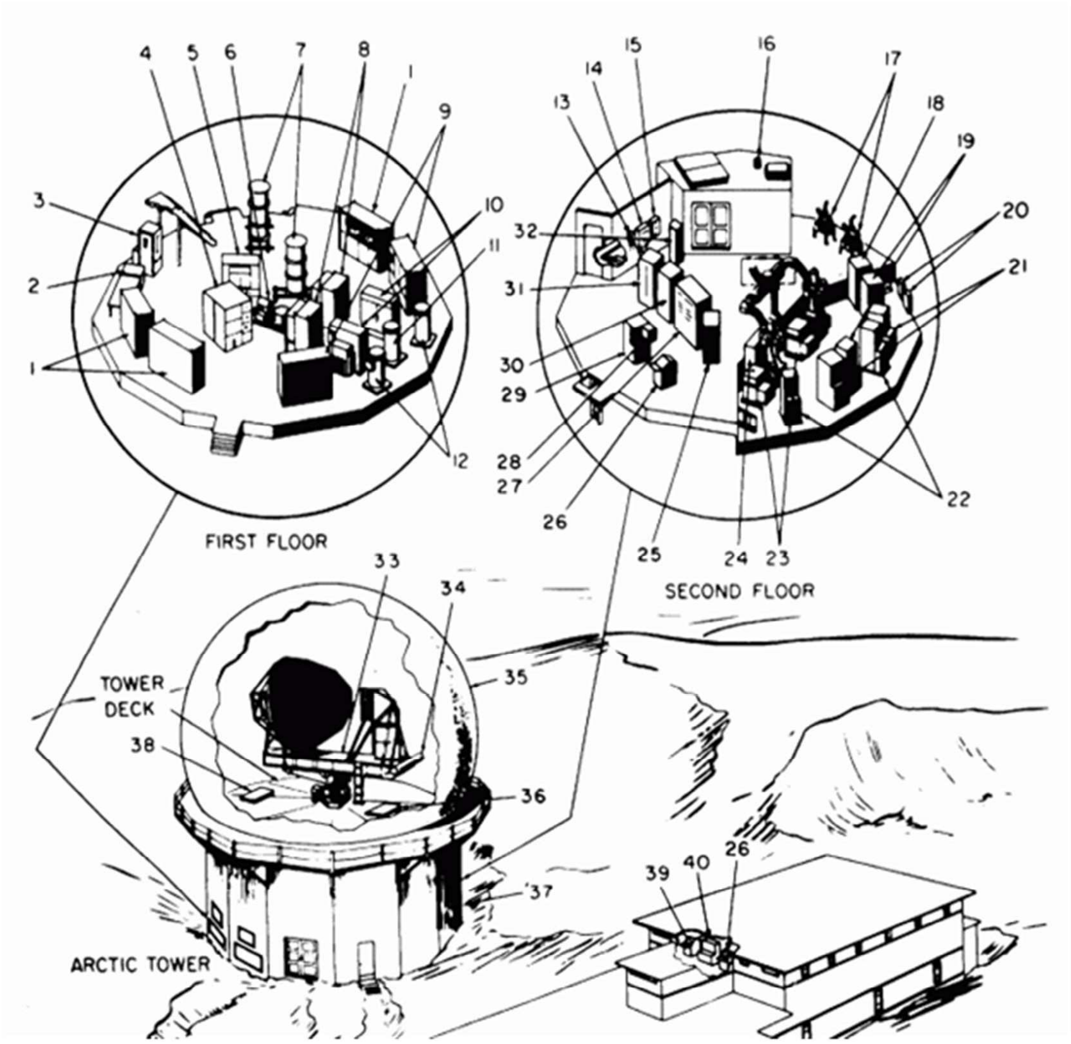

Abb. 16: Gesamtübersicht der Komponenten des AN/FPS-7 in einem typischen Radarbetriebsgebäude. Die Legende der Abbildung ist Tabelle 11.

Tabelle 11: Legende der Abb.16, Bezeichnung der Komponenten der Übersichtszeichnung

1		Air Conditioner AF/F32C-1	18		Panel, Power Distribution SB-704/FPS-7
2		Signal Generator Group OA-1575/FPS-7	19		Control-Monitor Group OA-2296/FPS-7B
3		Amplifier-Oscillator Group OA-2567/FPS-7B	20		Deicing Switchbox
4		Switchboard, Power SB-347/FPS-7	21		Amplifier-Control Group OA-2297/FPS-7B
		(AN/FPS-7B and AN/FPS-7D system serial number 26 only)	22		Amplifier-Oscillator Group OA-2567/FPS-7B
		Switchboard Power SB-1127/FPS-7C	23		Transmitter, Radar T-735/FPS-7B
		(AN/FPS-7D system serial numbers 31 thru 34 only)	24		Junction Box J-865A/FPS-7
5		Dehumidifier-Pressurizer, Waveguide HD-287/FPS-7	25		Moving Target Indicator Group OA-1494A/FPS-7
		(AN/FPS-7B and AN/FPS-7D system serial number 26 only)	26		Indicator Group AN/UPA-35 GFE)
		Dehumidifier-Pressurizer, Waveguide HD-287 A/FPS-7 (AN/FPS-7D	27		Receiver Group OA-2320/FPS-7B
		system serial numbers 31 thru 34 only)	28		Radar Recognition Set AN/UPX-6 (GFE)
6		Dummy Load, Electrical DA-179/FPS-7	29		Amplifier-Power Supply Group OA-2298/FPS-7B
7		Klystron Tube (Spare)	30		Radar Set Group OA-1495A/FPS-7
8		Amplifier-Power Supply Group OA-2298/FPS-7B	31		Radar Set Group OA-1509A/FPS-7
9		Cooler, Liquid, Electron Tube HD-288/FPS-7	32		Control Antenna C-1343/FPS-7
10		Power Supply PP-1735A/FPS-7	33		Antenna Group OA-1999/FPS-7B
11		Regulator, Voltage CN-427/FPS-7	34		Antenna AT-309/GPN(GFE)
12		Regulator, Voltage CN-428/FPS-7	35	*	Radome CW-208D/CPS-6B
13	*	Interconnecting Box J-500/FPS-6	36	*	Electric Space Heater HD-85/CPS-6B
14	*	Control, Inflation, Radome C-2255/FPS-7	37	*	Tower AB-372A/FPS-7
15	*	Control, Inflation, Radome C-1591/FPS-8	38	*	Electric Space Heater HD-86/CPS-6B
16		Control, Antenna C-2347/FPS-7	39		Indicator Group OA-1498/FPS-7 (AN/FPS-7D only)
17	*	Dehumidifier, Desiccant, Electric HD-212/FPS-8	40		Console, Countermeasures OA-2786/FPS-7

* AN/FPS-7D units are GFE. (government furnished equipment)

2.8 Höhenmessradargerät AN/FPS-6

Die Radargeräte des Typs AN/FPS-6 wurden in ortsfesten Radarstellungen des Radarführungsdienstes der Luftwaffe von Ende der 50er bis Ende der 80er Jahre betrieben. Die meisten Geräte waren von den amerikanischen Streitkräften übernommen worden, Hersteller war die amerikanische Firma General Electric. Zeitweise waren bis zu 8 Geräte im Einsatz.

Im Rahmen der Luftlageerstellung dienten sie zur Höhenbestimmung von Flugzielen. Der Einsatz erfolgte immer in Verbindung mit einem Rundsuch- und weiteren Höhenmessradargeräten. Abb. 17 zeigt die Antenne. Bei der Nutzung im Bereich der Bundeswehr waren die Antennen in Radomen aufgebaut und die Senderanlage im Senderaum fest aufgebaut. Abb. 18 zeigt die typische Geräteanordnung in einem Senderraum eines Höhenmessradargerätes, die in diesem Radargerät eingebauten Röntgenstörstrahler sind in Tabelle 12 angegeben.

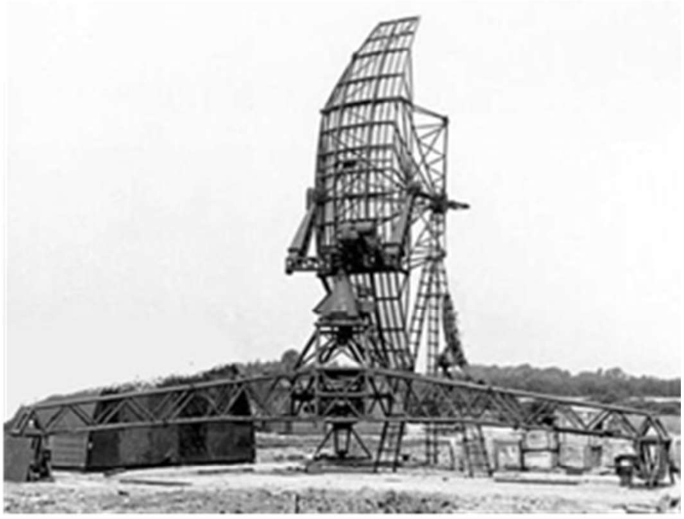

Abb. 17: Antenne eines ebenerdig aufgestellten Höhenmessradargerätes AN/FPS-6

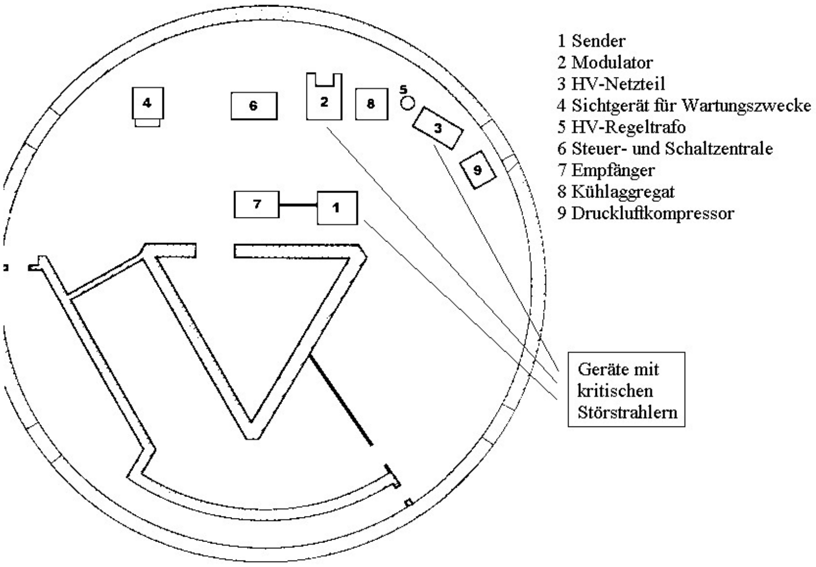

Abb. 18: Typischer Lageplan der Komponenten eines Höhenmessradargerätes AN/FPS-6 in Radarsenderaum unter dem Radom

Tabelle 12: Röntgenstörstrahler im Höhenmessradar AN/FPS-6

Nr	Baugruppe	Störstrahler	Typen	Anzahl	HV
1	Sender	Magnetron	RK 6410A/QK 338A,<br>RK 7529A/QK 327A<br>VMS 1424	1	65 kV
2	Modulator	Thyratron	JAN 5948A,<br>JAN 7390A (Keramik)	1	25 kV
3		HV-Diode	CDZ 576A	2	25 kV
4	HV-Netzteil	HV-Diode	371B	6	12,5 kV

2.9 Höhenmessradar AN/FPS-89

Das AN/FPS-89 ist eine verbesserte Version des AN/FPS-6, wobei am Sender und Modulator keine andern Störstrahler als beim Vorgängertyp zu Einsatz kamen. Die Tabelle 12, in der die Störstrahler zusammengefasst sind, entspricht somit der Tabelle 13. Abbildung 19 zeigt die Ansicht des Senderraumes eines AN/FPS-89.

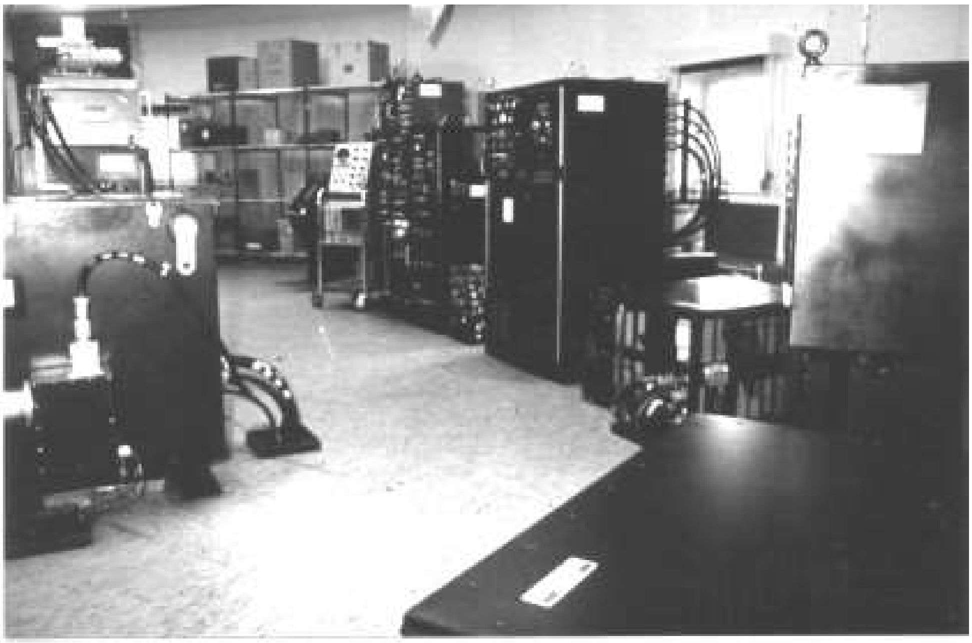

Abb. 19: Ansicht eines Radarsenderaumes eines AN/FPS-89

Tabelle 13: Röntgenstörstrahler im Höhenmessradar AN/FPS-89

Nr	Baugruppe	Störstrahler	Typen	Anzahl	HV
1	Sender	Magnetron	RK 6410A, JAN 6410A, QK 338A, RK 7529A, QK 327A, VMS 1424	1	65 kV
2	Modulator	Thyratron	JAN 5948A, JAN 7390A (Keramik)	1	25 kV
3		HV-Diode	CDZ 576A	2	25 kV
4	HV-Netzteil	HV-Diode	371B	6	12,5 kV

2.10 Höhenmessradar S-244

Die Höhenmessradargeräte des Typs S-244 wurden in ortsfesten Radarstellungen des Radarführungsdienstes der Luftwaffe von Mitte der 60er bis Anfang der 80er Jahre betrieben. Zeitweise waren bis zu 7 Geräte im Einsatz. Hersteller war die britische Firma Marconi.

Im Rahmen der Luftlageerstellung dienten sie zur Höhenbestimmung von Flugzielen, der Einsatz erfolgte immer in Verbindung mit einem Rundsuchradargerät.

Der Sender/Empfänger des Radargerätes S-244 beruhte auf konventioneller Technik (Magnetron, Thyratron). Die verschiedenen Bauteile waren gemeinsam in einem Geräteschrank (Bezeichnung SR 1000; Breite 180 cm, Höhe 195 cm, Tiefe 95 cm) sehr kompakt untergebracht. Für die verschiedenen Frequenzbereiche gab es unterschiedliche Sender-/Empfängerschränke mit verschiedenen Magnetrontypen. Die Angaben zu den Röntgenstörstrahlern sind in Tabelle 14 angegeben.

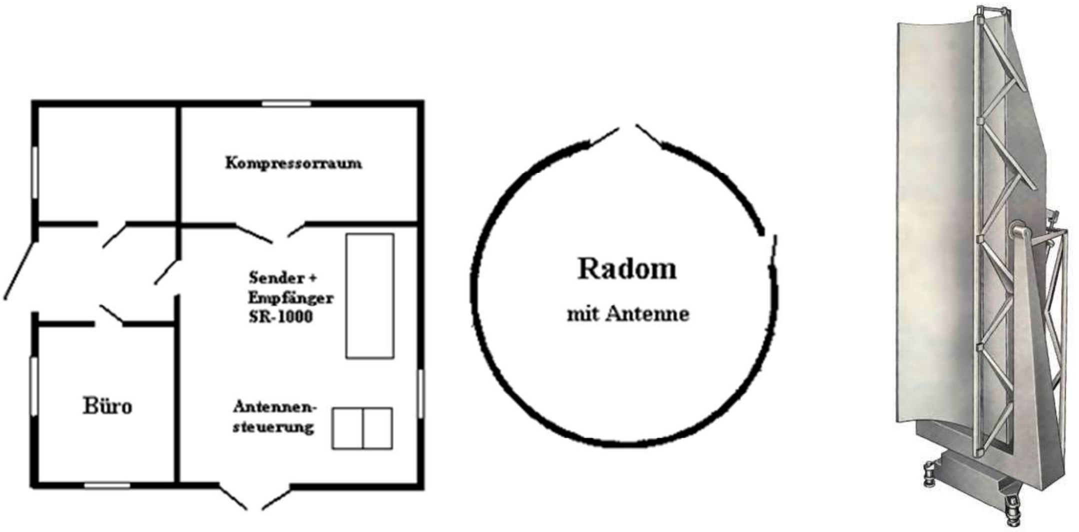

Abb. 20: Typische Anordnung der Baugruppen eines Höhenmessradargerätes S-244 mit Betriebsgebäude und Radom (links) unter den sich die Antenne (rechts) befand.

Tabelle 14: Röntgenstörstrahler im Sender SR 1000 des S-244

**Nr**	**Störstrahler**	**Typen**	**HV**		
1	Magnetron	M 543P …Q; M566P … R; M569P, Q; 570P … R; M5133 – M5136	45 kV		
2	Thyratron	CX 1140; CV 8563	20 kV		
3	HV-Dioden	CV 2160; GXU 3	10 – 20 kV*)		

*) Betriebsspannung abhängig von Einsatz als Lade-/Rückstromdioden im Modulator (20 kV) oder als Gleichrichter im Hochspannungsversorgungsteil (10 kV).

3 Werte der gerätespezifischen monatlichen Körperdosen

Wie in [1] dargestellt, beruhen die in den Ergebnisberichten ermittelten monatlichen Dosiswerte am den Aufenthalts- und Arbeitsstellen die Ausgangswerte der Dosisberechnung auf Maximalwerten. Für einige damalige Dosisangaben ist eine Neubewertung von Arbeitszeitansätzen nach Maßgabe realistischer – anstelle großzügiger - Bewertungen geboten.

3.1 Flugzeugradar NASARR

Die Werte der Photonen-Äquivalent-Ortsdosisleistung H_x[3]sind in folgender Tabelle zusammengefasst.

Tabelle 15: Zusammenstellung der Störstrahler des NASARR und maximale Werte der Ortsdosisleistung (in der Dosisgröße H_x)

**Störstrahler**	**Hoch-spannung [kV]**	**Aufpunkt und Abstand**	**max. Ortsdosis-leistung [µSv/h]**
Magnetron	25	5 cm oberhalb Mikrowellenausgang	300
		30 cm oberhalb Mikrowellenausgang	10
Thyratron	12	5 cm Abstand zum Thyratron	100
		an Oberfläche des Sendergehäuses	30

Zur Emission der Röntgenstrahlung am NASARR liegen eine Reihe von Messberichten verschiedener Dienststellen der Bundeswehr vor, die auch den Nutzungsbeginn Anfang der 1960-er Jahre abdecken. Die für die Arbeitsplatzbewertung relevante Emission stammt vom Magnetron. Dor tritt sie an der für diesen Störstrahler typischen Stelle der Mikrowellenleiterauskopplung an die metallische Kavität im Joch des Permanentmagneten aus.

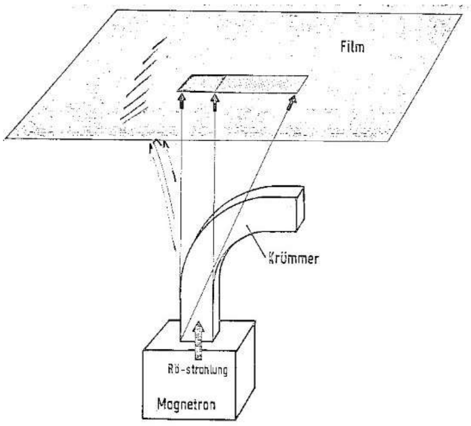

Abb. 21: Schemazeichnung zur Auswertung eines am Magnetron des NASARR exponierten Filmes zur Erfassung der Emissionscharakteristik.

[3] In der Nutzungszeit des NASARR und der Durchführung der Messungen war die Dosisgröße H_x die im operativen Strahlenschutz benutzte Größe.

Abb. 21 zeigt das Ergebnis der Untersuchung der Richtungsverteilung der Röntgenstörstrahlung am NASARR. Aus der Einbauposition ergibt sich, dass eine Exposition durch den räumlich begrenzten Strahl nur oberhalb des Radargerätes möglich ist, betroffene Körperteile sind also Hände und Unterarme sowie Kopf, Hals und obere Regionen des Körperstammes.
Die Erfassung der im Emissionsbereich durchgeführten Arbeiten am NASARR erfolgte durch Befragungen und Einschätzungen von Zeitzeugen und im Rahmen mehrerer Ortstermine an nicht mehr in Nutzung befindlichen aber vollständig erhaltenen (und z.T. betriebsbereiten) Systemen. In deren Ergebnis ist festzustellen, dass der in Teilbericht der Arbeitsgruppe Aufklärung der Arbeitsplatzverhältnisse Radar (AG Radar) angegebene Zeitansatz die tatsächlichen Verhältnisse erheblich überschätzt. Z.B. ist die zeitaufwändige Einstellarbeit an der Automatischen Frequenzkontrolle des Empfängers nicht mit einer Exposition verbunden. Diese Arbeitszeit ist bereits die Hälfte der damals angesetzten Expositionszeit. Eine Halbierung der damaligen Zeitansätze ist als realistisch anzusehen. Aus dem Maximalwerten der Messungen ergeben sich die in Tabelle 16 zusammengestellten Werte.

Tabelle 16: Maximale monatliche Ortsdosis H_x durch Röntgenstrahlung durch Arbeiten am NASARR. Hochspannung des relevanten Störstrahlers: 25 kV

**Nr**	**Ort des Körperteils**	**maximale monatliche Ortsdosis [H_x]**
1	Hände	2400 µSv
2	Unterarme	605 µSv
3	Kopf und Oberkörper	40 µSv

Die am NASARR ermittelten Werte der Ortsdosisleistung zeigen eine breite Verteilung. Die aus 20 Werten von ODL-Messungen, die auch verschiedene Abstände *r* zum Magnetron erfassten, ergeben sich nach Umrechnung gemäß $1/r^2$-Abstandsabhängigkeit auf den Referenzabstand von 40 cm für den Mittelwert und den Median die in Tabelle 17 angegebenen Werte.

Tabelle 17: Zusammenstellung monatlicher Dosiswerte für Arbeiten am NASARR

**Nr**	**Größe**	**Dosisleistung H_x/h [µSv/h]**	
1	Ausgangswert der Dosisbetrachtung (Tabelle 14 Nr 1) bezogen auf 40 cm Abstand	4,68	
2	Mittelwert	3,3 ± 5,8	
3	25%-Perzentil	0,63	
4	Median	1,17	
5	75% Perzentil	2,66	

Damit sind die in Tabelle A1 angegebenen Werte der Teilkörperdosen bei Bezug auf den Medianwert der vorliegenden Messungen um den Faktor 1,17/4,68 = 0,25 zu reduzieren und im Ergebnis sind für Techniker, die am NASARR gearbeitet haben durch Exposition gegenüber Röntgenstörstrahlung als Medianwert der monatlich rezipierte Dosis 5,15 µSv für die effektive Dosis anzusetzen, die Hände (Haut) erhalten als Medianwert eine monatliche Dosis von 370 µSv, die Schilddrüse 4,77 µSv. Durch die Emission des Magnetrons nach oben ist eine Exposition der Keimdrüsen am NASARR nicht vorgekommen.

3.1 Radargerät HIPR des FlaRakSystemes HAWK

Aus der Nutzungszeit des Waffensystems HAWK gibt es ein systematisches flächendeckendes Untersuchungsprogramm der Röntgenstörstrahlung der Radargeräte des Waffensystems HAWK. Eine Dosisbetrachtung ist demnach nur an den Rundsuchradargeräten PAR und den Zielbeleuchtungsradargeräten HPIR in den Rüstungsständen BASIC-HAWK und I-HAWK notwendig. In Tabelle 18 sind die Maximalwerte der Ortsdosisleistung für das HPIR angegeben

Tabelle 18: Maximalwert der Ortsdosisleistung am Störstrahler HPIR

**Nr**	**Basic-HAWK, I-HAWK, Klystron, 12 kV**				
	**Abstand**	**ODL**	**betroffene Organe**	**Expositions-zeit h/Monat**	**Max.Wert der monatlichen Dosis**
1	5 cm	80 µSv/h	Hände	5 h	0,52 mSv[1])
2	30 cm	2,5 µSv/h	Körperstamm	48 h	0,12 mSv

[1]) Der Dosiswert setzt sich zusammen aus dem Wert für den Körperstamm (Zeile 2) und dem Beitrag durch die insgesamt 5h Aufenthalt der Hände am Klystron

Die sich daraus ergebenden Maximalwerte von Teilkörper und Organdosiswerten sind in Tabelle A2 zusammengestellt.

Zum HPIR des Waffensystems HAWK liegen systematische Messungen der Ortsdosisleistung am Klystron vor. Auch hier zeigt sich eine breite Verteilung von Werten, die Angaben zur Statistik sind in Tabelle 19 angegeben.

Tabelle 19: Statistik der Messwerte am HPIR des Waffensystems HAWK

**Nr**	**Größe**	**Dosisleistung H_x/h [µSv/h]**	
1	Ausgangswert der ODL für die Dosisbetrachtung (Tabelle 16 Nr 1)	80	
2	Mittelwert	8,41 ± 13,16	
3	25%-Perzentil	1,60	
4	Median	5,00	
5	75% Perzentil	8,00	

Unter Zugrundelegung des Medianwertes (5,0 µSv/h) der Messwerteverteilung anstelle des Maximums (80 µSv/h) sind die Angaben der Tabelle mithin um den Faktor 16 zu reduzieren.

Als monatlich rezipierte Dosis ist (als effektive Dosis) der Wert 6,1 µSv zu Grunde zu legen, für die Keimdrüsen ist der Medianwert der monatlichen Dosis 5,7 µSv.

3.3 Radargeräte des Fla-Rak-Systems NIKE

Das Ergebnis der Sichtung der ab 1980 systematisch durchgeführten Ortdosismessungen an den Radrgeräten von NIKE ist in Tabelle 20 angegeben.

Tabelle 20: Maximalwerte der auf das Jahr bezogenen Ortsdosis an den Rundsuchradargeräten des Fla-Raketensystems NIKE

**Nr**	**Radargerät**	**Störstrahler**	**HV**	**Betroffene Körperteile**	**Jahresdosis H_x**	
1	LOPAR	Magnetron	36 kV	Hände, Unterarme	0,3 mSv	
2	HIPAR	Thyratron	28 kV	Hände, Unterarme	1,4 mSv	
3		Thyratron	28 kV	übriger Körper	0,2 mSv	
4		Klystron	200 kV	Hände, Unterarme	1 mSv	
5		Klystron	200 kV	übriger Körper	0,2 mSv	

Zur Berechnung der Teilkörper- und Organdosiswerte wird auf die Tabelle A3 in Anhang verwiesen, wo die Berechnung für die monatlich rezipierten Dosen durchgeführt wird. Eine für Dosisbetrachtungen relevante Exposition ergibt sich nur für technische Arbeiten an HIPAR. In Tabelle 21 sind die aus den Maximalwerten errechneten Werte für die Effektive Dosis und die Dosis für die Keimdrüsen angegeben.

Tabelle 21: Werte der aus den Maximalwerten errechneten monatlich rezipierten Effektiven Dosis und der Dosis der Keimdrüsen bei Tätigkeiten an HIPAR

**Nr**	**Dosis, betroffenes Organ**	**Maximalwert der monatlichen Dosis**	
1	Effektive Dosis	24,9 µSv	
2	Keimdrüsen	41,1 µSv	

Die Dosisangabe ergibt sich aus einer Summe von Dosisbeiträgen mit unterschiedlichen Photonenenergien. Dem müsste eine Umrechnung der Maximalwerte auf Medianwerte der entsprechenden Messwertreihen Rechnung tragen.

3.4 Feuerleitgerät Deisswil

Bei technischen Arbeiten am geöffneten Senderschrank des Richtgerätes ist eine Exposition des gesamten Körperstammes durch die im unteren Teil des Schrankes an der Vorderseite eingabauten Clipperdioden zu berücksichtigen, Wegen der bei Arbeiten an Vollsystem notwendigen gebückten Körperhaltung ist eine Exposition auch des Kopfes zu berücksichtigen. In Tabelle 22 sind die Ausgangswerte der auf weitgreifenden Annahmen zur Arbeitszeit ermittelten Ortsdosiswerte angegeben.

Tabelle 22: Maximale Werte der monatlichen Ortsdosis in 30 cm Abstand vom geöffneten Sender des Deisswil VII B (Betriebsspannung 22 kV). Exponierte Körperbereiche sind der Körperstamm in 130 cm Höhe sowie Kopf und Hände

**Nr.**	**Personenkreis**	**Expositions-zeit pro Monat [h]**	**maximale monatliche Ortsdosis H_x [µSv]**		
1	2	3	4	5	
			2 Clipperdioden	1 Clipperdiode	
1	InstDste	12	1371	960	
2	InstTr	20	2285	1600	

Hinweise zu Tabelle 22:

Spalte 2: Die mit Exposition verbundenen Arbeiten am Sender des Richtgerätes wurden entweder in Instandsetzungsdiensten (InstDste) oder von (höher qualifizierten und besser ausgestatteten) Einrichtungen der Instandsetzungstruppe (InstTrp) durchgeführt, was zu unterschiedlichen Expositionszeiten führt.

Spalte 4 und 5: In der Arbeitsplatzuntersuchung der AG Radar wurde angenommen, dass eine Exposition durch die Überlagerung des Strahlenfeldes zweier Clipperdioden zu berücksichtigen ist. Diese Annahme ist falsch, es wird immer nur eine Clipperdiode, die zum jeweils betriebenen Radarsender gehört, mit Hochspannung beaufschlagt. Nach Maßgabe der Heranziehung von Maximalwerten sind die Werte der Spalte 5 gültig.

Die aus dem Maximalwert der Ortsdosis für Personal der Instandsetzungstruppe ermittelten Teilköper- und Organdosiswerte sind in Tabelle A4 im Anhang angegeben. Als Wert der monatlichen Dosis ergibt sich (als Effektive Dosis) der Wert 12,2 µSv.

Die aus den Messberichten zum Radargerät des Deisswil ermittelten Daten zur Statistik sind in der folgenden Tabelle 23 zusammengefasst:

Tabelle 23: Statistik der Messwerte am Radarsender des Richtgerätes Deisswil VII B

**Nr**	**Größe**	**Dosisleistung H_x/h [µSv/h]**	
1	Ausgangswert der ODL für die Dosisbetrachtung (Tabelle 18 Nr 1, Spalte 5)	80	
2	Mittelwert	2,46 ± 10,6	
3	25%-Perzentil	0,028	
4	Median	0,090	
5	75% Perzentil	0,389	

Die aus dem Maximalwert der ODL errechneten Maximalwerte der Tabelle A4 sind bei Zugrundelegung des Medianwertes der Messwerte mithin um den Faktor 0,09/80 = 0,0011 zu reduzieren.

Als Wert der monatlichen Dosis (effektive Dosis) ist ein Wert unter 0,01 µSv anzusetzen für die Keimdrüsen ergibt sich der Wert 0,043µSv.

3.5 Landeanflugradar AN/CPN-4

Durch den vergleichsweise kompakten Aufbau des verlegefähigen Landeanflugradars mit den Arbeitsplätzen der Operatoren direkt an den Sender-/Modulatorbaugruppen sind beim AN/CPN-4 auch für Operatorentätigkeiten Expositionen durch Röntgenstörstrahlung zu berücksichtigen. In Tabelle 24 ist das Ergebnis der Arbeitsplatzuntersuchungen zusammengefasst.

Tabelle 24: Maximale Werte der Ortsdosisleistung und der monatlichen Ortsdosis der Röntgenstörstrahlung aus Störstrahlern an Aufpunkten vor dem Sender des Rundsuchradargerätes (Spannung des Störstrahlers 16 kV). Die vertikale Ausdehnung des Strahlenbündels reicht bis max. etwa 50 cm über Boden.

**Nr.**	**Personenkreis/ Tätigkeit**	**Ab-stand**	**exponierte Körperteile**	**Ortsdosis-leistung**	**max. monatliche Dosis H_x**
1	Radartechniker	30 cm	Beine/Unterschenkel, ggf. Körperstamm, bis ca. 50 cm Höhe über Boden (Arbeitshaltung stehend, Strahlenfeld ap)	150 µSv/h	1,5 mSv
2	Radartechniker	30 cm	Kopf, Oberkörper bis ca. 50 cm Höhe über Boden (nur bei hockender Arbeitshaltung, Strahlenfeld ap)	150 µSv/h	1,5 mSv
3	Radartechniker	15 cm	Hände *)	600 µSv/h	6 mSv
4	Operator (auf Platz 1)	40 cm	Beine, Körperstamm, bis ca. 50 cm Höhe über Boden (Arbeitshaltung sitzend, Strahlenfeld pa)	84µSv/h	3,3 mSv
5	Operator (auf Platz 2)	180 cm	Beine, Körperstamm, bis 50 cm Höhe über Boden (Arbeitshaltung sitzend, Strahlenfeld pa)	4 µSv/h	0,16 mSv
6	Operator (Zusammenfassung Platz 1 und Platz 2)	-	dito	-	3,5 mSv

*) Die Hände befanden sich hauptsächlich an den Einstellelementen an der Oberfläche der Sender/Modulatorbaugruppe.

Die für Radartechniker an AN/CPN-4 ermittelten maximalen Werte der Organ- und Teilkörperdosiswerte sind in Tabelle A4 angegeben. Als monatlich rezipierte Effektive Dosis ergibt sich der Wert 0,18 mSv, für die Keimdrüsen ergibt sich ein Wert von 0,24 mSv.

3.6 3D-Rundsuchradar HADR

An den Komponenten der Senderanlage des HADR ist bei technischen Arbeiten an der TWT des Treiberverstärkers bis zum technischen Rüststand 1984, also bei den ersten Geräten, eine Dosisbetrachtung geboten. Die Werte der Ortsdosisleistung und die Monatliche Aufenthaltsdauer an der betreffenden Stelle sind in Tabelle 25 angegeben.

Tabelle 25: Maximalwerte der Ortsdosisleistung (ODL) und Ortsdosis am Treiberverstärker des HADR

**Nr**	**Abstand**	**Betroffene Körperteile**	**ODL**	**Maximale monatliche Dosis H_x**	**Hoch-spannung**
1	30 cm	Oberkörper	110 µSv/h	110 µSv	45 kV
2	5 cm	Hände, Unterarme	300 µSv/h	300 µSv	45 kV

Mit einer 1984, also bereits im Anfang der Nutzungsphase des HADR wurde der Röhrentyp der TWT gewechselt und es wurden keine Werte der Röntgenstörstrahlung über dem messtechnischen Untergrund festgestellt.

Der Vollständigkeit halber sind in Tabelle A6 die sich aus Tabelle 21 ergebenden Teilkörper und Organdosiswerte angegeben. Bis 1984 sind für einen Radartechniker am HADR als monatlich rezipierte Effektive Dosis der Wert 31 µSv anzusetzen, aus der Bestrahlungsgeometrie ergibt sich keine Exposition der Keimdrüsen.

3.7 3D-Rundsuchradar AN/TPS-43

Dominierend für die Expositionsverhältnisse am AN/TPS-43 war die mit 117 kV betriebe Twystron-Endstufe. Zum Röntgenstörstrahlenschutzes wurde an der Tystronröhre eine Abschirmung angebaut und die Tür zum Senderaum (Abb.14) mit einer Abschirmung versehen.

Bei der Wartung des mobilen 3D-Radargerätes sind sowohl häufige Arbeiten als auch seltenere Arbeiten mit Exposition vorgekommen, sodass die Dosisanteile der Arbeitsplatzuntersuchung für monatliche Dosiswerte für häufige Arbeiten und jährliche Dosiswerte für die seltenen Arbeiten. ausgewiesen wurden.

In den eingehenden Untersuchungen von technischen Änderungen waren Hinweise auf eine nicht immer optimale Positionierung der Zusatzabschirmung des Twystrons gefunden worden. Entsprechend kann bei den Arbeitsplatzanalysen unterschieden werden zwischen korrekt angebrachter und defekter Twystronabschirmung. Eine Zuordnung wann welcher Fall im Beschäftigungszeitraum vorgelegen hat, ist allerdings nicht nachvollziehbar.

In Tabelle 26 sind die auf verschiedene Teilkörperregionen bezogenen Maximalwerte der monatlichen Dosis zusammengestellt, wobei die Jahresdosen für unregelmäßige Arbeiten Gleichmäßig (i.e. zu 1/12) auf die monatlichen Dosiswerte verteilt werden.

Tabelle 26: Maximalwerte der Ortsdosis und betroffene Körperregionen bei technischen Arbeiten am AN/TPS-43. Die ODL wird von der Endverstärkerröhre (Twystron) bestimmt, das mit 117 kV betrieben wurde.

**Nr**	**Körperteile**	**Be-strahlung**	**Maximale monatliche Ortsdosis**				
			**bis 1981**	**1982-1984**	**ab 1985**	**Abschir-mung defekt**	
1	Beine bis 50 cm Höhe	pa	4,00 mSv	2,44 mSv	0,10 mSv	19,2 mSv	
2	Körperstamm ab 50 cm Höhe	pa	2,48 mSv	0,113 mSv	0,10 mSv	3,6 mSv	
3	Hände		5,61 mSv	0,25 mSv	0,055 mSv	7,7 mSv	

Den verschiedenen Nutzungsphasen und der Feststellung, dass hohe Werte der ODL auf Defekten der Twystronabschirmung beruhen können, sind bei den maximalen monatlichen Dosiswerten ebenfalls Nutzungszeiträume und technische Gegebenheiten zu Berücksichtigen. Zu den Berechnungen wird auf Tabelle A6 verwiesen, das Ergebnis ist in Tabelle 27 zusammengefasst

Tabelle 27: Maximalwerte monatlicher Dosiswerte für Techniker, die am AN/TPS-43 gearbeitet haben.

**Nr**	**Dosis**	**Zeitraum, Konfiguration**				
		**bis 1981**	**1982-1984**	**ab 1985**	**Abschirmung defekt**	
1	Effektive Dosis	2,1 mSv	0,11 mSv	0,08 mSv	2,93 mSv	
2	Keimdrüsen	0,30 mSv	0,17 mSv	0,10 mSv	3,63 mSv	

3.8 AN/FPS-7E

Wie beim AN/TPS-43 konnte auch beim AN/FPS-7E als Ergebnis der Arbeitsplatzanalyse für Arbeiten mit Exposition gegenüber Röntgenstörstrahlung ein Anteil von regelmäßigen Arbeiten identifiziert werden, für den eine monatliche Dosis angegeben werden kann und ein Anteil unregelmäßiger, seltener vorkommender Arbeiten gefunden werden, für den eine Jahresdosisangabe adäquat ist. Auch hier wird der Dosisanteil der seltenen Arbeiten regelmäßig auf das Arbeitsjahr verteilt und für diese Arbeiten eine monatliche Dosis berechnet.

Maßgeblich beim AN/FPS-7E ist die Störstrahlung aus einem Hochleistungsklystron (220 kV) und den Thyratron-Röhren der Modulatoren (30 kV). Wegen der unterschiedlichen Konversionsfaktoren werden die Dosisanteile getrennt berechnet und addiert. Die Ausgangswerte sind in Tabelle 28 angegeben.

Buch beim AN/FPS-7E haben technische Änderungen während der Nutzungszeit zu Reduktionen der Röntgenstörstrahlungsemission geführt, sodass für die Dosisberechnung unterschiedliche Zeiträume zu berücksichtigen sind.

Tabelle 28: Maximale **monatliche** Ortsdosiswerte und zugehörige Hochspannung der Röntgenstörstrahlung bei technischen Arbeiten am AN/FPS-7E.

**Nr**	**Aufpunkt/ betroffene Körperbereiche**	**Hoch-spannung**	**maximale monatliche Ortsdosis H_x**		
			**bis 1977**	**von 1978-1981**	**ab 1982**
1	0,80 – 1,55 m Höhe Kopf und Körperstamm	30 kV	0,254 mSv	0,222 mSv	keine *
2	Hände		6,4 mSv	5,61 mSv	0,0063 mSv
3	1,60 m Höhe Kopf und Körperstamm	220 kV	0,0226 mSv	0,0226 mSv	0,0226 mSv
4	Hände		0,115 mSv	0,115 mSv	0,115 mSv

* keine Dosis über dem natürlichen Untergrund

In den verschiedenen Nutzungsphasen sind also bei technischen Arbeiten unterschiedliche Dosiswerte bei Arbeiten am Modulator vorgekommen. Das Ergebnis ist in Tabelle 29 angegeben, die Einzelwerte sind in Tabelle A8.

Tabelle 29: Werte der maximalen monatlichen Körper/Teilkörper-Dosiswerte bei Arbeiten am AN/FPS-7E

**Nr**	**Dosis**	**Nutzungszeitraum**			
		**bis 1977**	**1977-1981**	**ab 1981**	
1	Effektive Dosis	0,158 mSv	0,141 mSv	0,0227 mSv	
2	Keimdrüsen	0,276 mSv	0,241 mSv	-	

3.9 AN/FPS-6

In der vergleichsweise langen Nutzungsphase des AN/FPS-6, von dem verschiedene Versionen in der Nutzung waren, hat es insbesondere am Modulator und der Hochspannungsversorgung mehrere Änderungen gegeben, die sich auf die Ortsdosisleistung an den Baugruppen und somit auf die Dosis Beschäftigter auswirken. Bei der Analyse der Arbeitsplatzverhältnisse wurde auch auf Messwerte an den technisch gleich aufgebauten Sendern anderer Radargeräte zurückgegriffen. Das Ergebnis ist in Tabelle 30 zusammengefasst.

Tabelle 30: Zusammenfassung der errechneten maximalen monatlichen Ortsdosiswerte bezogen auf Geräteschränke am AN/FPS-6 für verschiedene Jahre der Nutzung

**Aufenthaltsort**		**maximale monatliche Ortsdosis in mSv**						
**Zeilen-Nr.**	**betroffene Körperteile**	**bis 1974**	**1975-1976**	**1977**	**1978-1980**	**1981-1982**	**ab 1983**	**Spannung in kV**
**Sender, Magnetron**								
1	Hände, Unterarme	42,6	1,99	0,09	0,09	0,05	0,02	**65**
2	Kopf, Oberkörper	6,84	0,08	0,07	0,07	0,005	0,003	
3	Beine Rumpf, Gonaden	3,81	---*	---*	---*	---*	---*	
**Modulator, Thyratron, Rückstromdioden**								
4	Hände, Unterarme	1,25	1,25	1,25	0,34	0,34	0,02	**25**
5	Kopf, Oberkörper	0,3	0,3	0,3	0,03	0,03	0,002	
6	Beine Rumpf, Gonaden	1,5	1,5	1,5	0,2	0,03	0,002	
**Hochspannungsnetzteil, HV-Dioden**								
7	Hände, Arme	< 0,001	< 0,001	< 0,001	keine **	keine **	keine **	**12,5**
8	Kopf, Oberkörper	< 0,001	< 0,001	< 0,001	keine **	keine **	keine **	

Anmerkung: Aus Gründen der schnelleren Vergleichbarkeit wurden alle Dosiswerte in derselben Größeneinheit aufgelistet.

* keine Ortsdosis über der natürlichen äußeren Strahlenexposition

** keine Störstrahler mehr vorhanden

Zur Berechnung von Teilkörper- und Organdosiswerten wird auf Tabelle A9 im Anhang verwiesen. Für die Nutzungsphasen wurden für Techniker die in Tabelle 31 angegeben Werte der maximalen, pro Monat rezipierten Dosiswerte als Effektive Dosiswerte ermittelt.

Tabelle 31: Werte der maximalen monatlichen Körper/Teilkörper-Dosiswerte bei Arbeiten am AN/FPS-6

**Nr**	**Dosis**	**Nutzungszeitraum**					
		**bis 1974**	**1975-1976**	**1977**	**1978-1980**	**1981-1982**	**1983**
1	Effektive Dosis	9,0 mSv	0,30 mSv	0,262 mSv	0,091 mSv	0,031 mSv	0,0037 mSv
2	Keimdrüsen	10,0 mSv	1,05 mSv	1,05 mSv	0,1403 mSv	0,140	0,0140 mSv

3.10 AN/FPS-89

Die ersten Messprotokolle zur Röntgenstörstrahlung der verschiedenen Komponenten des AN/FPS-89 stammen aus dem Jahr 1974. Die darin angegebenen Werte wurden ab 1975 zugrunde gelegt. Für die davorliegende Nutzungszeit des AN/FPS-89 wurden Messwerte an den entsprechenden Hauptkomponenten des Höhenmessradars AN/MPS-14 (Vorgängermodell) herangezogen, dessen Sender, Modulator und Hochspannungsnetzteil technisch mit denen des AN/FPS-89 identisch waren.

Gegenüber den Ortsdosisleistungswerten des AN/MPS-14 wurden beim AN/FPS-89 niedrigere Ortsdosisleistungswerte beobachtet.

Im Ergebnis wurden für das AN/FPS-89 dieselben Werte der Ortsdosisleistung zugrunde gelegt, wie für das AN/FPS-6. Die für die verschiedenen Nutzungsphasen ermittelten Werte der Teilkörper und Organdosiswerte (Tabelle A9) gelten für das AN/FPS-89 in gleicher Weise.

3.11 Höhenmessradargerät S-244

Das Ergebnis der Arbeitsplatzuntersuchung am Sender SR-1000 des Höhenmessradargerätes S-244 waren mögliche Exposition durch regelmäßige Arbeiten einerseits, für die eine monatliche Dosis angegeben wurde, und seltener und somit unregelmäßig anfallende Arbeiten, für die nur eine auf ein Beschäftigungsjahr bezogene Dosisangabe sinnvoll ist.

Für die hier anzustellende Dosisberechnung wird auf diese Unterteilung verzichtete und es wird in vereinfachender Weise die Jahresdosis zu 1/12 zur maximalen monatlichen Ortsdosis der regelmäßigen Arbeiten addiert. Damit ergeben sich die in Tabelle 32 angegebene Dosiswerte, wobei wegen technischer Umrüstungen bei S-244 zwei Nutzungsphasen zu unterscheiden sind

Tabelle 32: Maximalwerte der monatlichen Dosis an Arbeitsplätzen/Aufenthaltspunkten am Sender SR-1000 des Höhenmessradargerätes S-244

**Nr**	**Exponierte Körperteile**	**Hoch-spannung**	**Maximale monatliche Ortsdosis**		
			**bis 1973**	**ab 1974**	
1	Körperstamm in 1 m Höhe	45 kV	2,613 mSv	0,1125 mSv	
2	Kopf, Oberkörper	20 kV	0,01 mSv	0,01 mSv	
3	Beine	20 kV	7,53 mSv	0,67 mSv	

Die Einzelheiten der Dosisberechnung aus den Teilbeitragen sind in Tabelle A10 angegeben. In Tabelle 33 sind die Effektive Dosis und die Dosis für die Keimdrüsen angegeben, die in den beiden Nutzungszeiträumen pro Monat anzusetzen sind.

Tabelle 33: Werte der maximalen monatlichen Körper/Teilkörper-Dosiswerte bei Arbeiten am Sender SR-1000 des Höhenmessradargerätes S-244

**Nr**	**Dosis**	**Nutzungszeitraum**		
		**bis 1973**	**Ab 1974**	
1	Effektive Dosis	0,11 mSv	0,0273 mSv	
2	Keimdrüsen	0,33 mSv	0,143 mSv	

4 Zusammenfassung

Das Ergebnis der tätigkeitsbezogenen Dosisberechnung an den für den Anteil der Probanden, die bei der Bundeswehr gedient haben und für die eine tätigkeitsbedingte Exposition gegenüber Röntgenstörstrahlung vorgekommen ist, ist in Tabelle 33 angegeben.

Tabelle 33: Zusammenfassung der monatlichen Werte der effektiven Dosen bei Tätigkeiten an Radargeräten der Bundeswehr

**Nr**	**Radargerät**	**Tätigkeit**	**Monatliche Dosiswerte**		**Bemerkung**
			**Effektive Dosis**	**Organdosis Keimdrüsen**	
1	NASARR	Techniker	5,15 µSv	-	Medianwert
2	HAWK HPIR	Techniker	6,1 µSv	5,7 µSv	Medianwert
3	NIKE HIPAR	Techniker	24,9 µSv	41,1 µSv	Maximalwert
4	Deisswil	Techniker	0,01 µSv	0,043 µSv	Medianwert
5	AN/CPN-4	Techniker	180 µSv	240 µSv	Maximalwert
6	HADR	Techniker	31 µSv	-	Maximalwert bis 1984
7	AN/TPS-43	Techniker	bis 1981: 2100 µSv	300 µSv	Maximalwert
8			1982-1984: 1100 µSv	170 µSv	Maximalwert
9			ab 1985: 80 µSv	100 µSv	Maximalwert
10	AN/FPS-7E	Techniker	bis 1977: 158 µSv	276 µSv	Maximalwert
11			1977-1981: 141 µSv	241 µSv	Maximalwert
12			ab 1981: 22,7 µSv	-	Maximalwert
13	AN/FPS-6 AN/FPS-89	Techniker	bis 1974: 9,0 **m**Sv	10 **m**Sv	Maximalwert
14			1975-1976: 300 µSv	1050 µSv	Maximalwert
15			1977: 262 µSv	1050 µSv	Maximalwert
16	S-244	Techniker	bis 1973: 111 µSv	333 µSv	Maximalwert
17			ab 1974: 27,3 µSv	143 µSv	Maximalwert

Dr. A. Schirmer

Anhang 1: Hinweise zur Berechnung von Teilkörperdosiswerten und der Effektiven Dosis

Die Berechnung der Teilkörper- oder Organdosiswerte erfolgt anhand des einschlägigen Regelwerkes. Ausgangsgröße ist die während der Zeit, in der die Messungen durchgeführt wurden, gebräuchliche Photonen-Äquivalentdosis (H_x) , die mittels der Spannung der Röntgen-störstrahler in die für die Bewertung zu verwendende Umgebungs-Äquivalentdosis $H^*(10)$ (oder – bei Bewertung der Hautdosis- in die Richtungs-Äquivalentdosis $H'(0,07)$) anhand des in [2] angegeben Diagrammes (Abb. 3.1) umgerechnet wird. Mit der Bezeichnung H_0 für die Ortsdosis sind das die in den folgenden Tabellen angegeben Werte der monatlichen Ortsdosis an den Positionen der Teilkörperbereiche oder Organe.

In den weiteren Spalten der folgenden Tabellen werden die Bestrahlungsgeometrie und die durch die Photonenenergie bestimmten Konversionsfaktoren (ermittelt durch Ablesung der Diagramme in [3,4] angegeben. Die Konversionsfaktoren beruhen auf Berechnungen von aufrecht stehenden Körperphantomen, die durch parallele Strahlung exponiert werden. Einem kurzen Abstand des Körpers zur (punktförmigen) Quelle, also der Exposition durch divergente Strahlung wird durch sogenannte Korrektionsfaktoren k_k Rechnung getragen. Die in [3,4] angegeben Faktoren k_k werden hier übernommen. Weiterhin ist festzustellen, dass bei den untersuchten Arbeiten die Bestrahlung des aufrecht stehenden Menschen von vorn (ap-anterior-posterior) eine weitere Idealisierung darstellt. In [5] wurden Konversionsfaktoren für gebeugte Körperphantome bei Bestrahlung von oben (CRA-cranial) berechnet und verglichen. Es zeigt sich, dass bei einer Bestrahlung von oben der Konversionsfaktor kleiner ist als bei einer gebeugten Körperposition, bei der die Bestrahlung von CRA abweicht.

Übertragen auf die Bestrahlungsgeometrie mit gebeugter Körperhaltung an Radargeräte-schränken sind damit durch die Annäherung an die CRA-Bestrahlung in der Regel niedrigere Konversionsfaktoren zu erwarten[4]. Bei niedrigen Photonenenergien sind die Effekte ausgeprägter.

Die weiteren Spalten der folgenden Tabellen enthalten die schließlich berechneten monatlichen Teilkörper- oder Organdosiswerte.

Die Zahlenwerte wurden durchweg aus Tabellenkalkulationen übernommen und mit einer höheren Anzahl angegebener Stellen wird nicht unterstellt, dass die Genauigkeit erhöht ist. Die Ausgangswerte stellen ODL-Messungen dar, bei denen eine Unsicherheit von 15% nicht unterschritten wurde. Mindestens diese Unsicherheit ist für die Dosisangaben zu unterstellen.

---

[4] So ist bei weicher Röntgenstrahlung bei Bestrahlung (CRA-von oben) für bestimmte Organe (z.B. Speicheldrüse, Schilddrüse) ein erheblicher Abschwächungseffekt durch den darüber befindlichen Schädel zu erwarten.

Anhang 2: Berechnung von Teilkörper- und Organdosiswerten aus den ermittelten Werten der monatlichen Ortsdosen

Tabelle A1: Werte der monatlichen Teilkörper-/Organdosen für Radartechniker NASARR (Hochspannung des Röntgenstörstrahlers: 25 kV)

Radargerät		NASARR						
Techniker								
**Nr**	**Organ/Gewebe *T***	**Wich-tung w_T**	**Monatliche Ortsdosis H_0 [µSv]**	**Geo-metrie**	**Kon-versions-faktor f_k**	**Kor-rektion k_k**	**Organ-/Teilkörperdosis [µSv]**	
	1	2	3	4	5	6	7	
1	Knochenmark, rot	0,12	30,00	ap	0,147	1,10	4,85	
2	Dickdarm	0,12	0,00	ap	0,256	1,30	0,00	
3	Lunge	0,12	30,00	ap	0,175	1,10	5,78	
4	Magen	0,12	30,00	ap	0,250	1,50	11,25	
5	Brust	0,12	30,00	ap	0,333	1,50	14,99	
6	Keimdrüsen	0,08	0,00	ap	0,627	1,50	0,00	
7	Blase	0,04	0,00	ap	0,325	1,20	0,00	
8	Speiseröhre	0,04	30,00	ap	0,148	1,00	4,44	
9	Leber	0,04	30,00	ap	0,148	1,50	6,66	
10	Schilddrüse	0,04	30,00	ap	0,707	0,90	19,09	
11	Haut	0,01	2359,20		0,700	0,90	1486,30	
12	Knochenoberfläche	0,01	30,00	ap	0,297	1,00	8,92	
13	Gehirn	0,01	30,00	ap	0,028	0,70	0,58	
14	Speicheldrüsen	0,01	30,00	ap	0,178	0,60	3,20	
15	andere	0,12			0,171			
				ap	Effektive Dosis		20,62	

Anmerkung: Aus der Angabe in [3,4] für den Konversionsfaktor für die Effektive Dosis *E* zu $E/_{H^*(10)} = 0{,}31$ für die Bestrahlungsgeometrie ap a ergeben sich aus dem hierfür die inhomogene Bestrahlung ermittelten Wert für *E* für eine dafür angesetzte räumlich homogene Ortsdosis *H**(10) der Wert 66 µSv.

Tabelle A2: Maximalwerte der monatlichen Teilkörper-/Organdosen für Radartechniker am HPIR des Waffensystems HAWK (Hochspannung des Röntgenstörstrahlers 12 kV)

Radargerät		BASIC-HAWK, I-HAWK: HPIR (AN/MPQ-46)						
Radartechniker								
**Nr**	**Organ/Gewebe** ***T***	**Wich-tung** w_T	**Monatliche Ortsdosis** H_0 **[µSv]**	**Geo-metrie**	**Kon-versions-faktor** f_k	**Kor-rektion** k_k	**Organ-/Teilkörper-dosis [µSv]**	
	1	2	3	4	5	6	7	
1	Knochenmark, rot	0,12	89,52	pa	0,013	1,10	1,23	
2	Dickdarm	0,12	89,52	pa	0,013	1,30	1,49	
3	Lunge	0,12	89,52	pa	0,008	1,10	0,74	
4	Magen	0,12	89,52	pa	0,008	1,50	1,01	
5	Brust	0,12	89,52	pa	3,250	1,50	436,41	
6	Keimdrüsen	0,08	89,52	pa	0,671	1,50	90,06	
7	Blase	0,04	89,52	pa	0,128	1,20	13,70	
8	Speiseröhre	0,04	89,52	pa	0,005	1,00	0,45	
9	Leber	0,04	89,52	pa	0,005	1,50	0,67	
10	Schilddrüse	0,04	89,52	pa	0,225	0,90	18,13	
11	Haut	0,01	511,16		8,000	0,90	3680,35	
12	Knochenoberfläche	0,01	89,52	pa	0,067	1,00	5,97	
13	Gehirn	0,01	89,52	pa	0,000	0,70	0,02	
14	Speicheldrüsen	0,01	89,52	pa	0,013	0,60	0,67	
15	andere	0,12			0,073			
				ap	Effektive Dosis		98,3	

Anmerkung: Aus den Werten für den Konversionsfaktor für die Effektive Dosis E $f_{k\_E} = 0{,}70$ für pa ergibt sich aus dem für die inhomogene Bestrahlung ermittelte Wert für E für eine dafür anzusetzende räumlich homogene Ortsdosis $H^*(10) = \frac{E}{f_{k\_E}} = \frac{98{,}3\ \mu SvSv}{0{,}70} = 140\ \mu Sv$.

Tabelle A3: Werte der monatlichen Teilkörper-/Organdosen für einen Techniker der Instandsetzungsdienste an Radarsender der Rundsuchradargeräte LOPAR und HIPAR des Fla-Rak-Systems NIKE.

Radargerät			LOPAR					HIPAR					
Techniker							MAX -Wert					MAX-Wert	
**Nr**	**Organ/Gewebe** ***T***	**Wich-tung** w_T	**Monatliche Ortsdosis** H_0 **[µSv]**	**Geo-metrie**	**Kon-versions-faktor 36 keV** f_{k_AP}	**Kor-rektion** k_k	**Organ -/Teil-körper -dosis [µSv]**	**Monat-liche Ortsdosis** H_0 **28 keV [µSv]**	**Kon-versions-faktor 28 keV** f_{k_AP}	**Monat-liche Ortsdosis** H_0 **[µSv]**	**Kon-versions -faktor 200 keV** f_{k_A}	**Summe Organ-/Teil-körper-dosis [µSv]**	
	1	2	3	4	5	8	7						
1	Knochenmark, rot	0,12	0,00	ap	0,147	1,10	4,71	9,31	0,200	20,62	0,732	18,6	
2	Dickdarm	0,12	0,00	ap	0,256	1,30	12,54	9,31	0,368	20,62	0,872	27,8	
3	Lunge	0,12	0,00	ap	0,175	1,10	6,21	9,31	0,255	20,62	0,763	19,9	
4	Magen	0,12	0,00	ap	0,250	1,50	11,29	9,31	0,320	20,62	0,850	30,8	
5	Brust	0,12	0,00	ap	0,333	1,50	15,72	9,31	0,372	20,62	0,958	34,8	
6	Keimdrüsen	0,08	0,00	ap	0,627	1,50	39,43	9,31	0,727	20,62	1,000	41,1	
7	Blase	0,04	0,00	ap	0,325	1,20	16,40	9,31	0,397	20,62	0,974	28,5	
8	Speiseröhre	0,04	0,00	ap	0,148	1,00	4,95	9,31	0,195	20,62	0,775	17,8	
9	Leber	0,04	0,00	ap	0,148	1,50	8,26	9,31	0,245	20,62	0,790	27,8	
10	Schilddrüse	0,04	0,00	ap	0,707	0,90	27,52	9,31	0,773	20,62	1,061	26,2	
11	Haut	0,01	28,73		0,700	0,90	53,68	134,05	0,600	98,17	0,750	138,6	
12	Knochenoberfläche	0,01	0,00	ap	0,297	1,00	12,04	9,31	0,392	20,62	0,763	19,4	
13	Gehirn	0,01	0,00	ap	0,028	0,70	0,26	9,31	0,031	20,62	0,513	7,6	
14	Speicheldrüsen	0,01	0,00	ap	0,178	0,60	3,76	9,31	0,240	20,62	0,700	10,0	
15	andere	0,12	0,00		0,171				0,185		0,756		
	Effektive Dosis						0,18					24,9	

Tabelle A4: Werte der monatlichen Teilkörper-/Organdosen für einen Techniker der Instandsetzungsdienste am Radarsender des Feuerleitgerätes Deisswil. Dosisbestimmend ist die Clipperdiode (22 kV).

Radargerät		Deisswil						
Techniker							MAX-Wert	
**Nr**	**Organ/Gewebe** T	**Wich-tung** w_T	**Monatliche Ortsdosis** H_0 **[µSv]**	**Geo-metrie**	**Kon-versions-faktor** f_{k_AP}	**Kor-rektion** k_k	**Organ-/Teilkörper-dosis [µSv]**	
	1	2	3	4	5	8	7	
1	Knochenmark, rot	0,12	50,16	ap	0,085	1,10	4,71	
2	Dickdarm	0,12	50,16	ap	0,192	1,30	12,54	
3	Lunge	0,12	50,16	ap	0,113	1,10	6,21	
4	Magen	0,12	50,16	ap	0,150	1,50	11,29	
5	Brust	0,12	50,16	ap	0,209	1,50	15,72	
6	Keimdrüsen	0,08	50,16	ap	0,524	1,50	39,43	
7	Blase	0,04	50,16	ap	0,273	1,20	16,40	
8	Speiseröhre	0,04	50,16	ap	0,099	1,00	4,95	
9	Leber	0,04	50,16	ap	0,110	1,50	8,26	
10	Schilddrüse	0,04	50,16	ap	0,610	0,90	27,52	
11	Haut	0,01	74,56		0,800	0,90	53,68	
12	Knochenoberfläche	0,01	50,16	ap	0,240	1,00	12,04	
13	Gehirn	0,01	50,16	ap	0,008	0,70	0,26	
14	Speicheldrüsen	0,01	50,16	ap	0,125	0,60	3,76	
15	andere	0,12			0,085			
					Effektive Dosis		12,2	

Anmerkung: Aus dem Konversionsfaktor $f_{k\_E} = 0{,}24$ für die Effektive Dosis bei homogener Bestrahlungsrichtung ap ergibt sich aus dem hier für die inhomogene Bestrahlungsgeometrie ermittelten Wert der Effektiven Dosis E = 12,2 µSv als Dosiswert einer äquivalenten homogenen Bestrahlung $H^*(10)$ = 50,8 µSv.

Tabelle A5: Werte der monatlichen Teilkörper-/Organdosen für den Techniker des Landeanflugradargeräts AN/CPN-4. Maßgeblich ist das Thyratron des Rundsuchradars (16 kV).

Radargerät		AN/CPN-4						
Radartechniker							MAX-Wert	
**Nr**	**Organ/Gewebe** ***T***	**Wich-tung** w_T	**Monatliche Ortsdosis** H_0 **[mSv]**	**Geo-metrie**	**Kon-versions-faktor** f_{k_AP}	**Kor-rektion** k_k	**Organ-/Teilkörper-dosis [mSv]**	
1	Knochenmark, rot	0,12	0,53	ap	0,024	1,10	0,014	
2	Dickdarm	0,12	0,53	ap	0,051	1,30	0,035	
3	Lunge	0,12	0,53	ap	0,020	1,10	0,012	
4	Magen	0,12	0,53	ap	0,025	1,50	0,020	
5	Brust	0,12	0,53	ap	0,833	1,50	0,665	
6	Keimdrüsen	0,08	0,53	ap	0,300	1,50	0,240	
7	Blase	0,04	0,53	ap	0,150	1,20	0,096	
8	Speiseröhre	0,04	0,53	ap	0,025	1,00	0,013	
9	Leber	0,04	0,53	ap	0,020	1,50	0,016	
10	Schilddrüse	0,04	0,53	ap	0,366	0,90	0,175	
11	Haut	0,01	5,33		1,260	0,90	6,048	
12	Knochenoberfläche	0,01	0,53	ap	0,100	1,00	0,053	
13	Gehirn	0,01	0,53	ap	0,000	0,70	0,000	
14	Speicheldrüsen	0,01	0,53	ap	0,038	0,60	0,012	
15	andere	0,12			0,048			
					Effektive Dosis		0,182	

Anmerkung: Aus dem Konversionsfaktor $f_{k\_E} = 0{,}17$ für die Effektive Dosis bei homogener Bestrahlungsrichtung ap ergibt sich aus dem hier für die inhomogene Bestrahlungsgeometrie ermittelten Wert der Effektiven Dosis E = 0,182 mSv als Dosiswert für eine äquivalente homogene Bestrahlung H*(10) = 1,07 mSv.

Tabelle A6: Werte der monatlichen Teilkörper-/Organdosen für den Techniker des Luftraumüberwachungsradargerätes HADR bis 1984. Maßgeblich ist die Ortsdosisleistung an der TWT (45 kV)

Radargerät		HADR						
Radartechniker							MAX-Wert	
**Nr**	**Organ/Gewebe** ***T***	**Wich-tung** w_T	**Monatliche Ortsdosis** H_0 **[µSv]**	**Geo-metrie**	**Kon-versions-faktor** f_{k_AP}	**Kor-rektion** k_k	**Organ- /Teilkörper-dosis [µSv]**	
	1	2	3	4	5	8	7	
1	Knochenmark, rot	0,12	149,05	ap	0,147	1,10	24,10	
2	Dickdarm	0,12	0,00	ap	0,256	1,30	0,00	
3	Lunge	0,12	149,05	ap	0,175	1,10	28,69	
4	Magen	0,12	149,05	ap	0,250	1,50	55,89	
5	Brust	0,12	149,05	ap	0,333	1,50	74,45	
6	Keimdrüsen	0,08	0,00	ap	0,627	1,50	0,00	
7	Blase	0,04	0,00	ap	0,325	1,20	0,00	
8	Speiseröhre	0,04	149,05	ap	0,148	1,00	22,06	
9	Leber	0,04	149,05	ap	0,148	1,50	33,09	
10	Schilddrüse	0,04	149,05	ap	0,707	0,90	94,84	
11	Haut	0,01	381,30		0,700	0,90	240,22	
12	Knochenoberfläche	0,01	149,05	ap	0,297	1,00	44,33	
13	Gehirn	0,01	149,05	ap	0,028	0,70	2,87	
14	Speicheldrüsen	0,01	149,05	ap	0,178	0,60	15,92	
15	andere	0,12			0,171			
					Effektive Dosis		31,0	

Anmerkung: Aus dem Konversionsfaktor $f_{k\_E} = 0{,}617$ für die Effektive Dosis bei homogener Bestrahlungsrichtung ap ergibt sich aus dem hier für die inhomogene Bestrahlungsgeometrie ermittelten Wert der Effektiven Dosis E = 31 µSv als Dosiswert für eine äquivalente homogene Bestrahlung $H^*(10)$ = 50 µSv.

Tabelle A7: Werte der monatlichen Teilkörper-/Organdosen für den Techniker des Mobilen 3D-Radargerätes AN/TPS-43

	Radargerät	AN/TPS-43												
	Techniker													
							Nutzungszeitraum, technischer Zustand							
							**Bis 1981**		**1982-1984**		**ab 1985**		**Defekte Twystron-abschirmung**	
**Nr**	**Organ/Gewebe *T***	**Wich-tung w_T**	**Geo-me-trie**	**Kon-versions-faktor f_{k_AP}**	**Kor-rektion k_k**		**Monat-liche Orts-dosis H_0 [mSv]**	**Organ-/Teil-körper-dosis [mSv]**	**Monat-liche Ortsdosis H_0 [mSv]**	**Organ-/Teil-körper-dosis [mSv]**	**Monat-liche Ortsdosis H_0 [mSv]**	**Organ-/Teil-körper-dosis [mSv]**	**Monat-liche Ortsdo sis H_0 [mSv]**	**Organ-/Teil-körper-dosis [mSv]**
1	Knochenmark, rot	0,12	pa	0,866	1,10		3,53	3,363	0,16	0,153	0,142	0,1233	5,126	4,438
2	Dickdarm	0,12	pa	0,551	1,30		3,53	2,531	0,16	0,115	0,142	0,0785	5,126	2,826
3	Lunge	0,12	pa	0,750	1,10		3,53	2,914	0,16	0,133	0,142	0,1068	5,126	3,845
4	Magen	0,12	pa	0,513	1,50		3,53	2,715	0,16	0,124	0,142	0,0730	5,126	2,627
5	Brust	0,12	pa	0,541	1,50		3,53	2,866	0,16	0,131	0,142	0,0770	5,126	2,773
6	Keimdrüsen	0,08	pa	0,707	1,50		3,53	3,747	0,16	0,171	0,142	0,1007	5,126	3,626
7	Blase	0,04	pa	0,513	1,20		3,53	2,172	0,16	0,099	0,142	0,0730	5,126	2,627
8	Speiseröhre	0,04	pa	0,650	1,00		3,53	2,295	0,16	0,105	0,142	0,0926	5,126	3,332
9	Leber	0,04	pa	0,625	1,50		3,53	3,311	0,16	0,151	0,142	0,0890	5,126	3,204
10	Schilddrüse	0,04	pa	0,488	0,90		3,53	1,550	0,16	0,071	0,142	0,0695	5,126	2,501
11	Haut	0,01	pa	0,700	0,90		7,47	4,704	0,33	0,210	0,073	0,0512	10,249	7,174
12	Knochenoberfläche	0,01	pa	1,132	1,00		3,53	3,998	0,16	0,182	0,142	0,1612	5,126	5,803
13	Gehirn	0,01	pa	0,538	0,70		3,53	1,329	0,16	0,061	0,142	0,0765	5,126	2,755
14	Speicheldrüsen	0,01	pa	0,638	0,60		3,53	1,351	0,16	0,062	0,142	0,0908	5,126	3,268
15	andere	0,12	pa	0,631										
	Effektive Dosis							2,51		0,114		0,080		2,93

Anmerkung: Am AN/TPS-43 sind durch technische Maßnahmen bei der Dosisbetrachtung unterschiedliche Nutzungszeiträume zu berücksichtigen. Eine Reihe von hohen Messwerten der ODL wird mit Defekten der Twystron-Abschirmung asoziiert.

Tabelle A8: Werte der monatlichen Teilkörper-/Organdosen für den Techniker am 3D_Luftraumüberwachungsradar AN/FPS-7E

Radargerät		AN/FPS-7E										
Radartechniker							NUTZUNGSZEITRÄUME					
							Bis 1977		1977-1981		Ab 1981	
Nr	Organ/Gewebe T	Wich-tung w_T	Geo-metrie	Kon-versions-faktor f_{k_AP} 220 keV	Kon-versions-faktor f_{k_AP} 30 keV	Kor-rektion k_k	Monatl. Orts-dosis H_0 [mSv]	Organ-/Teil-körper-dosis [mSv]	Monatl. Orts-dosis H_0 [mSv]	Organ-/Teil-körper-dosis [mSv]	Monatl. Orts-dosis H_0 [mSv]	Monatl. Orts-dosis H_0 [mSv]
	1	2	4		5	8	7					
1	Knochenmark, rot	0,12	ap	0,720	0,220	1,10	0,271	0,081	0,240	0,073	0,028	0,0218
2	Dickdarm	0,12	ap	0,872	0,400	1,30	0,271	0,158	0,240	0,142	0,028	0,0312
3	Lunge	0,12	ap	0,765	0,275	1,10	0,271	0,097	0,240	0,088	0,028	0,0232
4	Magen	0,12	ap	0,850	0,350	1,50	0,271	0,163	0,240	0,147	0,028	0,0352
5	Brust	0,12	ap	0,958	0,458	1,50	0,271	0,207	0,240	0,186	0,028	0,0396
6	Keimdrüsen	0,08	ap	1,000	0,756	1,50	0,243	0,276	0,213	0,241	0,000	0,0000
7	Blase	0,04	ap	0,950	0,425	1,20	0,243	0,124	0,213	0,108	0,000	0,0000
8	Speiseröhre	0,04	ap	0,756	0,225	1,00	0,028	0,021	0,028	0,021	0,028	0,0208
9	Leber	0,04	ap	0,774	0,263	1,50	0,271	0,128	0,240	0,116	0,028	0,0320
10	Schilddrüse	0,04	ap	1,061	0,840	0,90	0,028	0,026	0,028	0,026	0,028	0,0263
11	Haut	0,01		0,800	0,600	0,90	6,969	3,788	6,126	3,332	0,142	0,1012
12	Knochenoberfläche	0,01	ap	0,763	0,480	1,00	0,271	0,138	0,240	0,123	0,028	0,0210
13	Gehirn	0,01	ap	0,525	0,050	0,70	0,028	0,010	0,028	0,010	0,028	0,0101
14	Speicheldrüsen	0,01	ap	0,695	0,250	0,60	0,028	0,011	0,028	0,011	0,028	0,0115
15	andere	0,12		0,738	0,244							
	Effektiv							0,158		0,141		0,0227

Tabelle A9: Werte der monatlichen Teilkörper-/Organdosen für den Techniker am 3D_Luftraumüberwachungsradar AN/FPS-7E

Radargerät		AN/FPS-6											
								**bis 1974**	**1975-1976**	**1977**	**1978-1980**	**1981-1982**	**ab 1983**
**Nr**	**Organ/Gewebe** ***T***	**Wich-tung** w_T	**Geo-metrie**	**Kon-versions-faktor** f_{k_AP} **65 keV**	**Kon-versions-faktor** f_{k_AP} **25 keV**	**Kor-rektion** k_k		**Organ-/Teil-körper-dosis [mSv]**	**Organ-/Teil-körper-dosis [mSv]**	**Organ-/Teil-körper-dosis [mSv]**	**Organ-/Teil-körper-dosis [mSv]**	**Organ-/Teil-körper-dosis [mSv]**	**Organ-/Teil-körper-dosis [mSv]**
1	Knochenmark, rot	0,12	ap	0,781	0,147	1,10		9,041	0,142	0,128	0,0958	0,01020	0,0042
2	Dickdarm	0,12	ap	0,846	0,256	1,30		6,715	0,372	0,372	0,0497	0,04965	0,0005
3	Lunge	0,12	ap	0,700	0,175	1,10		8,118	0,138	0,126	0,0870	0,01021	0,0038
4	Magen	0,12	ap	0,825	0,250	1,50		13,040	0,236	0,217	0,1413	0,01788	0,0063
5	Brust	0,12	ap	0,929	0,333	1,50		14,696	0,283	0,261	0,1608	0,02187	0,0072
6	Keimdrüsen	0,08	ap	1,061	0,627	1,50		10,003	1,052	1,052	0,1403	0,14032	0,0014
7	Blase	0,04	ap	0,925	0,325	1,20		6,851	0,436	0,436	0,0582	0,05819	0,0006
8	Speiseröhre	0,04	ap	0,659	0,148	1,00		6,942	0,114	0,104	0,0740	0,00836	0,0033
9	Leber	0,04	ap	0,753	0,148	1,50		11,886	0,188	0,171	0,1263	0,01363	0,0055
10	Schilddrüse	0,04	ap	1,159	0,707	0,90		11,098	0,270	0,142	0,0142	0,02224	0,0057
11	Haut	0,01		0,650	0,700	0,90		35,216	2,746	0,848	0,2846	0,25171	0,0288
12	Knochenoberfläche	0,01	ap	1,263	0,297	1,00		13,319	0,222	0,202	0,1423	0,01634	0,0063
13	Gehirn	0,01	ap	0,363	0,028	0,70		2,669	0,035	0,032	0,0277	0,00238	0,0012
14	Speicheldrüsen	0,01	ap	0,512	0,178	0,60		3,264	0,062	0,057	0,0354	0,01020	0,0016
15	andere	0,12		0,666	0,171								
	Effektiv							9,009	0,296	0,262	0,0912	0,03125	0,0037

Tabelle A10: Werte der maximalen monatlichen Teilkörper-/Organdosen für den Techniker am Höhenmessradar S-244

Radargerät	S-244														
Radartechniker															
								**Bis 1973**			**Ab 1974**			
**Nr**	**Organ/Gewebe** T	**Wich-tung** w_T	**Geo-metrie**	**Kon-versions-faktor** f_{k_AP} **45 keV**	**Kon-versions-faktor** f_{k_AP} **20 keV**	**Kor-rektion** k_k		**Organ-/Teil-körper-dosis [mSv] 45 keV**	**Organ-/Teil-körper-dosis [mSv] 20 keV**	**Organ-/Teil-körper-dosis [mSv] Summe**	**Organ-/Teil-körper-dosis [mSv] 45 keV**	**Organ-/Teil-körper-dosis [mSv] 20 keV**	**Organ-/Teil-körper-dosis [mSv] Summe**	
1	Knochenmark, rot	0,12	ap	0,147	0,0549	1,10		0,000	0,000	0,000	0,000	0,00033	0,00033	
2	Dickdarm	0,12	ap	0,256	0,1217	1,30		0,118	0,001	0,119	0,051	0,00000	0,05073	
3	Lunge	0,12	ap	0,175	0,0663	1,10		0,000	0,000	0,000	0,000	0,00040	0,00040	
4	Magen	0,12	ap	0,250	0,0875	1,50		0,000	0,001	0,001	0,000	0,00071	0,00071	
5	Brust	0,12	ap	0,333	0,5210	1,50		0,000	0,004	0,004	0,000	0,00424	0,00424	
6	Keimdrüsen	0,08	ap	0,627	0,4120	1,50		0,333	0,000	0,333	0,143	0,00000	0,14337	
7	Blase	0,04	ap	0,325	0,2113	1,20		0,138	0,000	0,138	0,059	0,00000	0,05945	
8	Speiseröhre	0,04	ap	0,148	0,0617	1,00		0,000	0,000	0,000	0,000	0,00033	0,00033	
9	Leber	0,04	ap	0,148	0,0649	1,50		0,000	0,001	0,001	0,000	0,00053	0,00053	
10	Schilddrüse	0,04	ap	0,707	0,4878	0,90		0,000	0,002	0,002	0,000	0,00238	0,00238	
11	Haut	0,01		0,700	1,0300	0,90		0,000	6,389	6,389	0,090	0,56845	0,65853	
12	Knochenoberfläche	0,01	ap	0,297	0,1700	1,00		0,000	0,001	0,001	0,000	0,00092	0,00092	
13	Gehirn	0,01	ap	0,028	0,0038	0,70		0,000	0,000	0,000	0,000	0,00001	0,00001	
14	Speicheldrüsen	0,01	ap	0,178	0,0813	0,60		0,000	0,000	0,000	0,000	0,00026	0,00026	
15	andere	0,12		0,171	0,0848									
	Effektiv									0,111			0,02734	
